# Identification of antibacterial constituents from *Rhododendron simsii* Planch with an activity-guided method

**DOI:** 10.3389/fphar.2024.1490335

**Published:** 2024-10-08

**Authors:** Yongji Lai, Yu-Ting Zhong, Yu Liang, Wei-Chen Chen, Qiuyan Liao, Mu Li, Pan Han, You-Sheng Cai, Fuqian Wang

**Affiliations:** ^1^ Department of Pharmacy, The Central Hospital of Wuhan, Tongji Medical College, Huazhong University of Science and Technology, Wuhan, China; ^2^ Key Laboratory of Combinatorial Biosynthesis and Drug Discovery, Ministry of Education and School of Pharmaceutical Sciences, Wuhan University, Wuhan, China; ^3^ Department of Medicament, College of Medicine, Tibet University, Lhasa, China; ^4^ Department of Pharmacy, Wuhan No.1 Hospital, Wuhan, China

**Keywords:** *Rhododendron simsii* Planch, diterpenoids, triterpenoids, absolute configuration, antibacterial

## Abstract

Bacterial infections and antibiotic resistance pose significant public health challenges globally. Natural products serve as valuable sources for discovering antimicrobial agents. *Rhododendron simsii* Planch, a folk medicine, is traditionally used to treat various inflammatory diseases. In this study, we investigated the antibacterial metabolites derived from *R. simsii* Planch. Rhodosimsiin A (1), bearing a 1,5-*seco*-1,6 and 3,6-epoxy grayanane diterpene skeleton, representing a novel 5/6/7/6/5 pentacyclic ring system, and 3*β*,16*α*-dihydroxy-6*β*-ethoxy-14*β*-acetoxy-grayan-1(5)-ene-10-one (4), which represents the first example of the degradation of C-20 and carbonylation in C-10 diterpenoid, together with two new grayanane diterpenes (2−3), three new triterpenes (13−15), and known analogs (5−12, 16−30), were isolated from the leaves of *R. simsii* Planch by using the bioassay-guided method. Their structures were elucidated by comprehensive spectroscopic analyses, and absolute configurations were established by single-crystal X-ray diffraction and calculated ECD spectra. Compounds 14, 15, 18, 20, 27, 28, and 30 exhibited potent antibacterial activity with an MIC_50_ of 1.4–24.3 *μ*g/mL against *Staphylococcus aureus*. The findings of this research indicate that secondary metabolites derived from *R*. *simsii* Planch are promising natural antimicrobial candidates.

## Graphical Abstract

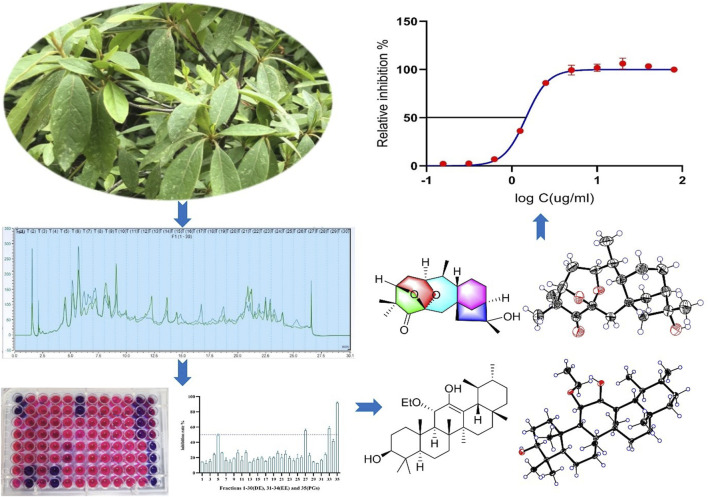

## 1 Introduction

Natural products (NPs) have played a critical role in drug discovery (Newman and Cragg, 2016). Chemists continue to use natural agents as prototypes to develop more effective and less toxic medicines.

Indigenous medicines and traditional medicines are well recognized as a unique source for the discovery of structurally novel and biologically active secondary metabolites. The *Rhododendron* genus, a member of the Ericaceae family, has rich resources and is distributed widely in China (Huang et al., 2018). Previously, many *Rhododendron* plants, such as *Rhododendron latoucheae* and *Rhododendron molle* G. Don, have been used to treat bronchitis, cough, rheumatoid arthritis, pain, and skin ailments (Popescu and Kopp, 2013; Liu et al., 2018; Luo et al., 2023) and applied in anti-rheumatoid arthritis therapy as folk medicine (He et al., 2021). *Rhododendron simsii* Planch, also known as Ying-shan-hong, is one of the folk medicines recorded in the “Dictionary of Chinese Materia Medica” and “Compendium of Materia Medica” for treating rheumatic diseases. It has also been used by many ethnic communities in China to control cough, pain, and various inflammatory and immune-related diseases such as rheumatoid arthritis (Nanjing University of Traditional Chinese Medicine, 2006).

Diterpenoids, triterpenoids, and flavonoids, displaying diverse biological activities, constitute the main chemical components of Rhododendraceae. Grayanane diterpenoids, featuring a unique 5/7/6/5 tetracyclic carbon skeleton, are exclusively found in Ericaceae plants (Wang et al., 2014). Their complex polycyclic carbon skeleton and extensive bioactivities, such as antinociceptive (Li et al., 2003), PTP1B inhibitory activity (Zhou et al., 2017), immunomodulatory (Zhang et al., 2013), anti-inflammatory (Li et al., 2019; Zhou et al., 2018), and antithrombotics effects (Zheng et al., 2023), have attracted considerable attention from organic synthesis chemists (Zhu et al., 2023).

**FIGURE 1:**
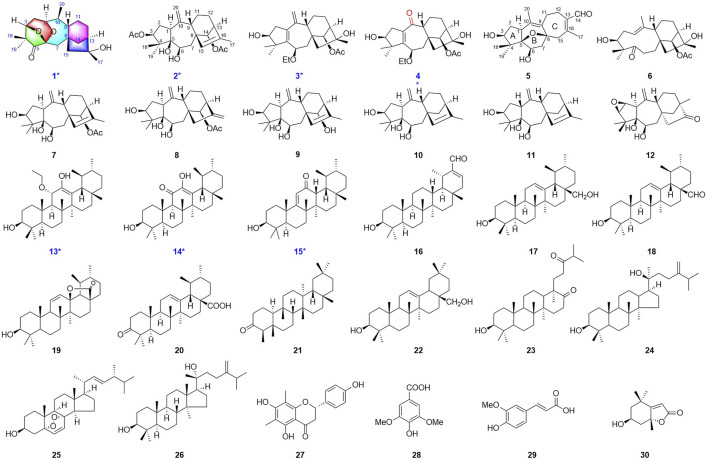
Structures of compounds 1−30.

These attractive, valuable examples from Ericaceae and the traditional application of *R. simsii* Planch propel the continuous investigation of antibacterial constituents for drug discovery. In our ongoing research on *R. simsii* Planch, we obtained one novel diterpenoid rhodosimsiin A (1), with an unprecedented pentacyclic skeleton; three new diterpenoids 3-AcO-grayanotoxin IX (2), 3*β*,16*α*-dihydroxy-6*β*-ethoxy-14*β*-acetoxy-grayan-1(5),10(20)-diene (3), and 3*β*,16*α*-dihydroxy-6*β*-ethoxy-14*β*-acetoxy-grayan-1(5)-ene-10-one (4); and three new triterpenes 11*α*-methoxyurs-12-ene-3*β*,12-diol (13), 3*β*,12-dihydroxyurs-12-en-11-one (14), and 3*β*-hydroxy-12-oxours-11-ene (15). In addition, related biogenetic analogs rhodauricanol A (5) (Feng et al., 2023), dauricanol E (6) (Feng et al., 2023), grayanotoxin IX (7) (Zhang et al., 2005; Zhou et al., 2014), grayanotoxin IX (8) (Chen et al., 2018), grayanotoxin VII (9) (Zheng et al., 2019; Li Y. et al., 2015), grayanotoxin XIX (10) (Sakakibara et al., 1980; Furusaki et al., 1981), grayathol A (11) (Furusaki et al., 1979), rhododecorumin V (12) (Zhu et al., 2018), 3*β*-hydroxy-taraxaster-20-ene-30-aldehyde (16) (Huang et al., 2011), 3*β*,28-dihydroxyurs-12-ene (17) (El-Seedi, 2005), ursaldehyde (18) (Ngo et al., 2018), 3-hydroxy-13,28-epoxyurs-11-en-28-one (19) (Musayeib et al., 2013), ursonic acid (20) (Poehland et al., 1987), friedelin (21) (Ageta et al., 1995), erythrodiol (22) (Kagawa et al., 1998), scabranol (23) (Li W. et al., 2015), foliasalacin A4 (24) (Yoshikawa et al., 2008), (22*E*)-5*α*,8*α*-epidioxyergosta-6,22-dien-3*β*-ol (25) (Hybelbauerová et al., 2008), euphorfistrine C (26) (Wei et al., 2021), farrerol (27) (Li et al., 2014), syringic acid (28) (Long et al., 2022), ferulic acid (29) (Rho and Yoon, 2017), and loliolide (30) (Shinde et al., 2007) were also obtained (Figure 1). Rhodosimsiin A (1) possesses an unprecedented 5/6/7/6/5 pentacyclic skeleton featuring a 1,5-*seco*-1,6 and 3,6-epoxy grayanane, and 3*β*,16*α*-dihydroxy-6*β*-ethoxy-14*β*-acetoxy-grayan-1(5)-ene-10-one (4) is the first example of the degradation of C-20 and carbonylation in C-10 diterpenoid. Here, we report the isolation, structure elucidation, and antibacterial evaluation of compounds 1–30.

## 2 Materials and methods

### 2.1 General experimental procedures

HRESI-MS data were recorded on a Thermo Fisher Scientific LTQ Orbitrap XL mass instrument spectrometer. NMR spectra were obtained using a Bruker Avance 400/600 NMR spectrometer. CD spectra were recorded using a JASCO J-815 CD spectrometer. X-ray diffraction data were collected using a Bruker SMART APEX-II CCD diffractometer. MPLC was carried out using an EZ PLUS 100D chromatography system (Lisure Science Co., Ltd., Suzhou). Preparative and semi-preparative HPLC was carried out using a Thermo Fisher Scientific Ultimate 3000 system using a C_18_ column (YMC−Pack ODS−AQ, 5 μm, 250 × 10 mm). The sample drying process was finalized using a speed Vac 2030 (Thermo Fisher Scientific). TLC was carried out using glass-precoated silica gel GF254 (Yantai Chemical Industry Research Institute) and visualized under UV light. Silica gel (Qingdao Haiyang Chemical Co., Ltd.), ODS, Sephadex LH−20, and MCI (YMC Co., Ltd.) were used for column chromatography.

### 2.2 Plant material and extraction

The procedure for specimen collection and extraction is similar to that in our prior research (Wang et al., 2023). The leaves of *R. simsii* Planch were collected from Shennongjia in Hubei Province, China, and identified by Prof. Xincai Hao (Hubei University of Medicine, Shiyan, China). A voucher specimen (No. Pharm-202006) was deposited at the Department of Pharmacy, Wuhan No.1 Hospital. The air-dried leaves (20 kg) were extracted with 95% aqueous EtOH ( 2 days each, 50 L × 3 times) at room temperature. The filtrates were combined and concentrated under vacuum to afford the crude extract, which was suspended in H_2_O (6 L) and then successively re-extracted with chloroform and ethyl acetate (5 L × 4, each). The solvent was concentrated under vacuum separately to afford 516 g (dichloromethane extract, DE) and 230 g (EtOAc extract, EE) residue. The obtained extract was stored in a refrigerator at 4°C until further use.

### 2.3 Bioassay-guided isolation

The results of the preliminary antibacterial activity evaluation showed that the subfractions 3–4 of DE and subfractions 4 and 27 of EE exhibited obvious antibacterial activity against *Staphylococcus aureus* at 100 *μ*g/m (SI). Therefore, the active fractions were selected for further antibacterial component separation, and finally, 30 compounds were obtained after isolation and purification processes.

The DE (114 g) was subjected to silica gel column chromatography (CC) and eluted with a petroleum ether–acetone (PE–AC) gradient (20:1; 10:1; 5:1; 2:1; and 0:1, each 500 mL) to afford subfractions (Fr.1–Fr.5). Fr.2 was subjected to an MCI gel (90% MeOH), followed by Sephadex LH-20 column chromatography (CH_2_Cl_2_/CH_3_OH 1:1) and was then purified through recrystallization to obtain friedelin (21, 163.2 mg). The decolorized Fr.3 from MCI (100% MeOH) was chromatographed into two subfractions (Fr.3.1 and Fr.3.2) using a silica gel column chromatograph (CC). Fr.3.1 was further separated using an MPLC system with an ODS column and semipreparative HPLC (YMC−Pack ODS−AQ, 5 µ, 250 × 10 mm, 2 mL/min) to afford 11*α*-methoxyurs-12-ene-3*β*,12-diol (13, 44.6 mg, *t*_R_ = 40 min, ACN-H_2_O, 100:0), 3*β*-hydroxy-taraxaster-20-ene-30-aldehyde (16, 8.1 mg, *t*_R_ = 28 min, MeOH/H_2_O, 82:18), ursaldehyde (18, 5.9 mg, *t*_R_ = 32 min, MeOH/H_2_O, 80:20), 3*β*,12-dihydroxyurs-12-en-11-one (14, 8.4 mg, *t*_R_ = 39 min, MeOH/H_2_O, 80:20), foliasalacin A4 (24, 5.5 mg, *t*_R_ = 43 min, MeOH/H_2_O, 75:25), and (22*E*)-5*α*,8*α*-epidioxyergosta-6,22-dien-3*β*-ol (25, 7.9 mg, Sephadex LH-20, MeOH). Fr.3.2 was treated in the same manner to obtain 3*β*,28-dihydroxyurs-12-ene (17, 7.4 mg, *t*_R_ = 26 min, ACN-H_2_O, 100:0), ursonic acid (20, 5.0 mg, *t*_R_ = 37 min, ACN-H_2_O, 86:14), erythrodiol (22, 8.0 mg, *t*_R_ = 33 min, ACN-H_2_O, 100:0), and euphorfistrine C (26, 5.3 mg, *t*_R_ = 53 min, ACN-H_2_O, 90:10). Fr.4 was chromatographed using the MCI gel eluted with MeOH and was then fractionated using the silica gel CC eluted with the CH_2_Cl_2_–MeOH gradient (40:1; 20:1; and 10:1, each 500 mL) to afford two fractions (Fr.4.1 and Fr.4.2). Fr.4.1 was then resubjected to the MPLC system and semipreparative HPLC (YMC−Pack ODS−AQ, 5 µ, 250 × 10 mm, 2 mL/min) to afford 3-AcO-grayanotoxin IX (2, 2.4 mg, *t*_R_ = 48 min, MeOH/H_2_O, 57:43), grayanotoxin XIX (10, 141.5 mg, *t*_R_ = 41 min, MeOH/H_2_O, 57:43), grayathol A (11, 4.6 mg, *t*_R_ = 30 min, MeOH/H_2_O, 63:37), rhododecorumin V (12, 3.2 mg, *t*_R_ = 40 min, MeOH/H_2_O, 46:54), 3-hydroxy-13,28-epoxyurs-11-en-28-one (19, 4.6 mg, *t*_R_ = 17 min, MeOH/H_2_O, 90:10), scabranol (23, 5.9 mg, *t*_R_ = 15 min, MeOH/H_2_O, 90:10), and 3*β*-hydroxy-12-oxours-11-ene (15, 3.4 mg, *t*_R_ = 43 min, MeOH/H_2_O, 88:12). Fr.4.2 was purified using the Sephadex LH-20 CC, followed by semipreparative HPLC (YMC−Pack ODS−AQ, 5 µ, 250 × 10 mm, 2 mL/min) to yield rhodauricanol A (5, 4.0 mg, *t*_R_ = 28 min, MeOH/H_2_O, 52:48), 3*β*,16*α*-dihydroxy-6*β*-ethoxy-14*β*-acetoxy-grayan-1(5),10(20)-diene (3, 5.8 mg, *t*_R_ = 42 min, MeOH/H_2_O, 55:45), 3*β*,16*α*-dihydroxy-6*β*-ethoxy-14*β*-acetoxy-grayan-1(5)-ene-10-one (4, 2.0 mg, *t*_R_ = 40 min, MeOH/H_2_O, 55:45), dauricanol E (6, 4.2 mg, *t*_R_ = 37 min, MeOH/H_2_O, 52:48), grayanotoxin IX (7, 166.0 mg, *t*_R_ = 20 min, MeOH/H_2_O, 65:35), grayanotoxin IX (8, 68.6 mg, *t*_R_ = 28 min, MeOH/H_2_O, 65:35), grayanotoxin VII (9, 14.4 mg, *t*_R_ = 40 min, MeOH/H_2_O, 52:48), and loliolide (30, 56.4 mg, recrystallization).

The EE fraction (46 g) was subjected to an MPLC system with an ODS column (25%−100% MeOH) to yield 30 subfractions (Fr.1−Fr.30). After the active fraction screening process, subfractions 4 and 27 were selected for further separation. Fr.4 was then chromatographed using the MCI CC (MeOH 100%) and was purified by semipreparative HPLC (YMC−Pack ODS−AQ, 5 µ, 250 × 10 mm, 2 mL/min) to yield rhodosimsiin A (1, 2.2 mg, *t*_R_ = 14.3 min, MeOH/H_2_O, 80:20), syringic acid (28, 46.6 mg, *t*_R_ = 28 min, MeOH/H_2_O, 25:75, 0.2% FA), and ferulic acid (29, 7.9 mg, *t*_R_ = 36 min, MeOH/H_2_O, 25:75, 0.2% FA). Farrerol (27, 1,075 mg) was derived from Fr.27 using the Sephadex LH-20 column chromatograph (MeOH).

*Rhodosimsiin A* (1): colorless crystals; [*α*]^20^_D_ + 22.5 (*c* 0.07 g/100 mL, MeOH); CD (*c* 1.99 × 10^−3^ M, MeOH) *λ*_max_(Δ*ε*) 207 (−4.56), 304 (+2.39) nm; ^1^H and ^13^C NMR data, Tables 1, 2; HR-MS *m/z* 335.2208 [M + H]^+^ (calcd for C_20_H_31_O_4_^+^, 335.2217).

**TABLE 1:** ^1^H (*δ* in ppm, *J* in Hz) NMR data of 1–4 in CD_3_OD (400 MHz).

No.	1	2	3	4
1	4.39, t (9.2)	2.82, dd (12.0, 7.8)		
2	2.07, dd (14.9, 9.3)	1.95, overlap	2.19, dd (16.0, 3.5)	2.30, d (16.6)
	2.41, dd (14.9, 9.3)	2.22, dt (15.3, 7.8)	2.98, ddd (16.0, 6.2, 1.3)	2.92, dd (16.6, 6.1)
3	4.12, d (9.0)	4.85, dd (7.8, 5.7)	3.81, dd (6.1, 3.4)	3.85, dd (6.5), overlap
6		3.66, dd (10.1, 2.3)	3.98, d (6.3)	4.18, d (6.1)
7	1.67, d (14.6)	1.56, overlap	1.52, d (15.1)	1.72, d (15.4)
	2.05, d (14.6)	1.95, overlap	2.44, dd (15.1, 6.5)	2.63, dd (15.4, 6.0)
9	1.99, m	2.70, m	3.21, d (7.3)	3.85, d (6.5), overlap
10	2.55, m			
11	1.58, overlap	1.58, overlap	1.86, overlap	1.53, m
	1.67, overlap	1.95, overlap	1.98, m	1.80, m
12	1.36, m	1.58, overlap	1.63, m	1.75, m
	1.59, overlap	1.90, overlap	1.84, overlap	1.95, m
13	1.83, m	2.29, d (6.7)	2.13, m	2.19, m
14	1.82, m	5.51, s	5.01, s	4.63, s
	2.20, d (10.9)			
15	1.61, m	5.25, s	2.21, br s	2.21, m
	1.98, overlap			
17	1.32, s	1.73, d (1.6)	1.42, s	1.41, s
18	1.19, s	1.09, s	1.08, s	1.16, s
19	1.14, s	1.13, s	0.98, s	1.02, s
20	0.96, d (6.9)	4.93, overlap	5.20, d (12.3)	
		5.05, s		
3-OAc		2.06, s		
6-OEt			3.50, m; 3.76, dd (8.8, 7.0)	3.54, m; 3.76, m
			1.23, t	1.24, t (7.0)
14-OAc		2.08, s	2.04, s	2.03, s

The “m” means multiplet signals.

**TABLE 2:** ^13^C (*δ* in ppm) NMR data of 1–4 in CD_3_OD (150 MHz).

No.	1	2	3	4
1	73.3	43.1	140.6	138.3
2	26.9	36.3	42.2	37.7
3	79.7	83.1	79.8	77.7
4	44.9	50.5	52.4	52.1
5	209.0	82.6	146.8	162.1
6	100.7	70.9	73.9	71.8
7	44.2	37.0	38.8	38.5
8	46.8	51.2	49.6	46.1
9	48.5	50.8	50.9	55.4
10	38.7	150.4	150.1	203.2
11	25.1	27.3	27.8	19.4
12	23.8	23.2	24.8	25.4
13	51.0	49.8	53.0	56.0
14	41.6	82.4	86.9	85.6
15	58.0	134.9	57.3	56.4
16	79.1	142.9	81.9	80.0
17	23.1	14.8	24.8	23.2
18	22.5	19.2	20.5	18.1
19	26.2	26.4	25.2	23.1
20	17.7	114.7	113.8	
3-OAc		21.1		
		172.9		
6-OEt			66.2	65.1
			16.1	14.6
14-OAc		21.0	21.2	19.7
		173.4	172.8	171.1

Grayanotoxin IX (2): white, amorphous powder; [*α*]^20^_D_ –1.5 (*c* 0.07 g/100 mL, MeOH); CD (*c* 1.59 × 10^−3^ M, MeOH) *λ*_max_(Δ*ε*) 211 (−27.8), 237 (+0.20) nm; ^1^H and ^13^C NMR data, Tables 1, 2; HR-MS *m/z* 419.2420 [M + H]^+^ (calcd for C_24_H_35_O_6_^+^, 419.2428).

3*β*,16*α*-dihydroxy-6*β*-ethoxy-14*β*-acetoxy-grayan-1(5),10(20)-diene (3): white, amorphous powder; [*α*]^20^_D_ + 133.0 (*c* 0.03 g/100 mL, MeOH); CD (*c* 0.82 × 10^−3^ M, MeOH) *λ*_max_(Δ*ε*) 217 (+6.22), 246 (+44.79) nm; ^1^H and ^13^C NMR data, Tables 1, 2; HR-MS *m/z* 405.2623 [M + H]^+^ (calcd for C_24_H_37_O_5_^+^, 405.2636).

3*β*,16*α*-dihydroxy-6*β*-ethoxy-14*β*-acetoxy-grayan-1(5)-ene-10-one (4): white, amorphous powder; [*α*]^20^_D_ + 36.0 (*c* 0.03 g/100 mL, MeOH); CD (*c* 0.81 × 10^−3^ M, MeOH) *λ*_max_(Δ*ε*) 206 (+6.46), 229 (−0.47), 257 (+10.29), 339 (−2.18) nm; ^1^H and ^13^C NMR data, Tables 1, 2; HR-MS *m/z* 407.2421 [M + H]^+^ (calcd for C_23_H_35_O_6_^+^, 407.2428).

11*α*-methoxyurs-12-ene-3*β*,12-diol (13): colorless crystals; [*α*]^20^_D_ + 22.6 (*c* 0.17 g/100 mL, MeOH); CD (*c* 3.53 × 10^−3^ M, MeOH) *λ*_max_(Δ*ε*) 209 (+5.25), 236 (−0.47), 285 (−0.59) nm; ^1^H and ^13^C NMR data, Tables 3, 4; HR-MS *m/z* 509.3965 [M + Na]^+^ (calcd for C_32_H_54_NaO_3_^+^, 509.3963).

**TABLE 3:** ^1^H (*δ* in ppm, *J* in Hz) NMR data of 13−15 in CDCl_3_ (400 MHz).

No.	13	14	15
1	1.27, overlap	1.07, overlap	1.41, overlap
	2.12, dt (13.6, 3.6)	2.75, dt (13.6, 3.6)	2.01, dt (13.3, 3.5)
2	1.53, m	1.66, m	1.67, m
	1.65, qd (13.1, 3.4)		1.76, m
3	3.17, dd (11.8, 4.6)	3.24, dd (11.0, 5.3)	3.17, dd (11.8, 4.5)
5	0.79, overlap	0.72, d (10.4)	0.94, overlap
6	1.41, overlap	1.43, overlap	1.63, m
	1.58, m	1.60, overlap	1.71, overlap
7	1.34, overlap	1.44, overlap	1.46, overlap
	1.50, m	1.67, overlap	1.71, overlap
9	1.88, d (9.4)	2.47, s	
11	4.08, d (9.4)		5.92, s
12		6.28 brs, OH	
13			2.85, d (3.9)
15	1.80, dt (13.5, 5.0)	1.90, dt (13.6, 5.0)	1.81, dt (13.5, 4.6)
16	0.82, overlap	0.94, overlap	0.92, overlap
	0.99, overlap	1.66, overlap	1.06, overlap
	2.06, dt (13.5, 5.0)	2.09, dt (13.6, 5.0)	1.95, dt (13.5, 4.6)
18	2.27, d (11.1)	2.44, dd (11.3, 1.8)	2.08, dd (11.2, 3.1)
19	1.36, overlap	1.41, overlap	1.47, overlap
20	1.01, m	1.06, overlap	1.10, overlap
21	1.29, overlap	1.27, overlap	1.23, overlap
	1.40, overlap	1.44, overlap	1.44, overlap
22	1.43, overlap	1.47, overlap	1.42, overlap
23	0.99, s	1.01, s	1.05, s
24	0.80, s, overlap	0.81, s	0.84, s
25	1.11, s	1.15, s	1.21, s
26	1.08, s	1.17, s	1.29, s
27	1.21, s	1.35, s	1.09, s
28	0.80, s, overlap	0.83, s	0.99, s
29	0.94, d, overlap	0.79, d (6.6)	0.69, d, (6.7)
30	0.93, d, overlap	0.92, d (6.5)	0.87, d, (6.4)
31	3.40, m; 3.68, m		
32	1.09, t (6.88)		

**TABLE 4:** ^13^C (*δ* in ppm) NMR data for 13–15 in CDCl_3_ (150 MHz).

No.	13	14	15	No.	13	14	15
1	40.6	39.4	36.4	17	34.3	33.6	35.0
2	28.2	27.7	27.7	18	48.4	49.1	47.9
3	79.5	78.8	78.3	19	42.4	41.0	39.9
4	40.3	39.3	39.3	20	41.0	39.4	39.5
5	56.9	55.1	50.0	21	32.6	31.4	31.9
6	19.6	17.7	18.1	22	43.1	41.3	41.8
7	35.0	33.2	33.1	23	28.9	28.3	28.3
8	44.2	45.7	45.7	24	16.6	15.8	15.8
9	50.8	59.9	179.4	25	17.3	16.8	24.4
10	39.6	37.4	40.2	26	18.8	18.7	24.8
11	77.9	195.5	123.5	27	24.2	21.2	19.7
12	142.4	144.6	203.2	28	29.3	29.0	28.5
13	118.0	134.5	48.2	29	21.8	16.7	20.4
14	41.6	41.8	41.1	30	17.5	21.2	21.2
15	28.7	27.5	26.9	31	62.0		
16	29.0	27.4	27.4	32	15.9		

3*β*,12-dihydroxyurs-12-en-11-one (14): white, amorphous powder; [*α*]^20^_D_ +146.0 (*c* 0.10 g/100 mL, MeOH); CD (*c* 2.19 × 10^−3^ M, MeOH) *λ*_max_(Δ*ε*) 210 (+4.75), 233 (−1.91), 288 (+9.97) nm; ^1^H and ^13^C NMR data, Tables 3, 4; HR-MS *m/z* 457.3676 [M + H]^+^ (calcd for C_30_H_49_O_3_^+^, 457.3505).

3*β*-hydroxy-12-oxours-11-ene (15): colorless crystals; [*α*]^20^_D_ + 107.2 (*c* 0.08 g/100 mL, MeOH); CD (*c* 2.19 × 10^−3^ M, MeOH) *λ*_max_(Δ*ε*) 239 (+10.46), 268 (−2.99), 337 (+5.23) nm; ^1^H and ^13^C NMR data, Tables 3, 4; HR-MS *m/z* 441.3727 [M + H]^+^ (calcd for C_30_H_49_O_2_^+^, 441.3728).

### 2.4 X-ray crystallographic analysis

The crystals were selected, and the data were collected on a Rigaku XtaLAB SynergyCustom HyPix-Arc 150 diffractometer (Cu-K*α* radiation, *λ* = 1.54184 Å). The crystals were maintained at 100.00 K during data collection. Using Olex2 (Dolomanov et al., 2009), the structure was solved using SHELTXT (Sheldrick, 2015a, A71) structure solution program using intrinsic phasing and refined with the ShELXLT (Sheldrick, 2015b, C71) refinement package using least squares minimization. Crystallographic data of 1 (CCDC 2379395), 5 (CCDC 2379394), 7 (CCDC 2379401), 8 (CCDC 2379400), 9 (CCDC 2379396), 12 (CCDC 2379397), 13 (CCDC 2379398), and 15 (CCDC 2379399) were deposited in the Cambridge Crystallographic Data Center.

### 2.5 Antibacterial assays

The MIC_50_ values were determined by a standardized microdilution method according to CLSI Performance Standards for Antimicrobial Susceptibility Testing 2009. In brief, bacterium inocula were added to each well in a 96-well plate, and the inoculum was standardized to approximately 5 × 10^5^ CFU/mL. Then, twofold serial dilutions of test compounds (80 μg/mL) afforded the final concentrations in a series of wells. Ceftazidime and penicillin G sodium salt (Biosharp) were used as positive controls, and after incubation at 37°C for 24 h, the OD_600_ was measured using a microplate reader. MIC_50_ values were calculated using GraphPad Prism 8.0.

## 3 Results

### 3.1 Structure elucidation

Rhodosimsiin A (1) was isolated as a white powder with the molecular formula of C_20_H_30_O_4_ (*m/z* 335.2208 [M + H]^+^, calcd for C_20_H_31_O_4_, 335.2217) that was established by HR-MS and required six degrees of unsaturation. The ^1^H NMR data (Table 1) of 1 displayed 4 methyls (*δ*_H_ 0.97, 1.14, 1.19, and 1.32). The ^13^C NMR, HMBC, and HSQC spectra showed 20 carbons, including 4 methyls, 6 methylenes, 5 methines, and 1 carbonyl (*δ*_C_ 209.0).

**FIGURE 2:**
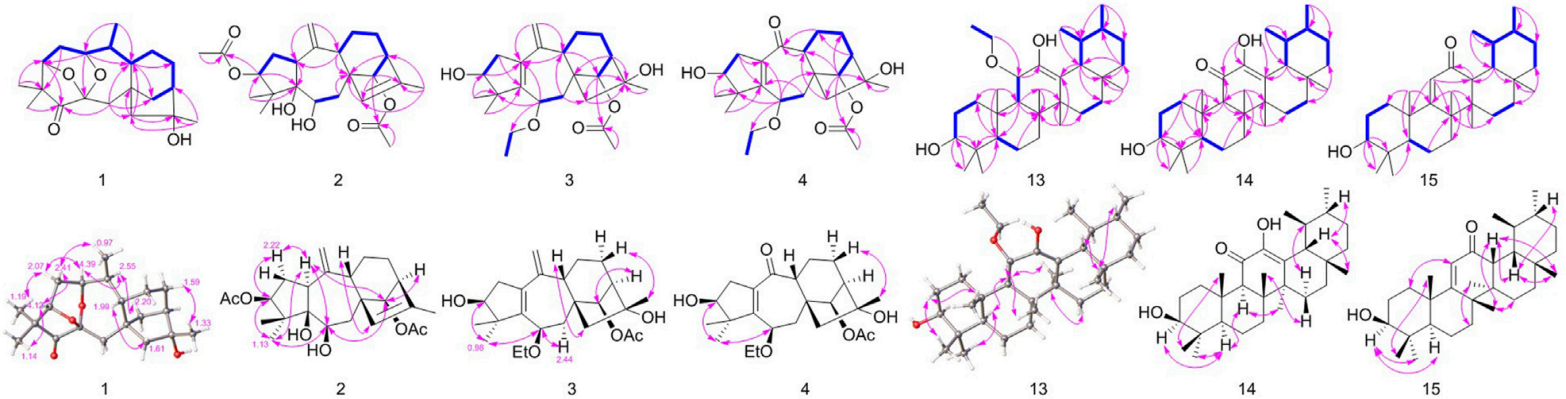
Key ^1^H−^1^H COSY (bold, blue), HMBC (single-headed), and key NOESY (double-headed) correlations.

More structural details were deduced from the analyses of 2D spectra (Figure 2). There was a spin system of H-3/H-2/H-1/H-10/H-20/H-9/H-11/H-12/H-13/H-14 in 1 according to the ^1^H−^1^H COSY spectrum. The correlations from H_3_-18/19 to C-3, C-4, and C-5; from H-3 to C-5; from H_2_-7 to C-5, C-9, C-14, and C-15; from H-20 to C-1 and C-9; from H_2_-15 to C-8, C-9, and C-14; from H_3_-17 to C-13, C-15, and C-16; and from H-1 to C-3, C-6, and C-9 were present in the HMBC spectrum. This 2D-NMR analysis (Figure 2) revealed that 1 was almost the same as pierisjaponin G except with the absence of an exocyclic double bond and the presence of a methyl doublet (*δ*_H_ 0.96, *δ*_C_ 17.7) with position 10 (*δ*_H_ 2.55; *δ*_C_ 38.7). In addition, the ring cleavage at C-1/C-5 with the oxygenated bridge formation at both C-3/C-6 and C-1/C-6 was found in this compound, which was confirmed by the residual unsaturation, molecular formula (required four oxygens), and chemical shifts of C-1 (*δ*C 73.3)/C-3 (*δ*C 79.7)/C-6(*δ*C 100.7), as well as the HMBC correlations mentioned in Figure 2. Thus, 1 was determined as shown. The configuration of 1 was confirmed according to NOESY analysis. H_3_-19 was randomly assigned as *α*-oriented and H_3_-18 as *β*-oriented. In the NOESY spectrum of 1 (Figure 2), H-3 was correlated to H_3_-18 and H-2*β*, H-2*β* showed a correlation to H_3_-20, H-2*α* was correlated to H_3_-19 and H-1, and H-1 was correlated to H-10, indicating that H_3_-18, H-3, and H_3_-20 were *β*-oriented, while H_3_-19, H-1, and H-10 were *α*-oriented. Cross-peaks of H-10*α*/H-14, H-9/H-15, and H_3_-17/H-12*β* revealed the *β*-orientation of H-9, H-15, and H_3_-17, which is consistent with the configuration of 1,5-*seco*-grayanane diterpenoid, such as pierisjaponin A (Zheng et al., 2020), reported from *Rhododendron*. Furthermore, after several attempts, the crystal of compound 1 was obtained from a methanol–water (87:13) solvent system by slow capillary evaporation at 4°C, which met the test quality requirements. Finally, X-ray diffraction analysis using Cu-K*α* radiation confirmed the elucidated structure and determined the absolute configuration of 1 to be 1*S*, 3*S*, 6*S*, 8*S*, 9*S*, 10*S*, 13*R*, 16*R* (Figure 4). Therefore, compound 1 was a novel grayanane diterpene with a remarkable 5/6/7/6/5 pentacyclic ring system.

Then, 3-AcO-grayanotoxin IX (2) was isolated as a white amorphous powder, and its molecular formula was established as C_24_H_34_O_6_ according to the HR-MS ion peak at *m/z* 419.2420 [M + H]^+^ (calcd for C_24_H_35_O_6_, 419.2428), which required eight degrees of unsaturation. The ^1^H and HSQC spectrum showed signs of one endocyclic (*δ*_C_ 134.9; *δ*_H_ 5.25) and one exocyclic double bond (*δ*_C_ 114.7; *δ*_H_ 4.93/5.05) and three oxymethines (*δ*_C_ 83.1, *δ*_H_ 4.85; *δ*_C_ 70.9, *δ*_H_ 3.66; and *δ*_C_ 82.4, *δ*_H_ 5.51). These characteristic signals indicated that 2 is a grayanane diterpenoid and was similar to grayanotoxin IX (Hikino et al., 1971); the difference was an acetyl group (*δ*_H_ 2.06, *δ*_C_ 21.1, and 172.9) at C-3 in 2, instead of a hydroxy group in grayanotoxin IX, suggesting that 2 is a 3-acetylization product of grayanotoxin IX. The H-6 in the grayanane diterpenes is usually *α*-oriented. No correlation between H-6 and H-15 was observed in the NOESY spectrum, which indicated that the bond connecting C-8 and C-15 was *β*-oriented. Through the NOESY analysis (Figure 2) and comparison with the CD curve of known compounds 7 and 10 (Figure SI), the absolute configuration was finally determined, and 2 was named 3-AcO-grayanotoxin IX.

**FIGURE 3:**
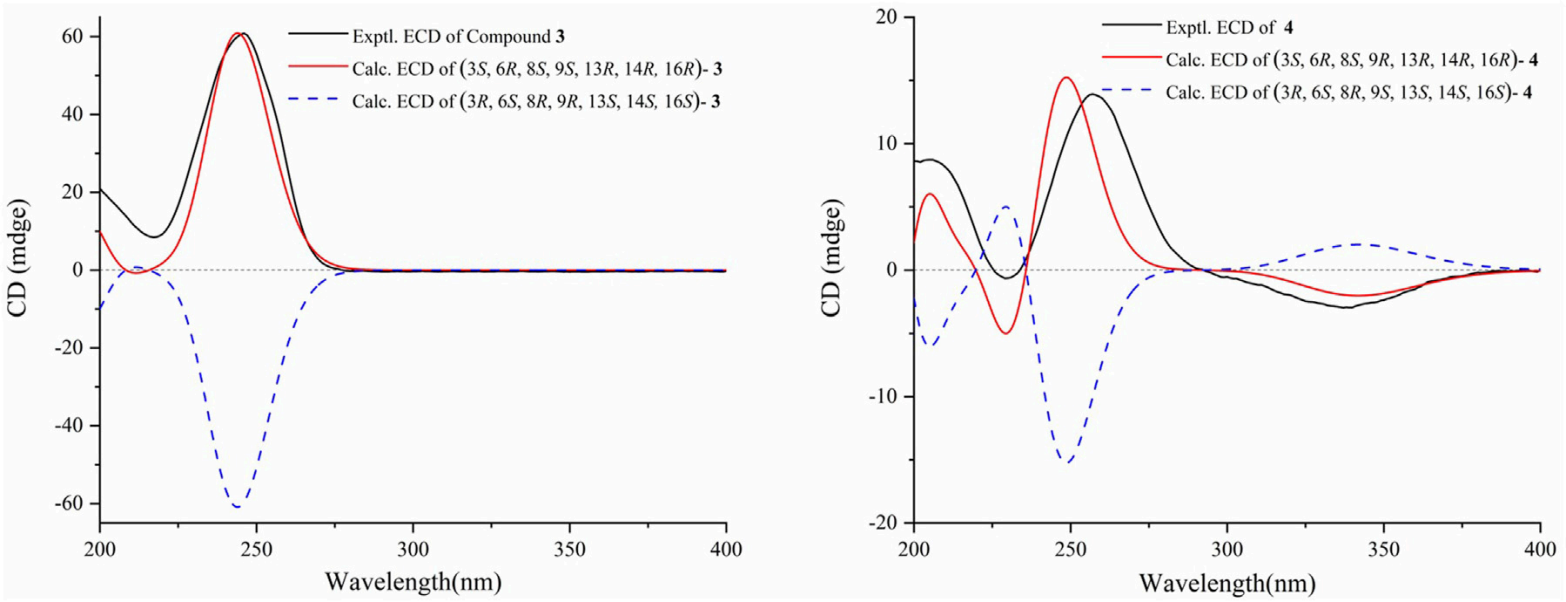
Experimental and calculated ECD spectra of compounds 3 and 4.

The molecular formula of 3*β*,16*α*-dihydroxy-6*β*-ethoxy-14*β*-acetoxy-grayan-1(5),10(20)-diene (3) was established as C_24_H_36_O_5_ by HRMS at *m/z* 405.2628 [M + H]^+^ (calcd for C_24_H_37_O_5_, 405.2636). The NMR data analysis of 3 showed some similarities to 3*β*,6*β*,16*α*-trihydroxy-14*β*-acetoxy-grayan-1(5),10(20)-diene, a grayanane diterpenoid from *Rhododendron micranthum* (Chai et al., 2020), except that the ethoxy (*δ*_C_ 66.2 and 16.1; *δ*_H_ 3.50/3.76 and 1.23) in 3 replaced the hydroxy at C-6, and similar to compound 2, the relative configuration was confirmed by NOESY analysis (Figure 2). Subsequently, the absolute configuration of 3 was determined to be 3*S*, 6*R*, 8*S*, 9*S*, 13*R*, 14*R*, 16*R* by comparing the calculated ECD curve with the experimental ECD curve (Figure 3). Therefore, 3 was named 3*β*,16*α*-dihydroxy-6*β*-ethoxy-14*β*-acetoxy-grayan-1(5),10(20)-diene. Note that compound 3 may serve as the acetylation product derived from the separation process.

3*β*,16*α*-dihydroxy-6*β*-ethoxy-14*β*-acetoxy-grayan-1(5)-ene-10-one (4), a white powder, was assigned the molecular formula of C_23_H_34_O_6_ by the [M + H]^+^ ion peak at *m/z* 407.2425 in the HRESIMS spectrum (calcd for C_23_H_35_O_6_, 407.2428). Similar to compound 3, the characteristic signals of H-3 (*δ*_H_ 3.85), H-6 (*δ*_H_ 4.18), and H-14 (*δ*_H_ 4.63) appeared in the ^1^H NMR spectrum of 4 (Table 1). Meanwhile, the 2D NMR spectra suggested the presence of an endocyclic double bond (*δ*_C_ 138.3/162.1), an ethoxycarbonyl (*δ*_C_ 171.1), an ethoxy (*δ*_H_ 3.54/3.76, 1.24; *δ*_C_ 65.1, 14.6), and a carbonyl group (*δ*_C_ 203.1) (Figure 2). The signals mentioned above indicated that the structure of 4 was similar to that of 3, which was classified as a grayanane diterpenoid. The differences in the structure were due to the degradation of C-20 and the carbonylation of C-10 in compound 4. The relative configuration of 4 was determined by the key NOESY correlations (Figure 2). Furthermore, the ECD calculation (Figure 3) was applied to confirm the absolute configuration of 4 to be 3*S*, 6*R*, 8*S*, 9*R*, 13*R*, 14*R*, 16*R*.

**FIGURE 4:**
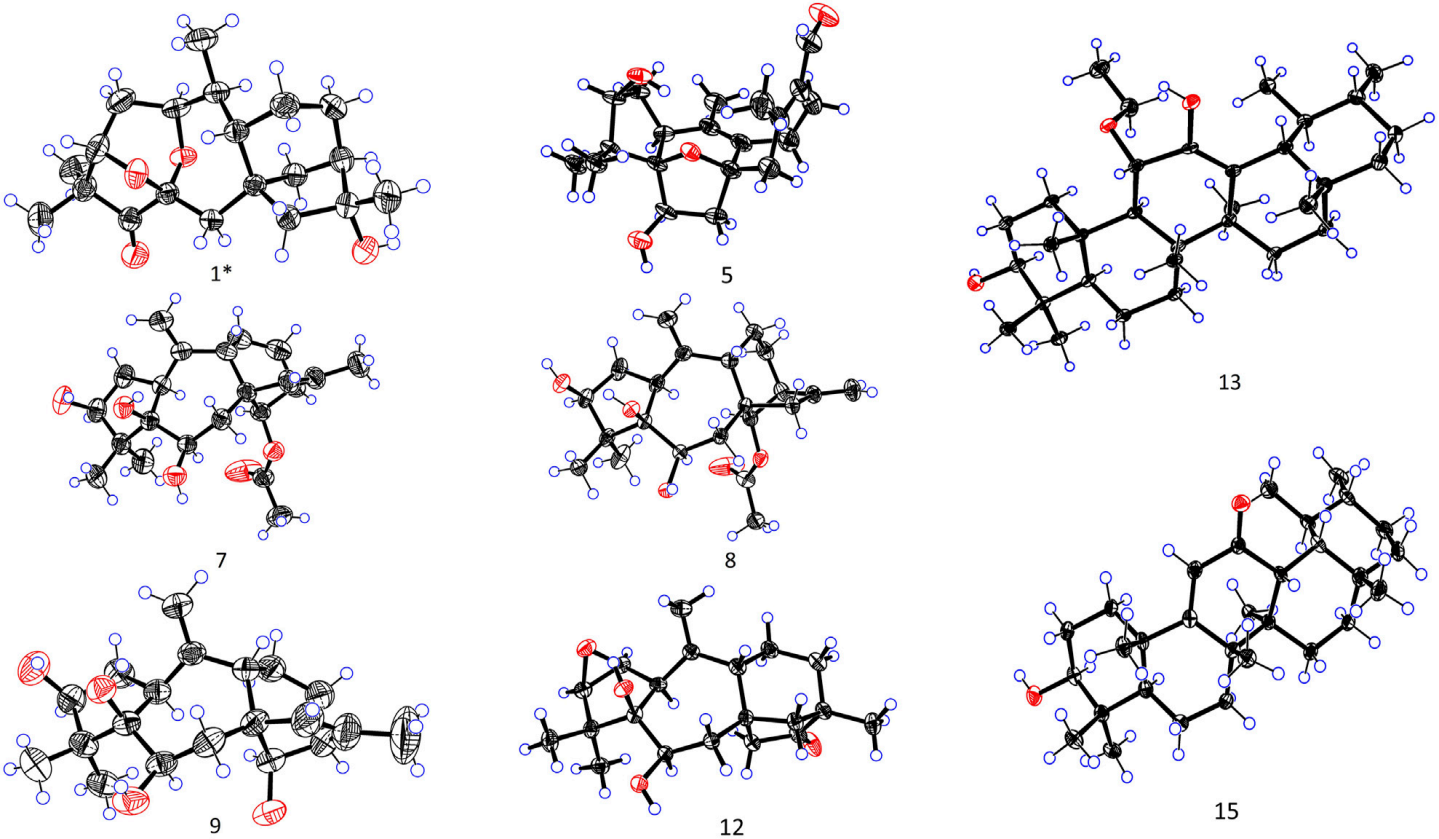
X-ray crystal structures of 1, 5, 7−9, 12−13, and 15.

Rhodauricanol A (5) was isolated as colorless crystals with the molecular formula of C_20_H_28_O_4_ (*m/z* 333.2060 [M + H]^+^, calcd for C_20_H_29_O_4_^+^, 333.2052) that was established by HR mass spectrometry and required seven degrees of unsaturation. ^1^H and ^13^C NMR data and detailed 2D-NMR analysis revealed that the structure of 5 was likely a diterpene. Compound 5 was subsequently identified as rhodauricanol A based on the comparison of NMR data reported in the literature (Feng et al., 2023) and single-crystal X-ray diffraction (Figure 4). Importantly, compound 5, with 5/6/5/7 tetracyclic skeleton diterpene, is now being reported for the second time.

Then, 11*α*-ethoxy-12-ene-3*β*,12-diol (13) was isolated as colorless crystals, and the molecular formula was determined to be C_32_H_54_O_3_ by the analysis of the HR-MS ion peak at *m/z* 509.3963 [M + Na]^+^ (calcd for C_32_H_54_NaO_3_, 509.3965) with the six degrees of unsaturation. The ^1^H-NMR data (Table 3) demonstrated the existence of eight methyls (0.80, s, H_3_-24; 0.80, s, H_3_-28; 0.93, d, H_3_-30; 0.94, d, H_3_-29; 0.99, s, H_3_-23; 1.08, s, H_3_-26; 1.11, s, H_3_-25; and 1.21, s, H_3_-27) and one ethoxy (*δ*_H_ 3.40/3.68 and 1.09). The ^13^C NMR data (Table 4) showed 32 carbon resonances, comprising one endocyclic double bond (*δ*_C_ 118.0, C-13; 142.4, C-12). These data indicated that 13 was likely an ursane-type triterpenoid, and detailed 2D-NMR analysis (Figure 2) revealed that the structure of 13 was similar to that of 11*α*-methoxyurs-12-ene-3*β*,12-diol (Kaweetripob et al., 2013), except that the methoxy group at C-11 was replaced by ethoxy in 13, which was also supported by the chemical shift of C-31 (*δ*_C_ 62.0) and C-32 (*δ*_C_ 15.9). Thus, the structure of 13 was determined as shown. Although the relative configuration of 13 was partially unclear in the NOESY spectrum, the absolute configuration (3*S*, 5*R*, 8*R*, 9*R*, 10*S*, 11*S*, 14*S*, 17*R*, 18*R*, 19*S*, 20*R*) was confirmed by single-crystal X-ray diffraction analysis (Figure 4).

Compound 14 was obtained as a white powder with the molecular formula of C_30_H_48_O_3_ obtained through the analysis of the [M + H]^+^ ion peak at *m/z* 457.3505 in the HRESIMS spectrum (calcd for C_30_H_49_O_3_, 457.3676), with seven degrees of unsaturation. The ^1^H NMR data (Table 3) of 14 displayed eight methyls, i.e., six singlets (*δ*_H_ 0.81, 0.83, 1.01, 1.15, 1.17, and 1.35) and two doublets (*δ*_H_ 0.79, d, *J* = 6.6 Hz; 0.92 d, *J* = 6.5 Hz), an oxygenated methine (*δ*_H_ 3.24), and a hydroxyl group (*δ*_H_ 6.28). The ^13^C NMR data (Table 4) of 14 revealed the presence of 30 carbons, including a carbonyl (*δ*_C_ 203.2) and a pair of conjugated endocyclic double bonds (*δ*_C_ 134.5 and 144.6). These characteristic data suggested that 14 was a ursane-type triterpenoid. Further 2D-NMR analysis revealed that the structure of 14 was similar to that of 3*β*,12,24-trihydroxyurs-12-en-11-one (Kaweetripob et al., 2013), and the difference was the absence of hydroxyl attached to C-24 (Figure 2). The relative stereochemistry of 14 was deduced by the NOESY experiment (Figure 2). Therefore, 14 was named as 3*β*,12-dihydroxyurs-12-en-11-one.

Then, 3*β*-hydroxy-12-oxours-11-ene (15) was isolated as a white powder with the molecular formula of C_30_H_48_O_2_ with seven degrees of unsaturation, deduced from the analysis of the molecular ion peak at *m/z* 441.3728 [M + H]^+^ (calcd. for C_30_H_49_O_2_^+^, 441.3727) in HR-MS. The ^1^H NMR data (Table 3) of 15 showed eight methyls, i.e., six singlets (*δ*_H_ 0.84, 0.99, 1.05, 1.09, 1.21, and 1.29) and two doublets (*δ*_H_ 0.69, d, *J* = 6.7 Hz; 0.87 d, *J* = 6.4 Hz), an oxygenated methine (*δ*_H_ 3.24), and an olefin proton (*δ*_H_ 5.92). The ^13^C NMR data (Table 4) of 15 revealed 30 carbons, including a carbonyl (*δ*_C_ 203.2) and a pair of conjugated endocyclic double bonds (*δ*_C_ 123.5 and 179.4). A detailed analysis of the NMR data manifested that the structure of compound 15 was similar to that of 3*β*-hydroxy-11-oxours-12-ene (Qiu et al., 2017; Jiang et al., 2012), and both were ursane-type triterpenoids. The obvious difference was the presence of the double bond of C-9/C-11 and the carbonyl of C-12 in 15, which was proved by the 2D-NMR correlations (Figure 2). However, it is difficult to entirely determine the relative configuration due to insufficient NOESY interactions. Subsequently, the X-ray diffraction analysis using Cu-K*α* radiation was applied to determine the absolute configuration to be 3*S*, 5*R*, 8*S*, 10*S*, 11*S*, 13*R*, 14*R*, 17*R*, 18*S*, 19*S*, 20*R*.

### 3.2 Antibacterial effect

The antibacterial activities of compounds 1–30 against *S. aureus* subsp*. aureus* (ATCC29213) and *Escherichia coli* (ATCC25922) were tested, and the MIC_50_ values were obtained by a standardized microdilution method according to CLSI Performance Standards for Antimicrobial Susceptibility Testing 2009 and reported method (Tang et al., 2019; Clinical Laboratory Standards Institute, 2009). The results showed that some of the compounds exhibited antibacterial activity against *S. aureus* with an MIC_50_ value of 1.4–24.3 *μ*g/mL. (Table 5).

**TABLE 5:** Inhibitory effects of compounds on *Staphylococcus aureus* and *Escherichia coli* (the results are expressed as MIC_50_ values in μg/mL).

Compounds	*S. aureus*	*E. coli*
14	10.87 ± 0.58	>80
15	1.50 ± 0.10	>80
18	1.46 ± 0.01	>80
20	3.29 ± 0.82	>80
27	24.31 ± 0.42	>80
28	1.76 ± 0.05	>80
30	17.38 ± 0.17	>80
Penicillin G-Na	0.58 ± 0.01	—
Ceftazidime	—	1.15 ± 0.01

## 4 Conclusion

In conclusion, rhodosimsiin A (1) bearing an unprecedented 1,5-*seco*-1,6 and 3,6-epoxy grayanane diterpene skeleton, representing a novel 5/6/7/6/5 pentacyclic ring system, together with three new grayanane diterpenes (2−4), three new triterpene (13−15), and known analogs (5−12, and 16−30), was isolated from the leaves of *R. simsii* Planch with an activity-guided method. Their structures were elucidated by comprehensive spectroscopic analyses, and 1, 5, 7−9, 12−13, and 15 were confirmed by X-ray crystallography. The discovery of 5/6/7/6/5 pentacyclic grayanane diterpene (rhodosimsiin A, 1) and norditerpene (3*β*,16*α*-dihydroxy-6*β*-ethoxy-14*β*-acetoxy-grayan-1(5)-ene-10-one, 4) expands the grayanane skeletons and provided a new dimension to the diversity of the diterpene family. Additionally, rhodauricanol A (5), with 5/6/5/7 tetracyclic skeleton diterpene, is now being reported for the second time. Compounds 14, 15, 18, 20, 27, 28, and 30 exhibited potent antibacterial activity with an MIC_50_ value of 1.4–24.3 *μ*g/mL against *S. aureus*.

These discoveries could potentially stimulate further research in synthetic and pharmaceutical fields regarding the chemical and pharmacological properties of *Rhododendron*.

## Data Availability

The datasets presented in this study can be found in online repositories. The names of the repository/repositories and accession number(s) can be found in the article/Supplementary Material. Further inquiries can be directed to the corresponding authors.
