# Supplementary material for: Identification of antibacterial constituents from Rhododendron simsii Planch with an activity-guided method
Source: Front Pharmacol. 2024 Oct 8;15:1490335. doi: 10.3389/fphar.2024.1490335 (PMC11493700; doi:10.3389/fphar.2024.1490335)
Supplement: Supplementary file 2 [file DataSheet1.docx]

Identification of antibacterial constituents from *Rhododendron simsii* Planch with activity-guided method

Yongji Lai, ^1,†^ Yu-ting Zhong,^3,†^ Yu Liang,^1^ Wei-chen Chen,^3^ Qiuyan Liao,^3^ Mu Li,^4^ Pan Han,^1^ You-sheng Cai,^3^* Fuqian Wang ^2,^*

^1.^ Department of Pharmacy, The Central Hospital of Wuhan, Wuhan 430014, China

^2.^ Department of Pharmacy, Wuhan No.1 Hospital, Wuhan 430022, China

^3.^ Key Laboratory of Combinatorial Biosynthesis and Drug Discovery, Ministry of Education and School of Pharmaceutical Sciences, Wuhan University, Wuhan, 430071, China

^4.^ Department of Medicament, College of Medicine, Tibet University, Lhasa, China

^†^ These authors contributed equally to this work.

**CONTENTS**

HR-MS, 1D and 2D NMR spectra of compound **1** 3-6

HR-MS, 1D and 2D NMR spectra of compound **2** 7-10

HR-MS, 1D and 2D NMR spectra of compound **3** 11-14

HR-MS, 1D and 2D NMR spectra of compound **4** 15-18

HR-MS, 1D and 2D NMR spectra of compound **13** 19-22

HR-MS, 1D and 2D NMR spectra of compound **14** 23-26

HR-MS, 1D and 2D NMR spectra of compound **15** 27-30

Experimental ECD spectra of compounds **2**, **7** and **10** 31

Relative inhibition curves of compounds and positive control 31

Relative inhibition rate of subfractions against *Staphylococcus aureus* 32

X-ray crystallographic data 32

# HR-MS of compound 1

# CD spectrum of compound 1


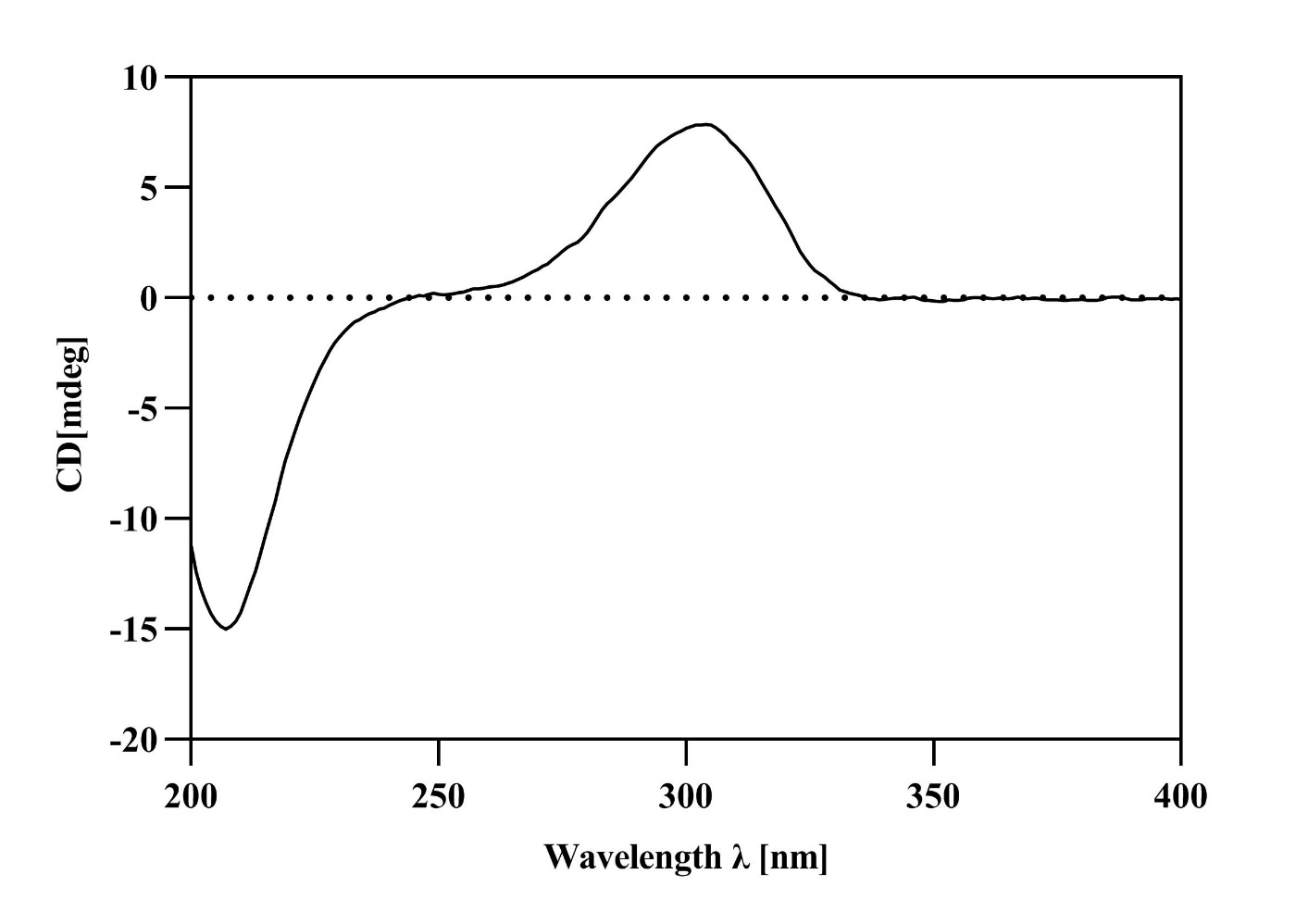


# ^1^H NMR of compound 1


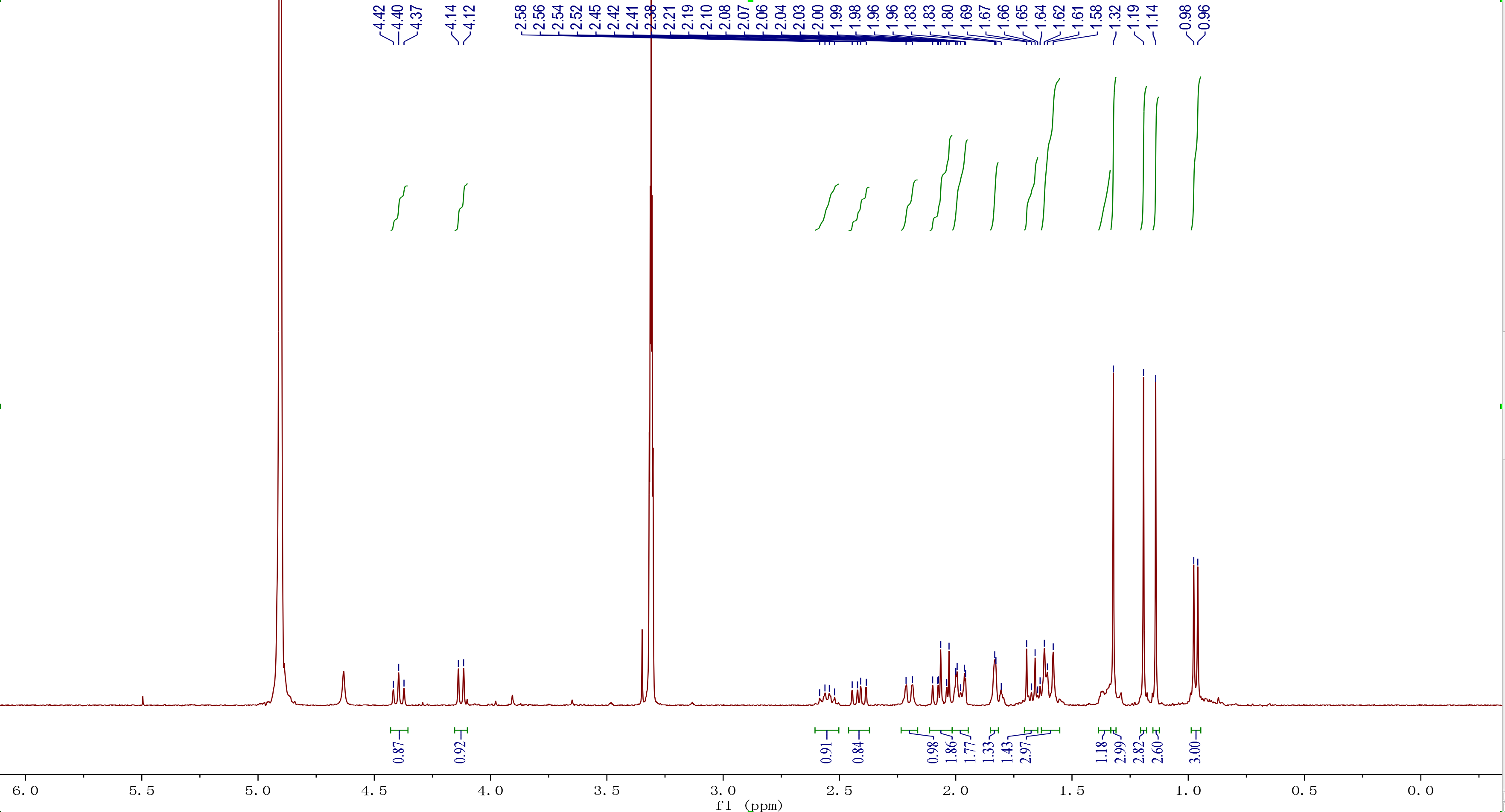


# ^13^C NMR of compound 1


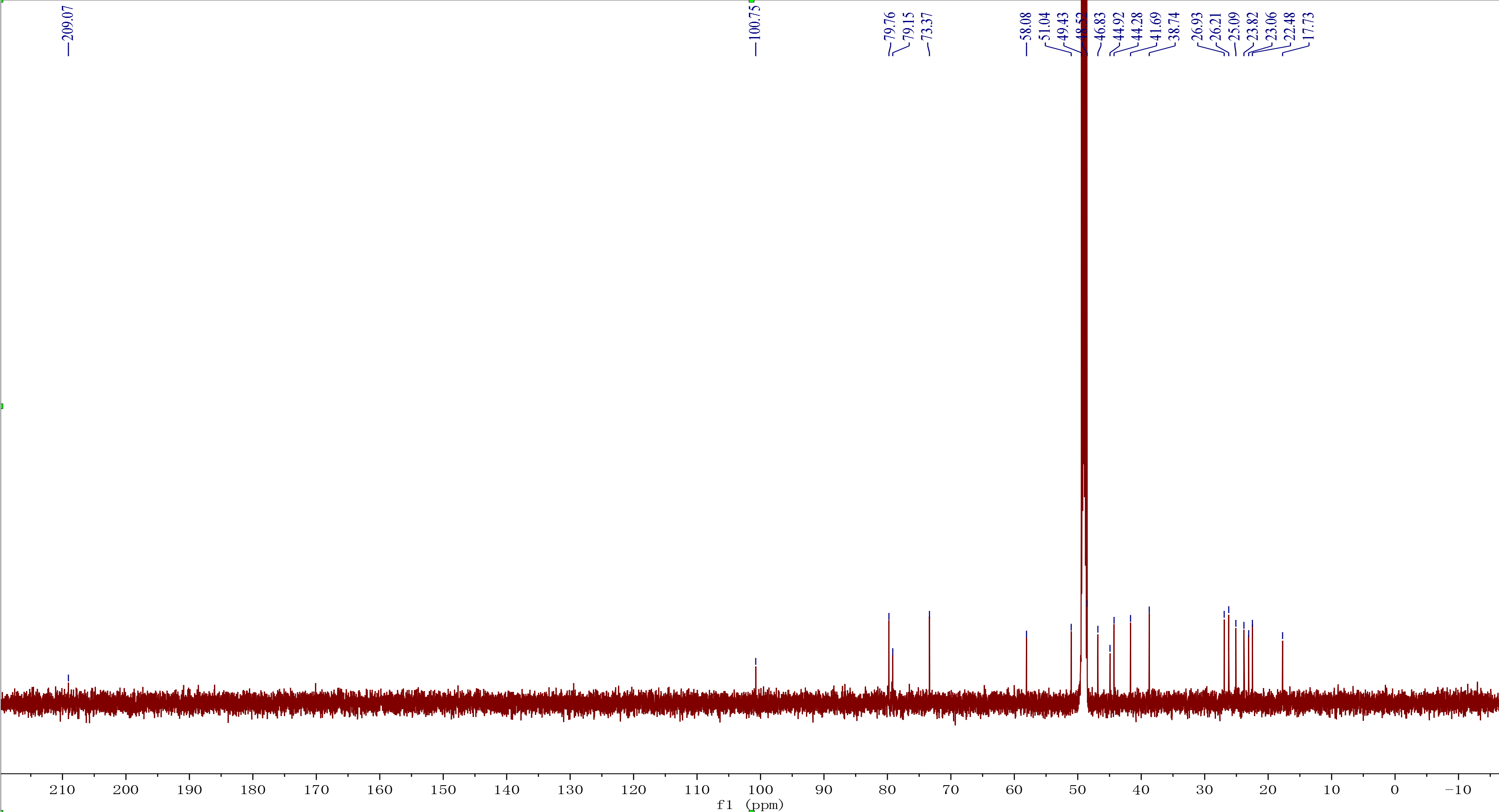


# HSQC of compound 1


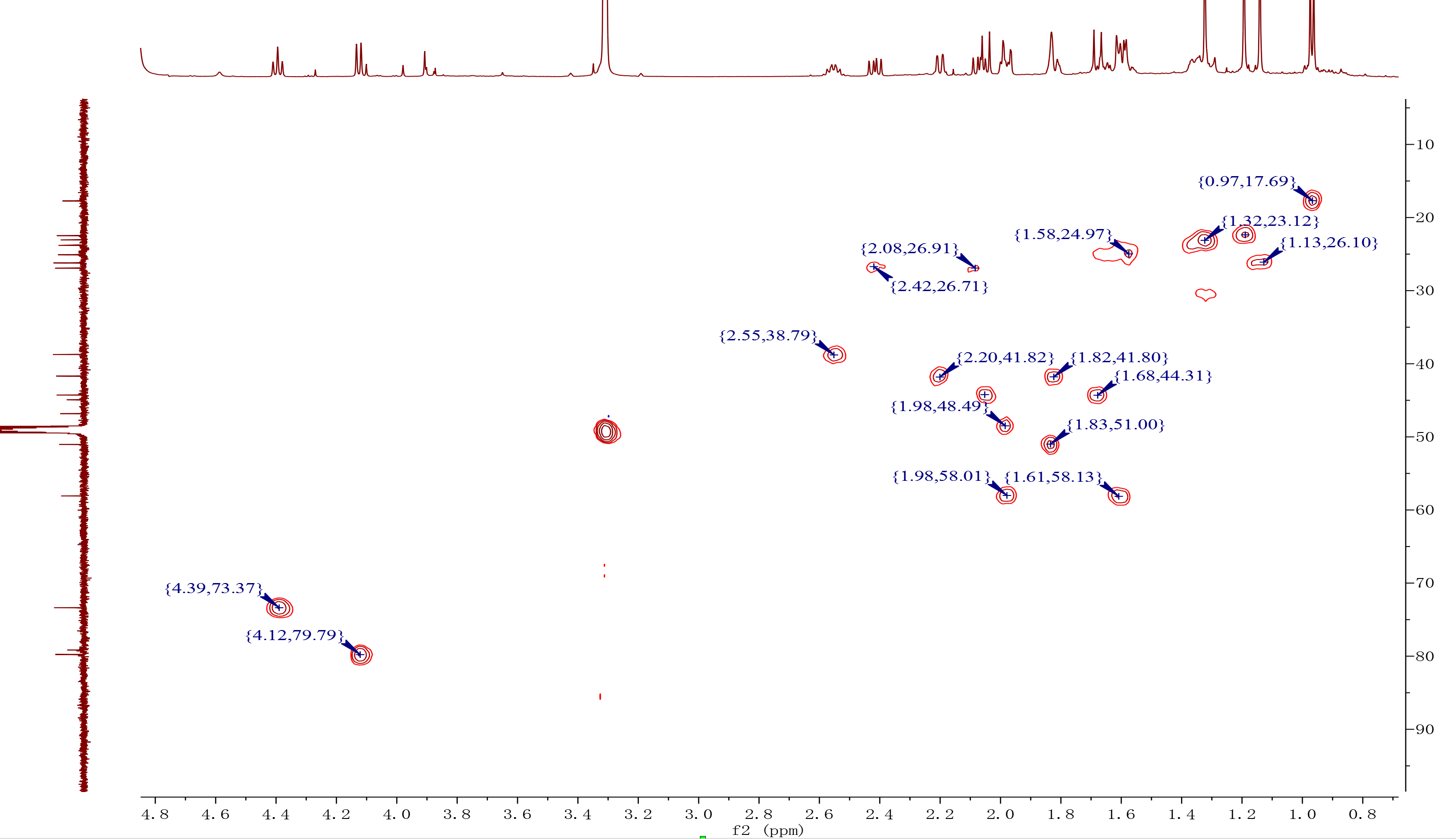


# HMBC of compound 1


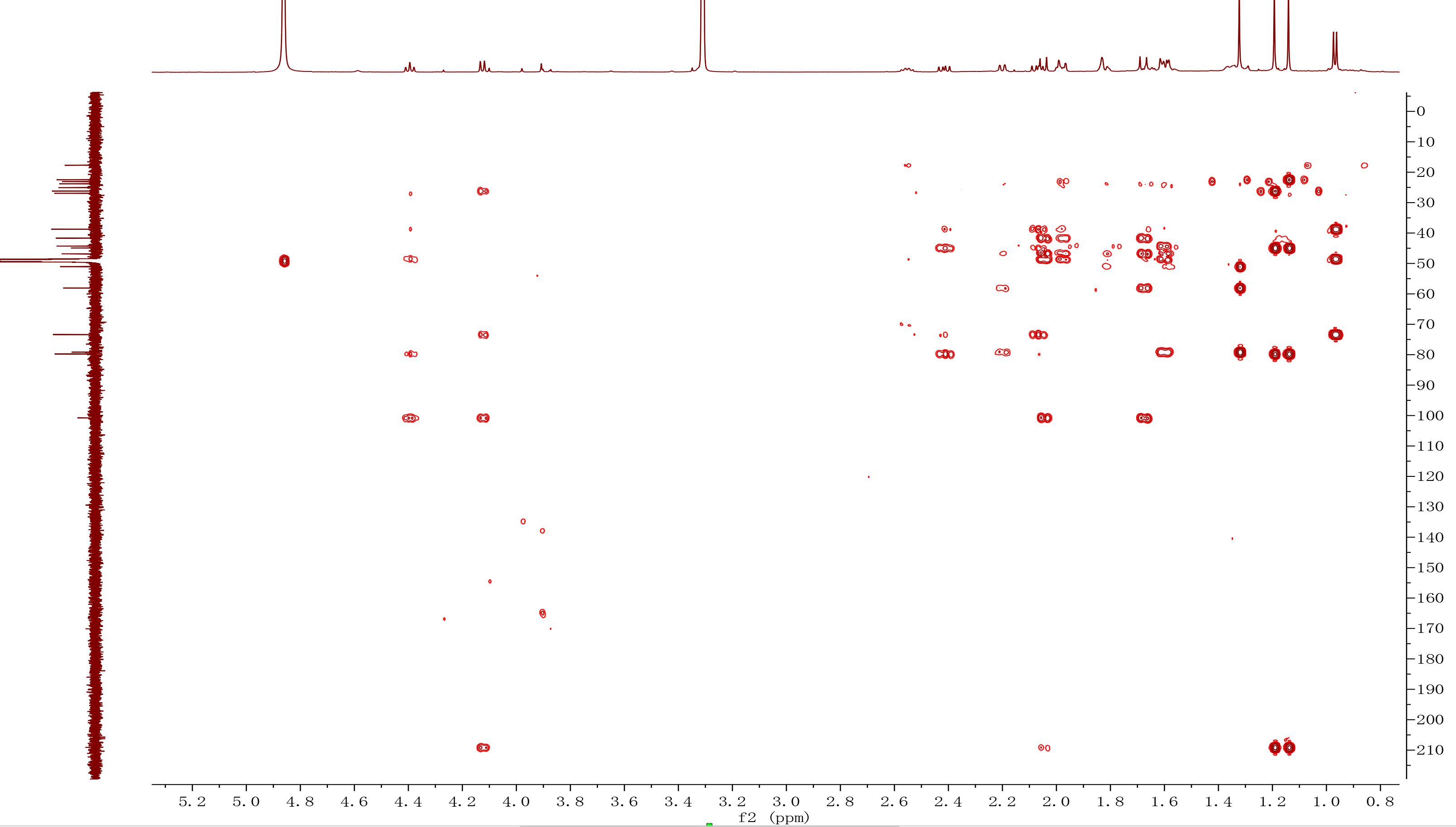


# ^1^H−^1^H COSY of compound 1


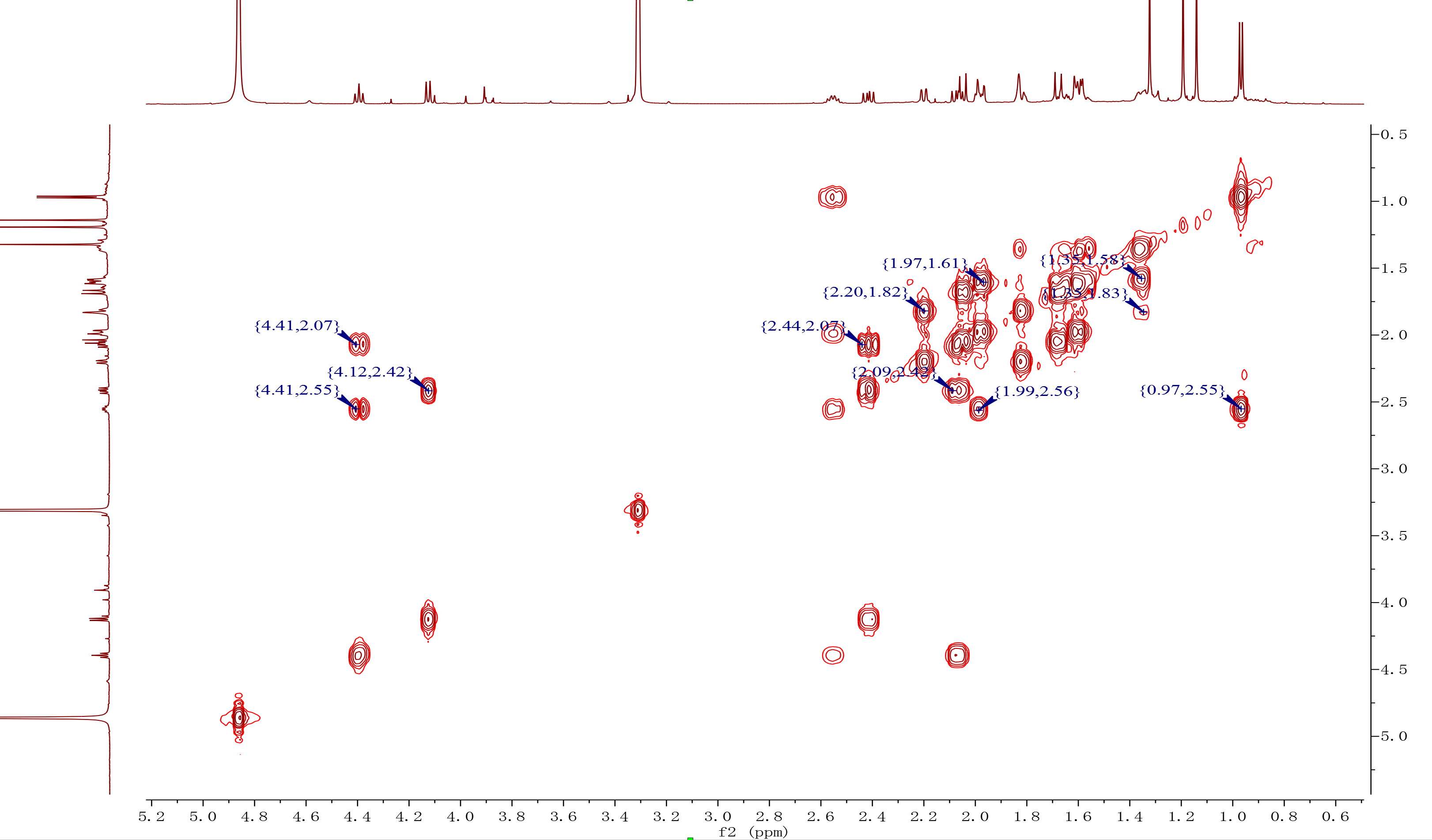


# NOESY of compound 1


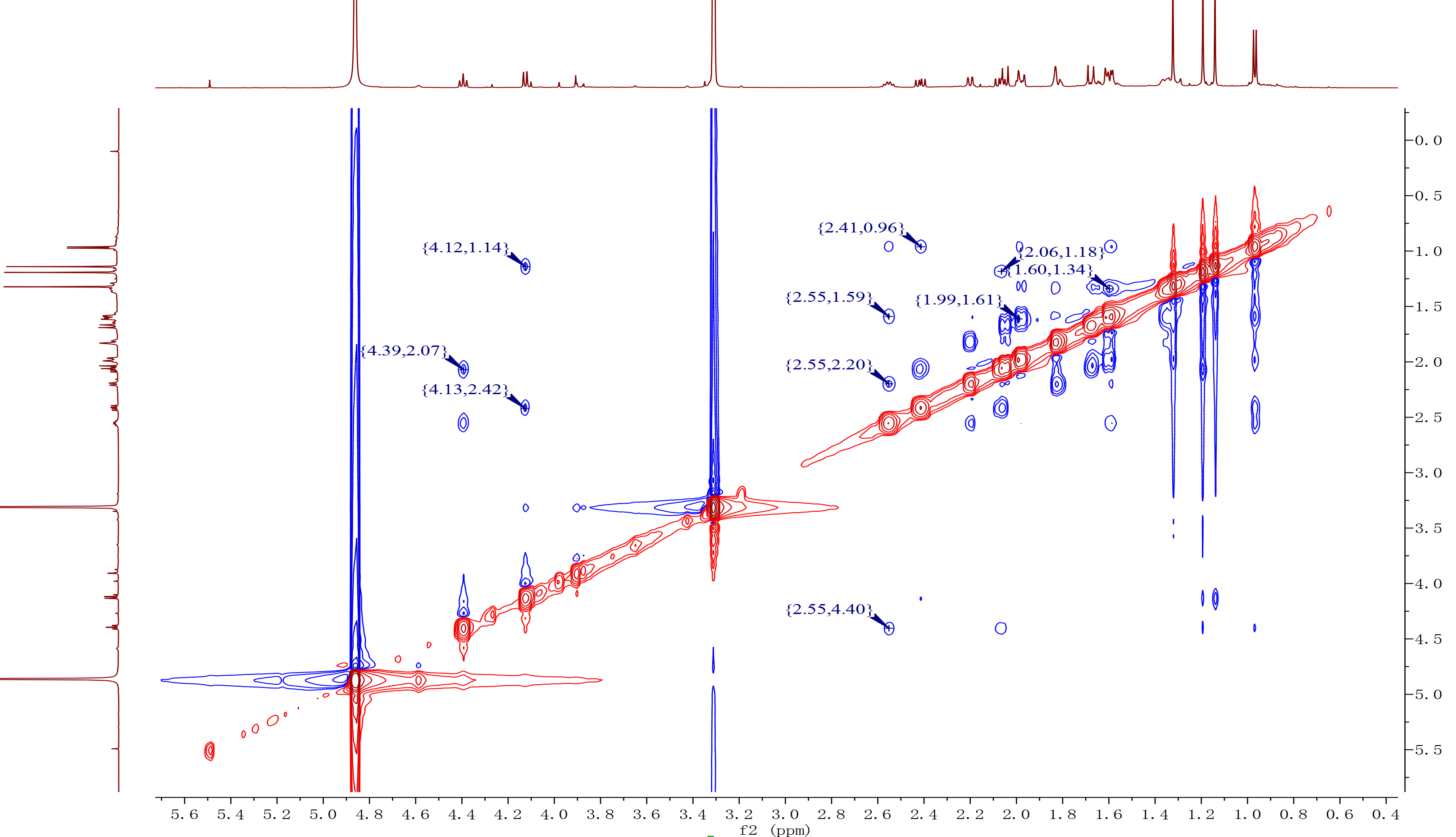


# HR-MS of compound 2

# CD spectrum of compound 2


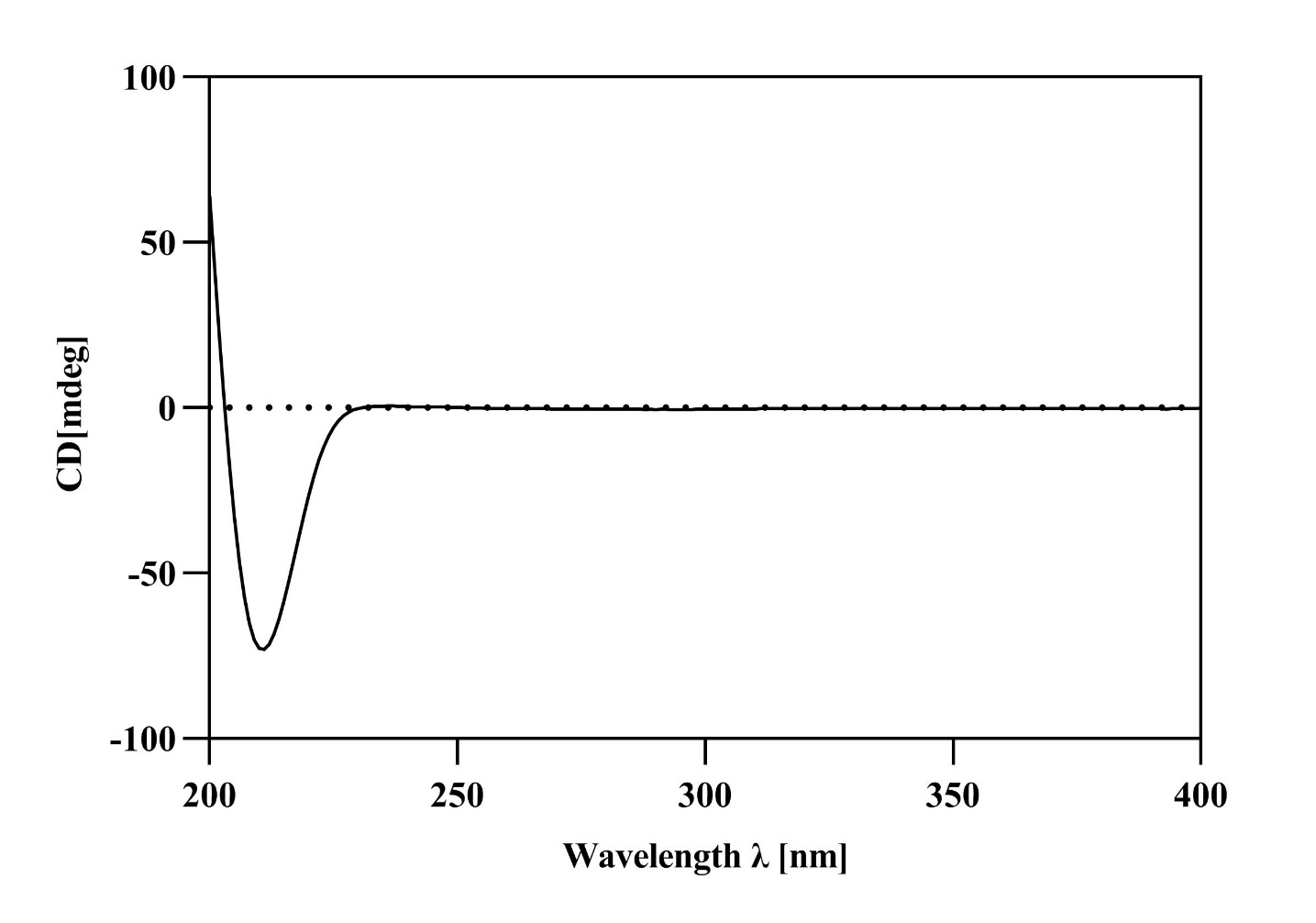


# ^1^H NMR of compound 2


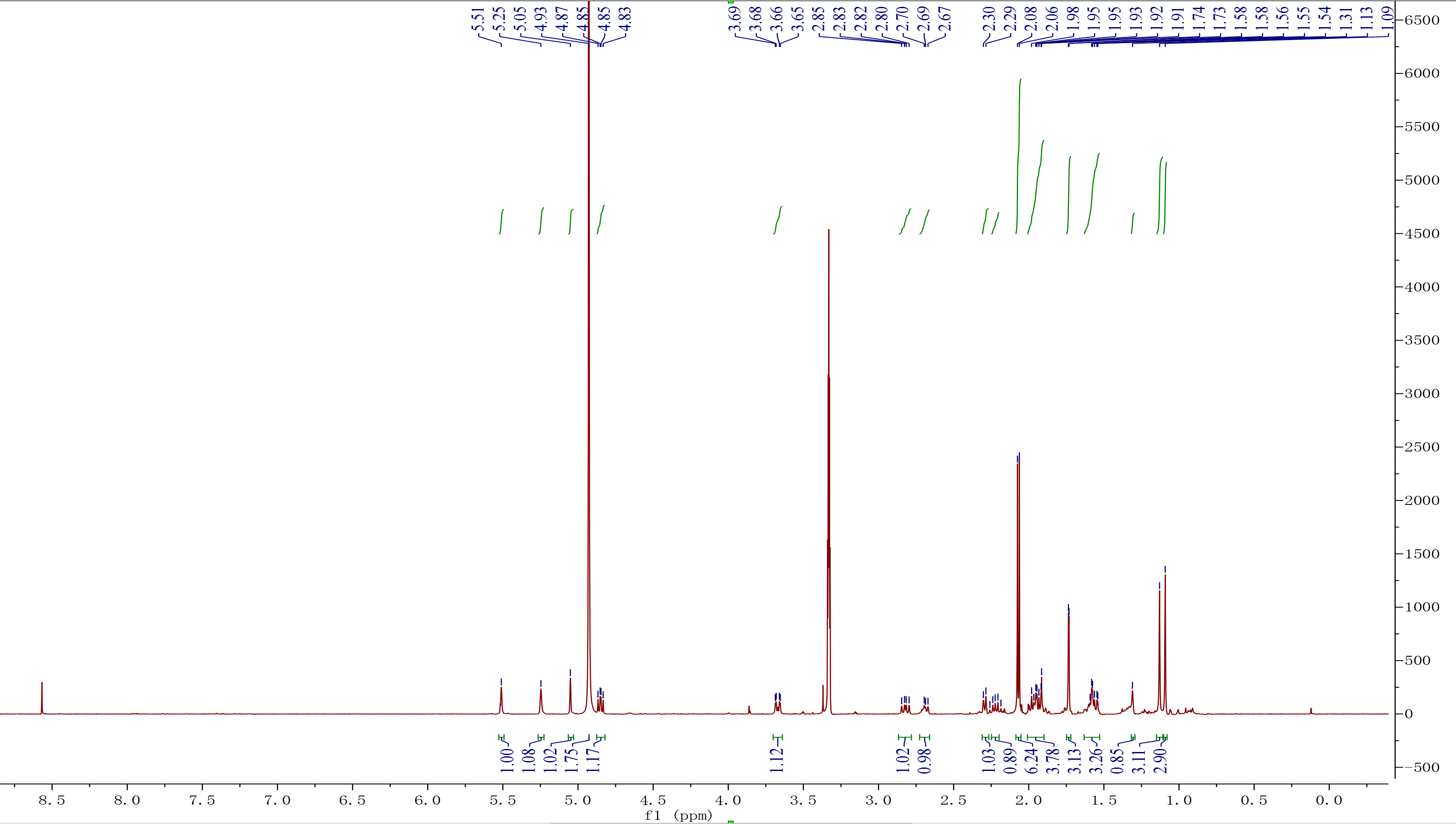


# ^13^C NMR of compound 2


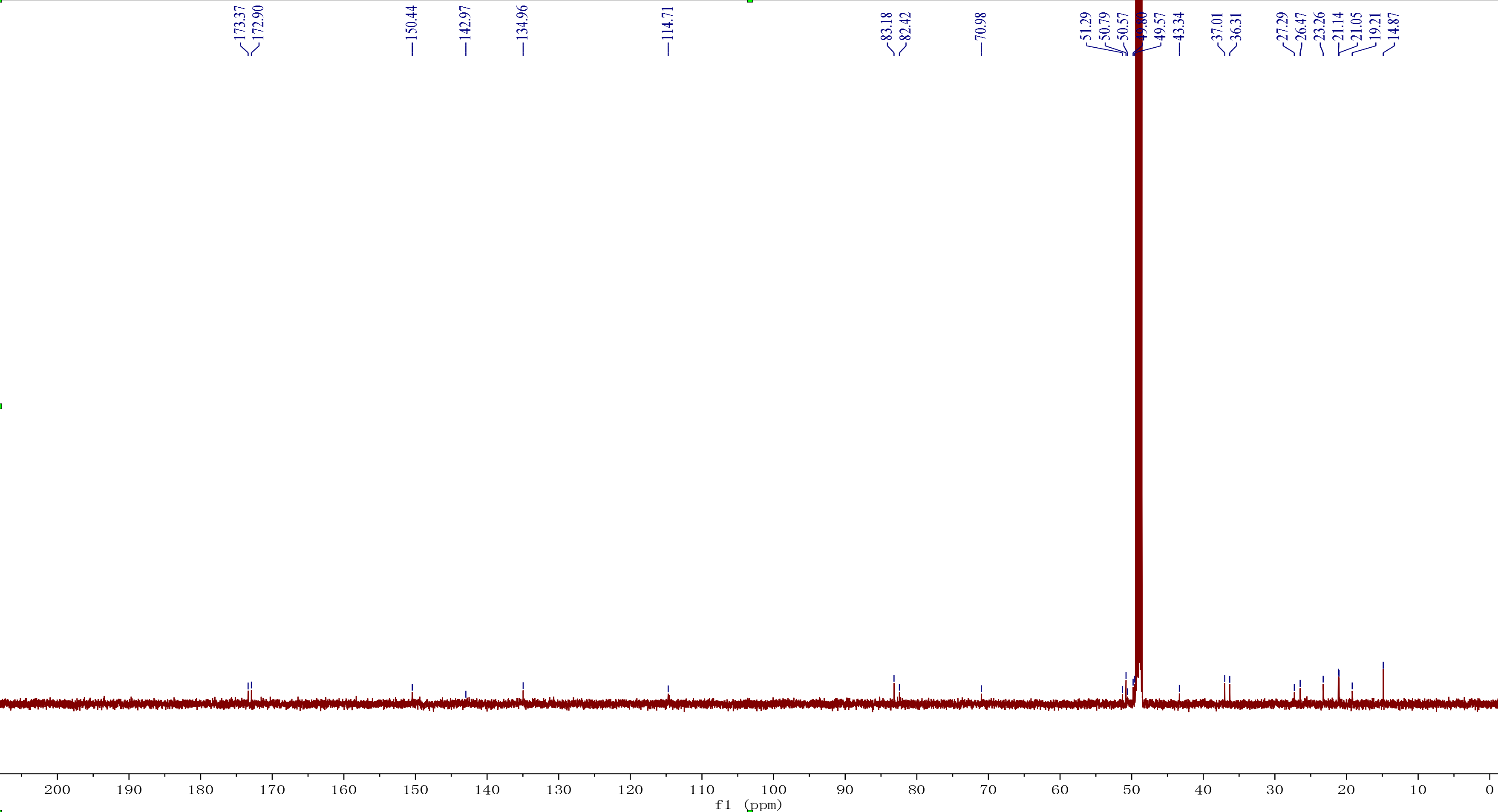


# HSQC of compound 2


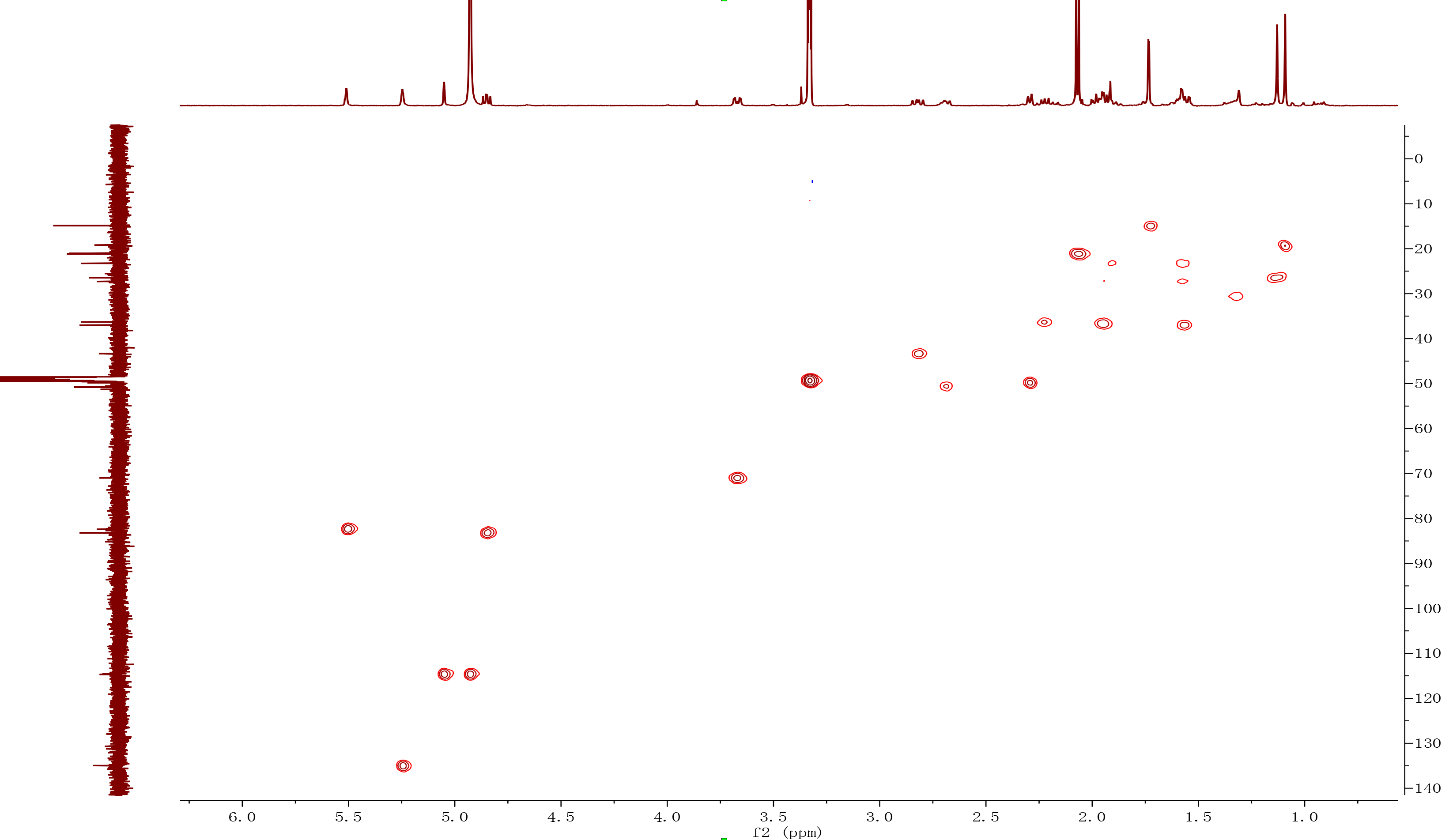


# HMBC of compound 2


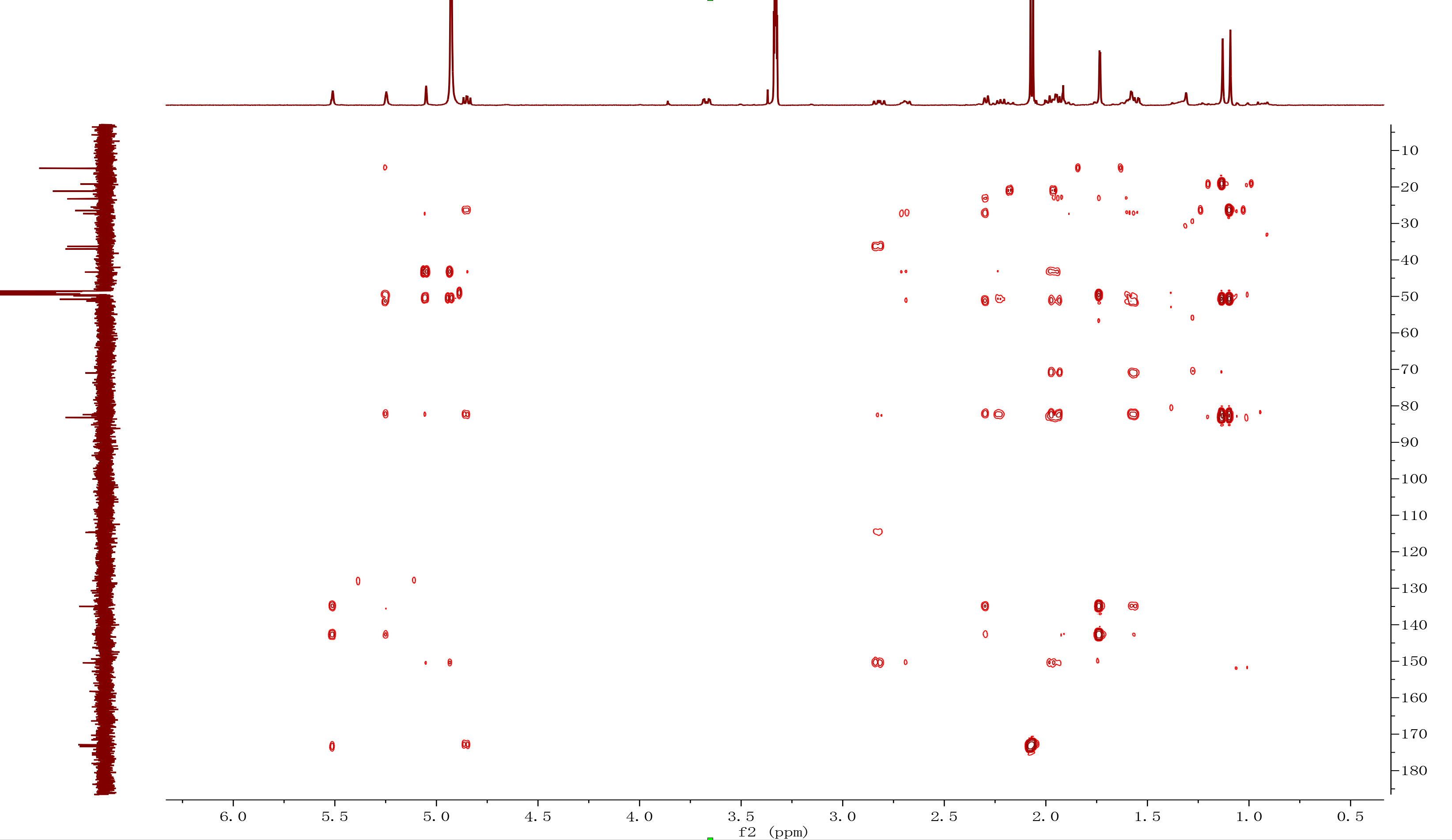


# ^1^H−^1^H COSY of compound 2


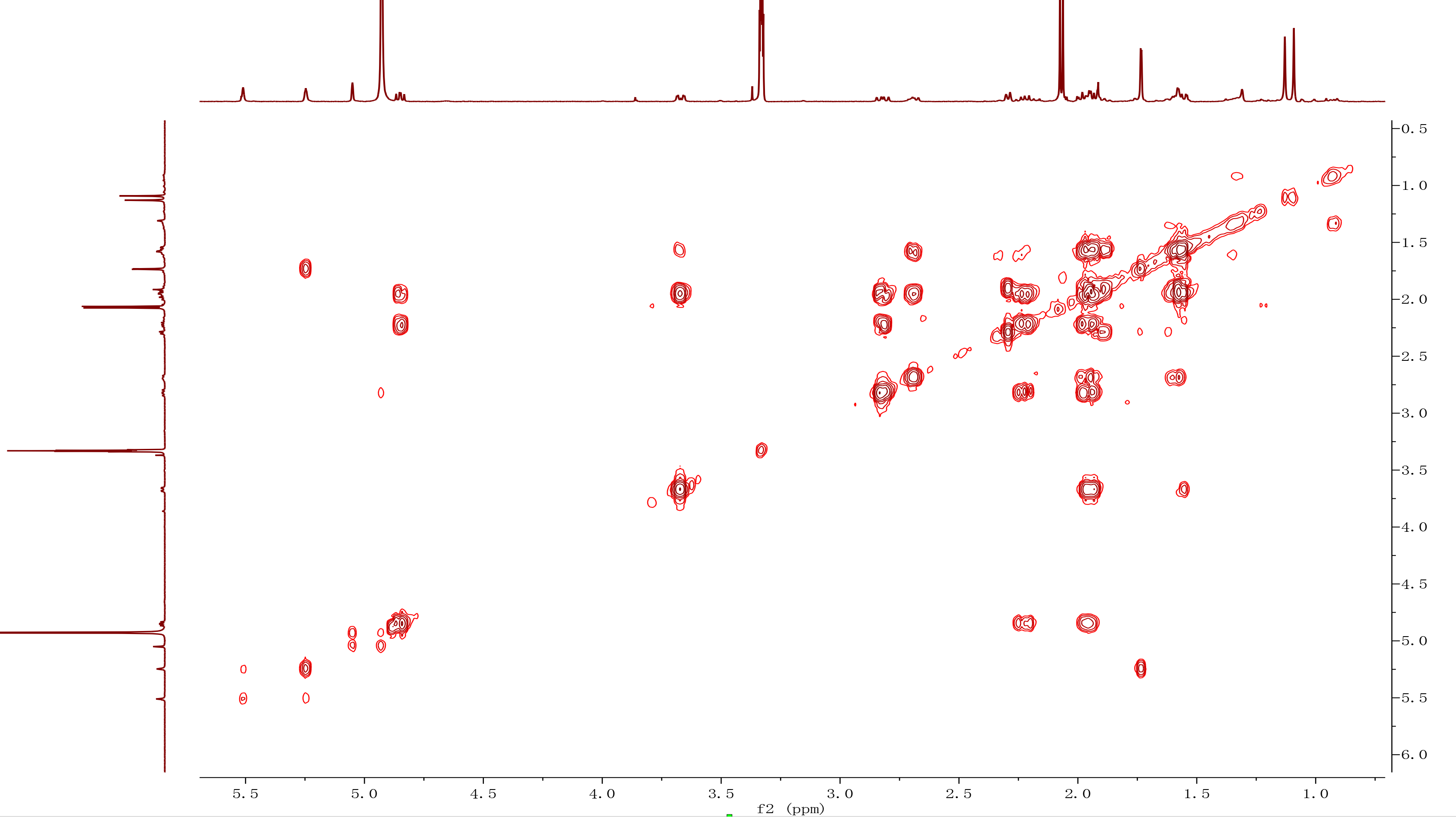


# NOESY of compound 2


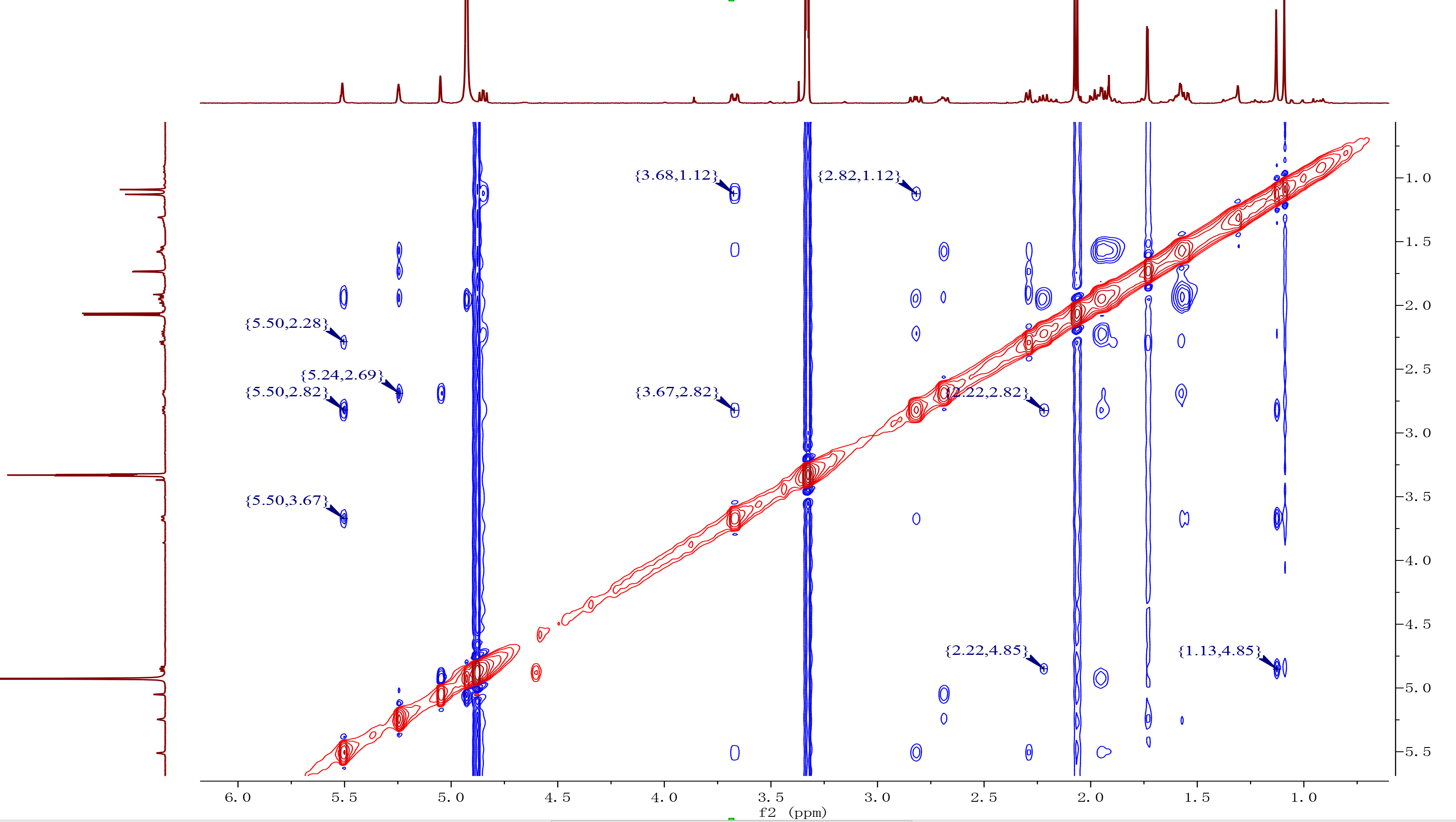


# HR-MS of compound 3

# CD spectrum of compound 3


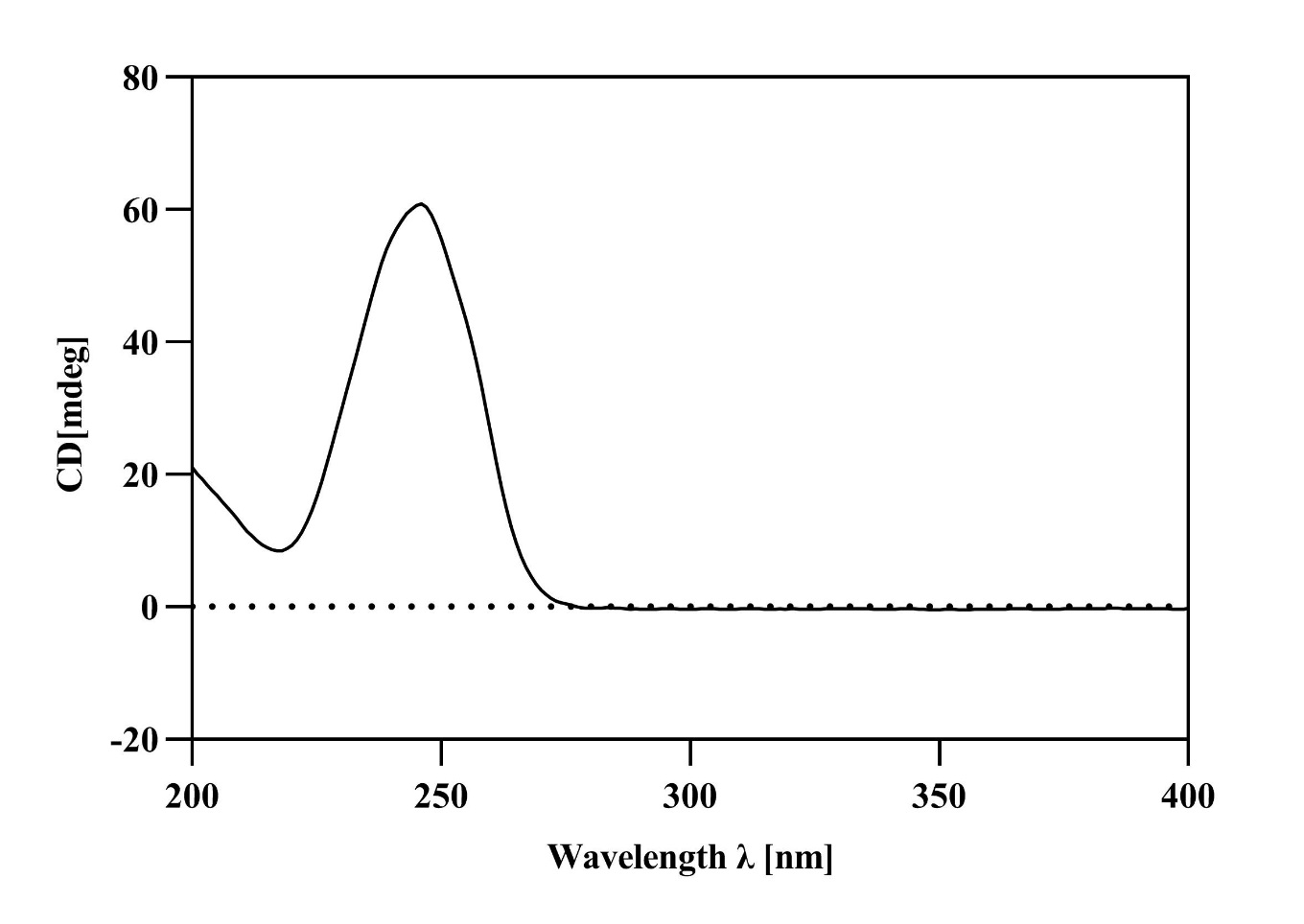


# ^1^H NMR of compound 3


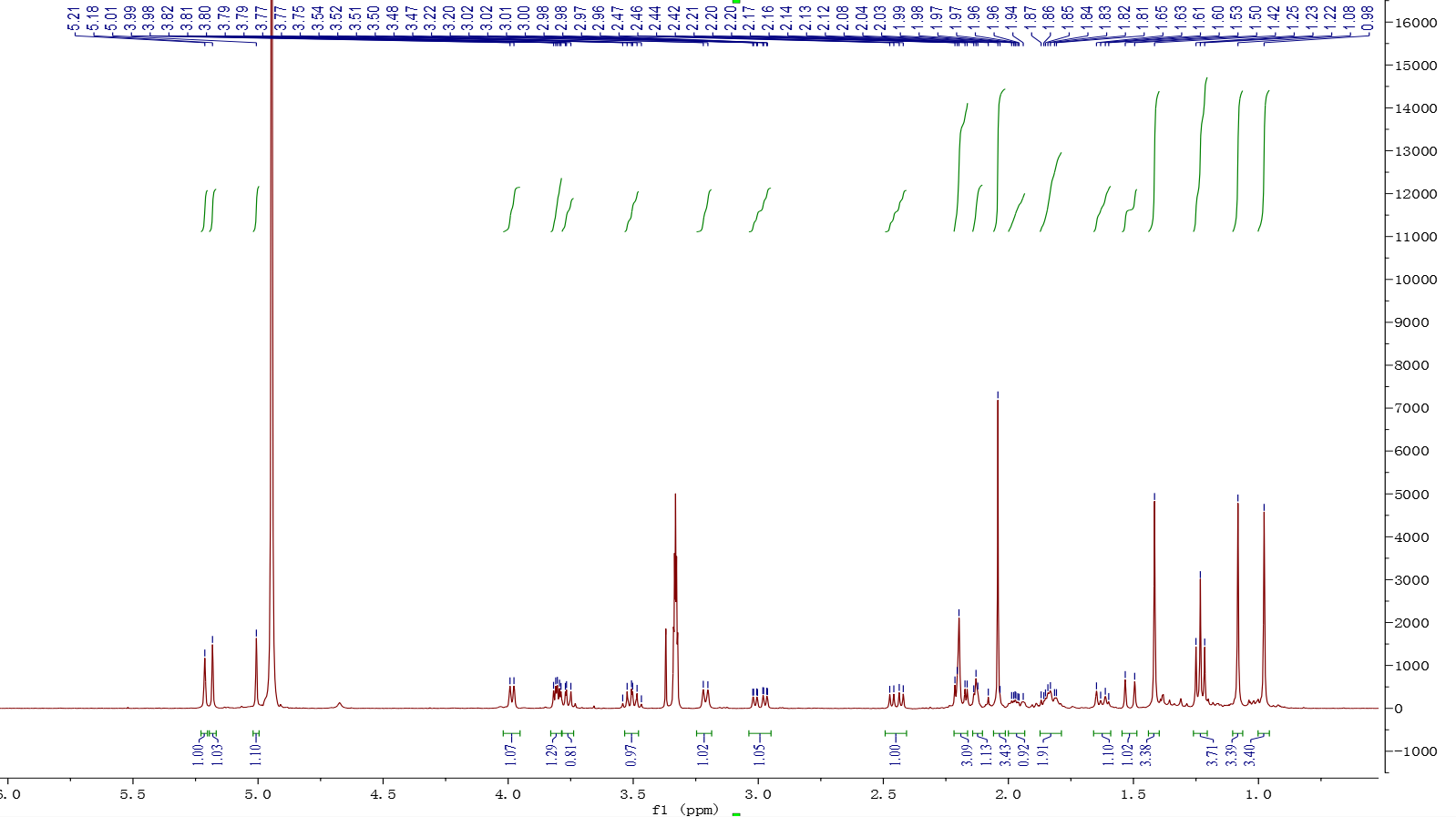


# ^13^C NMR of compound 3


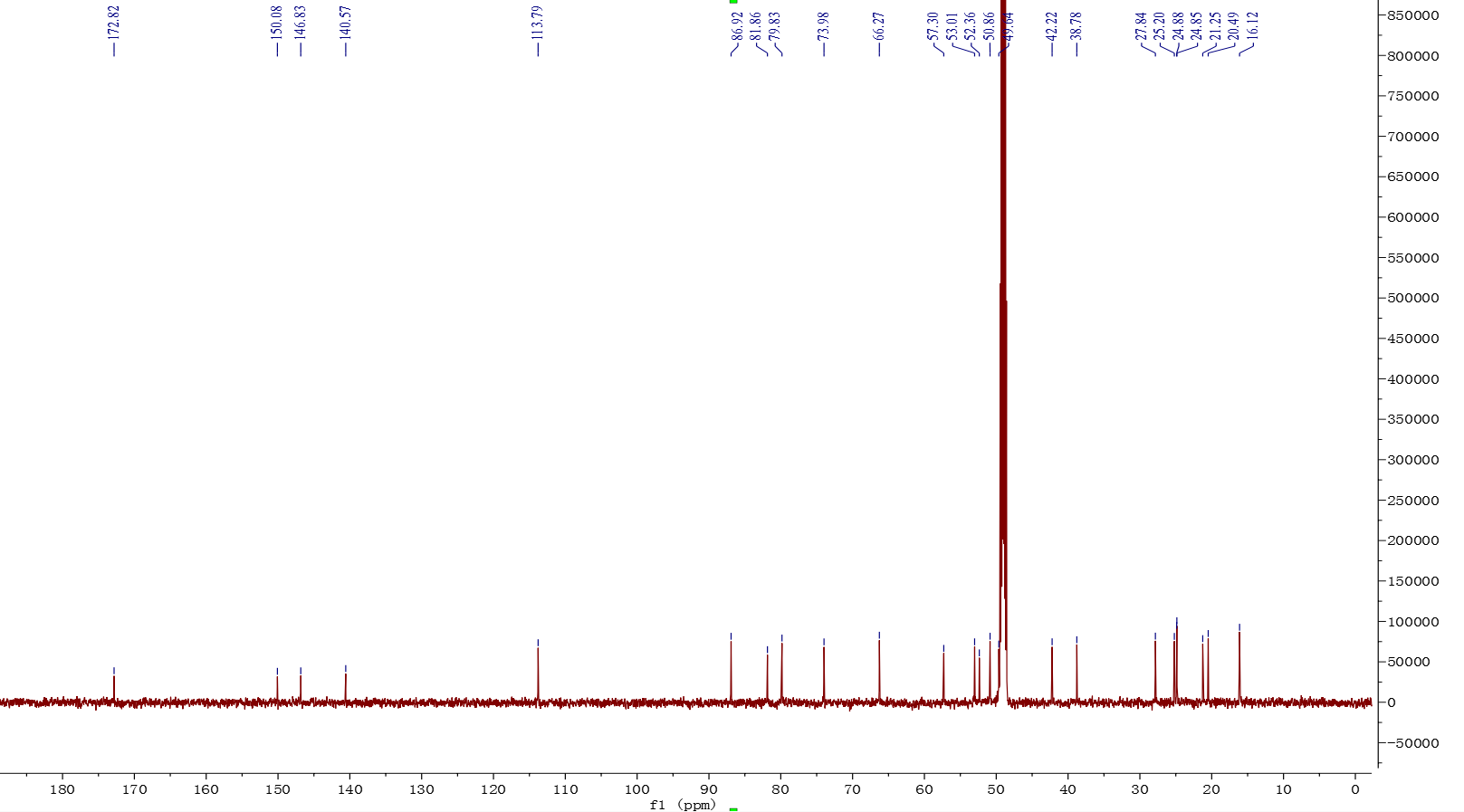


# HSQC of compound 3


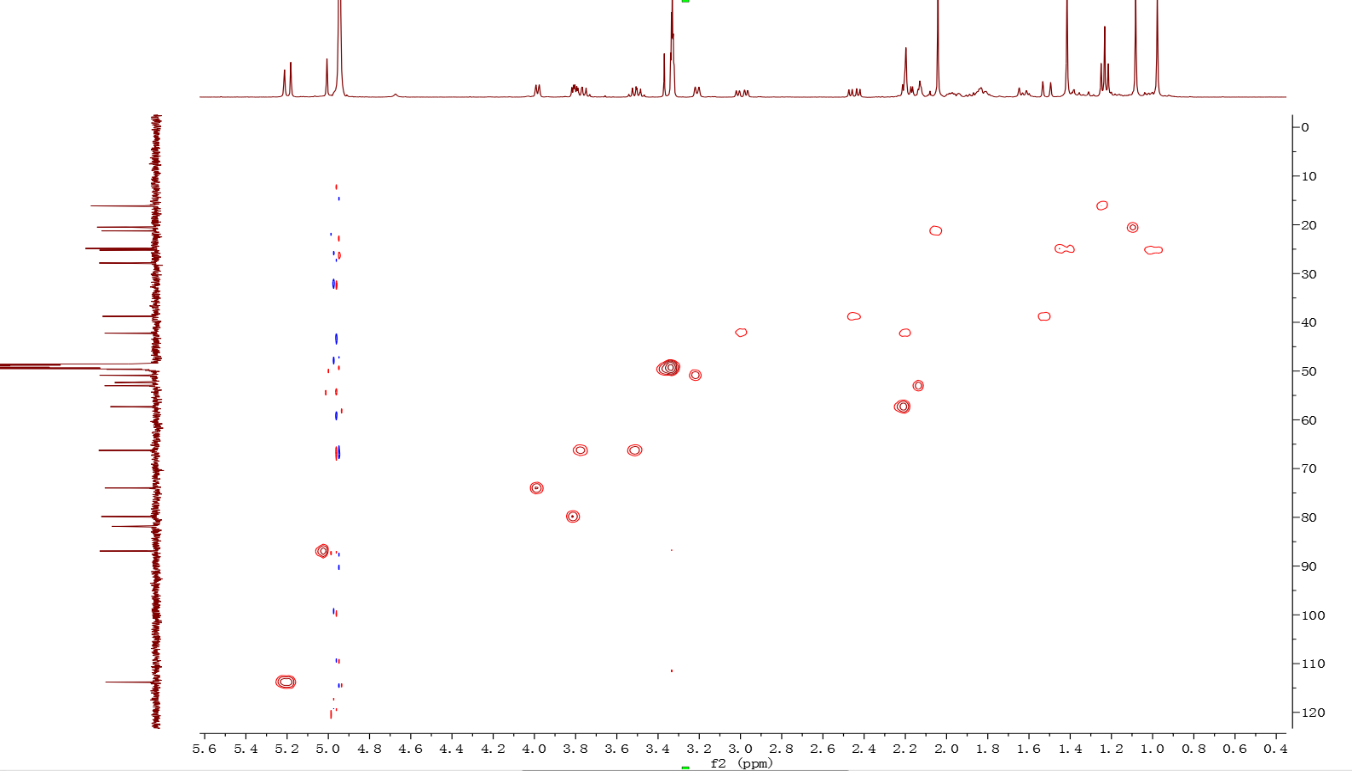


# HMBC of compound 3


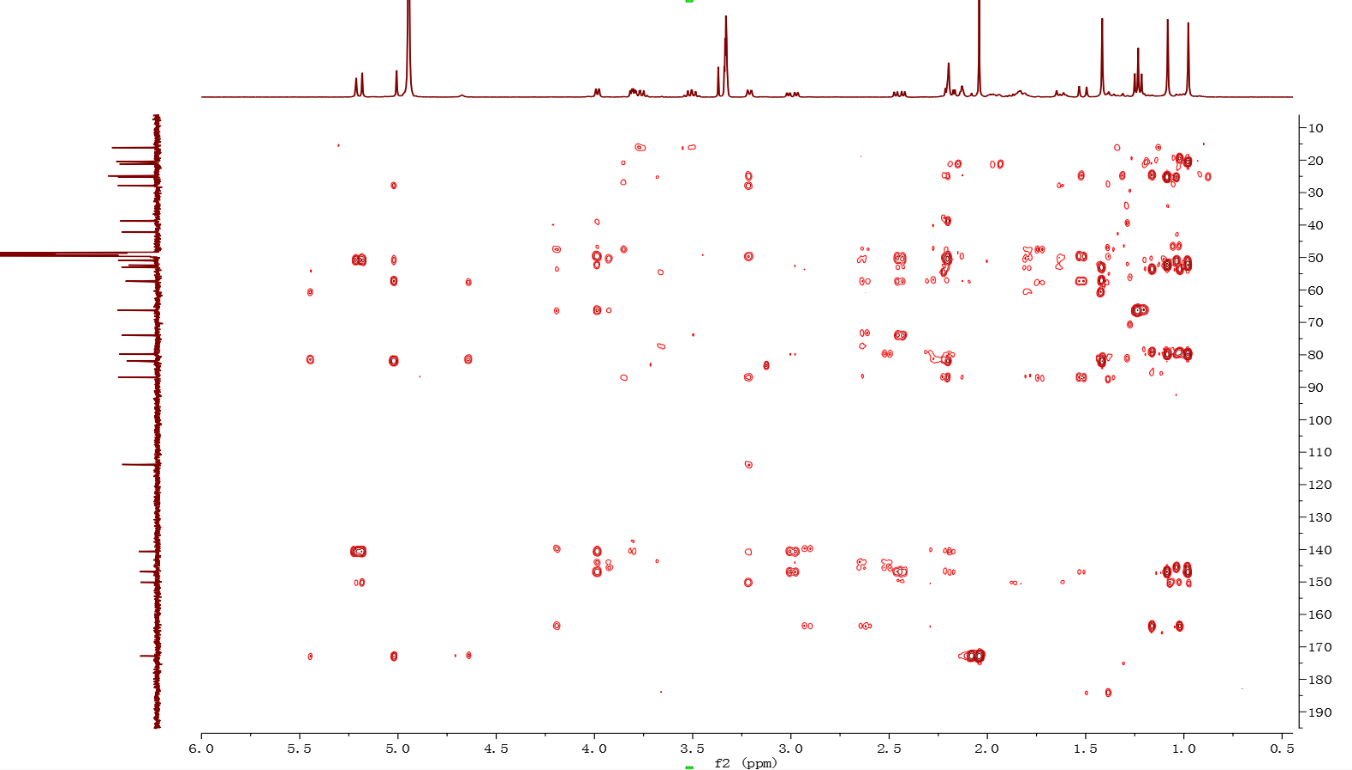


# ^1^H−^1^H COSY of compound 3


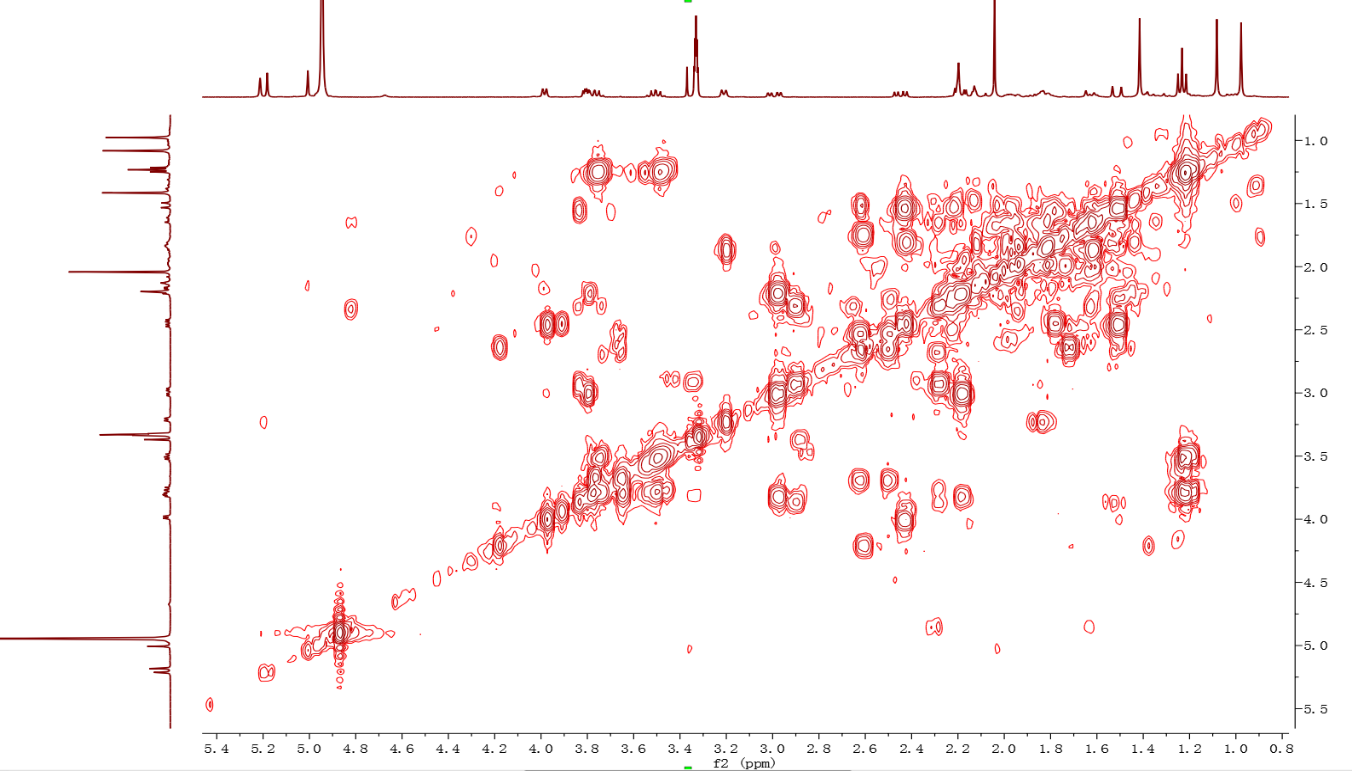


# NOESY of compound 3


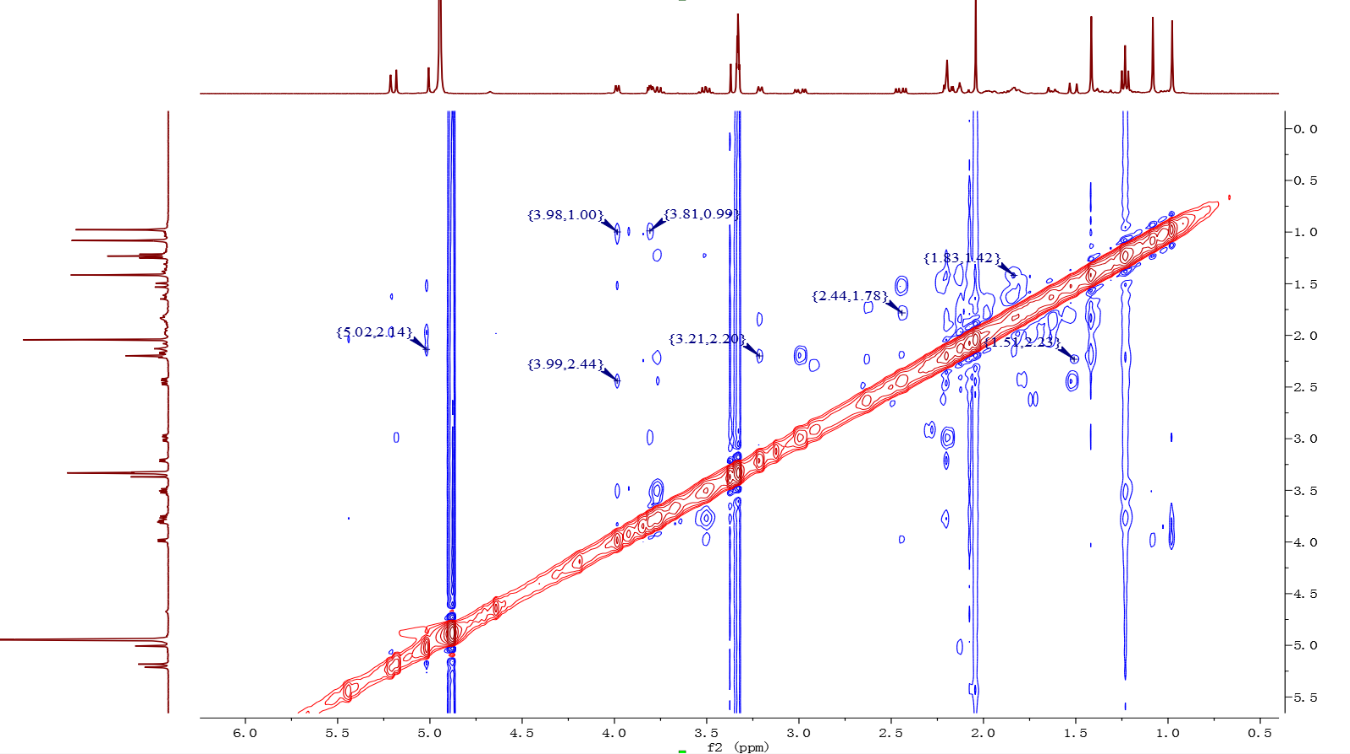


# HR-MS of compound 4

# CD spectrum of compound 4


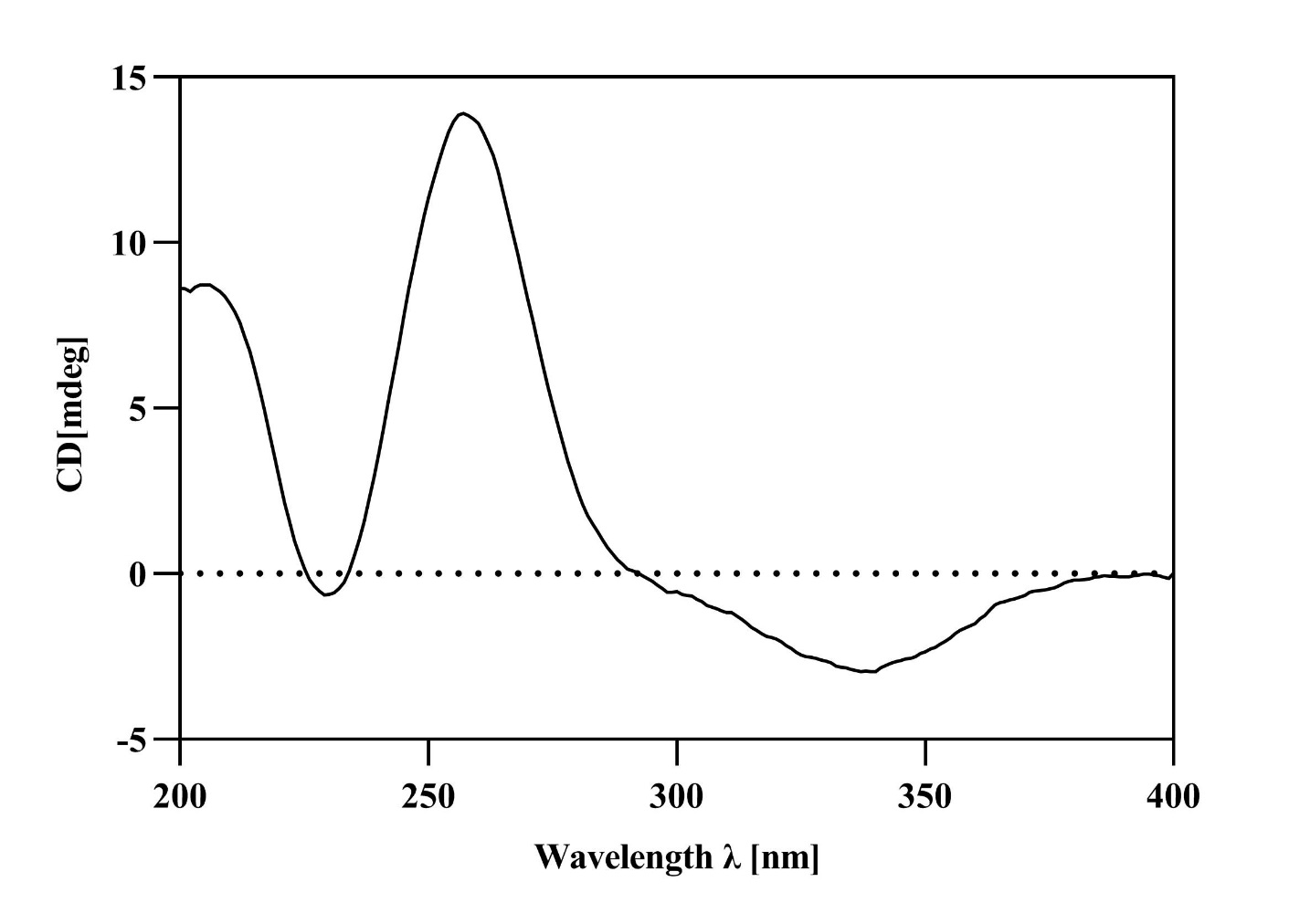


# ^1^H NMR of compound 4


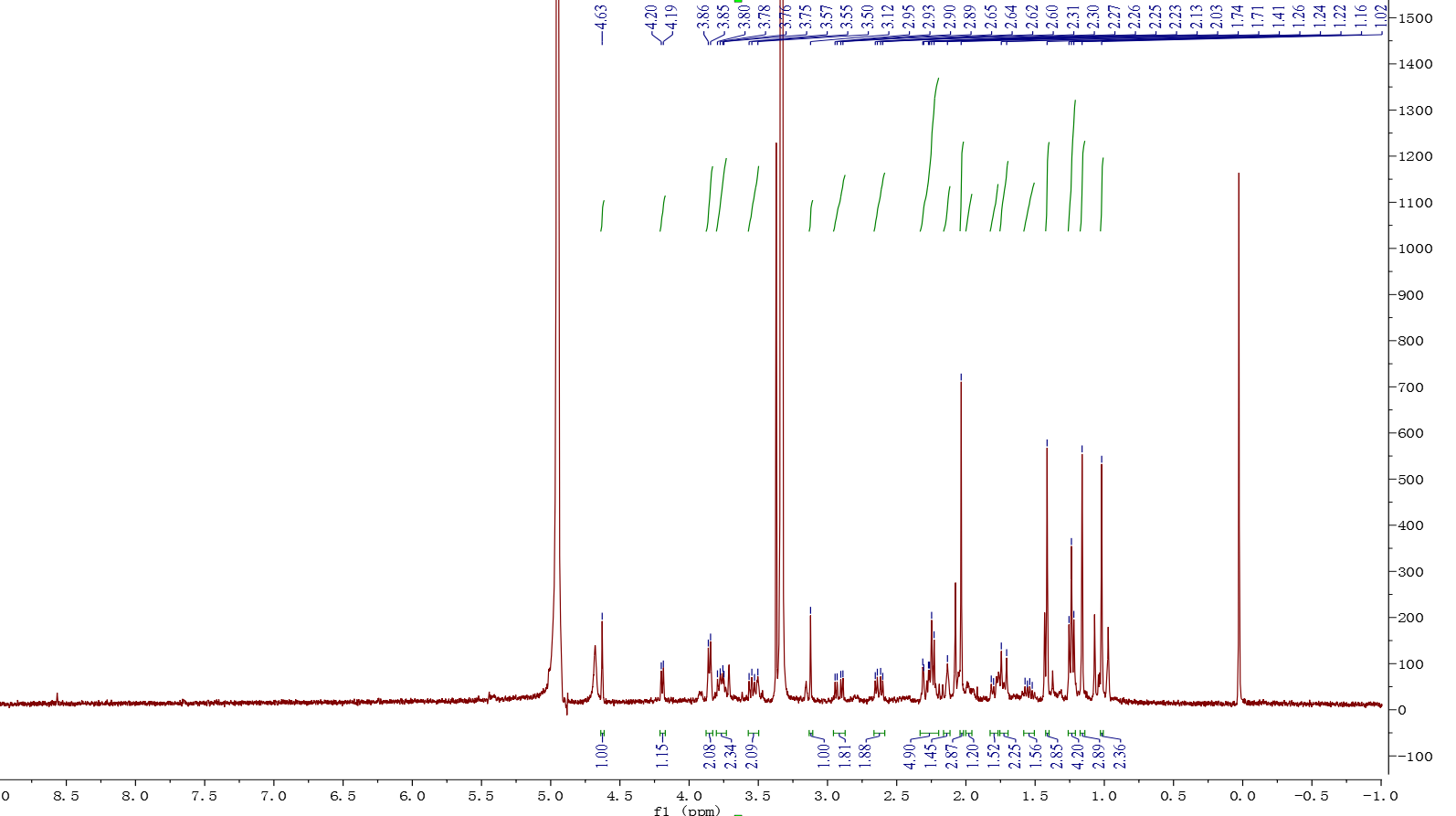


# ^13^C NMR of compound 4


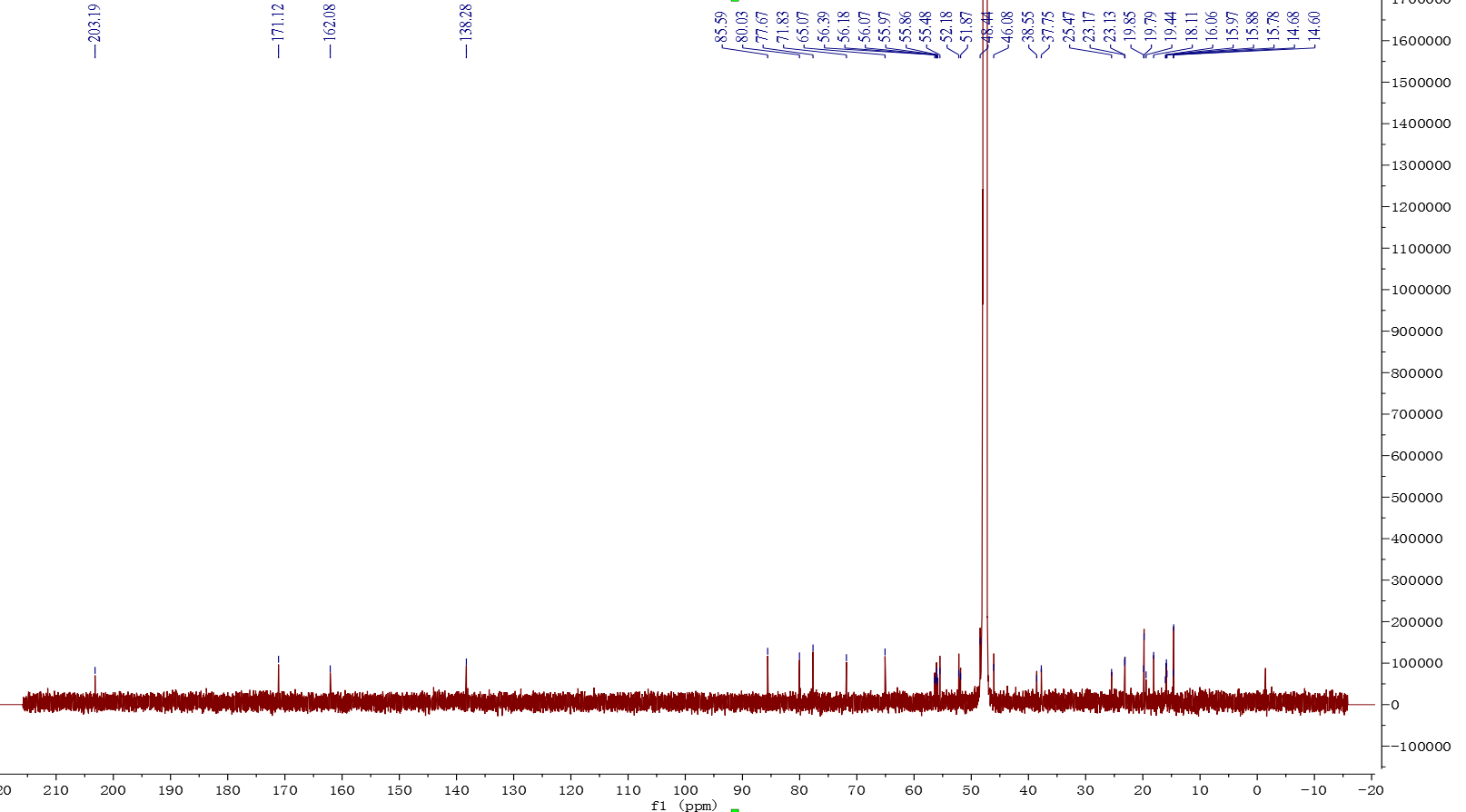


# HSQC of compound 4


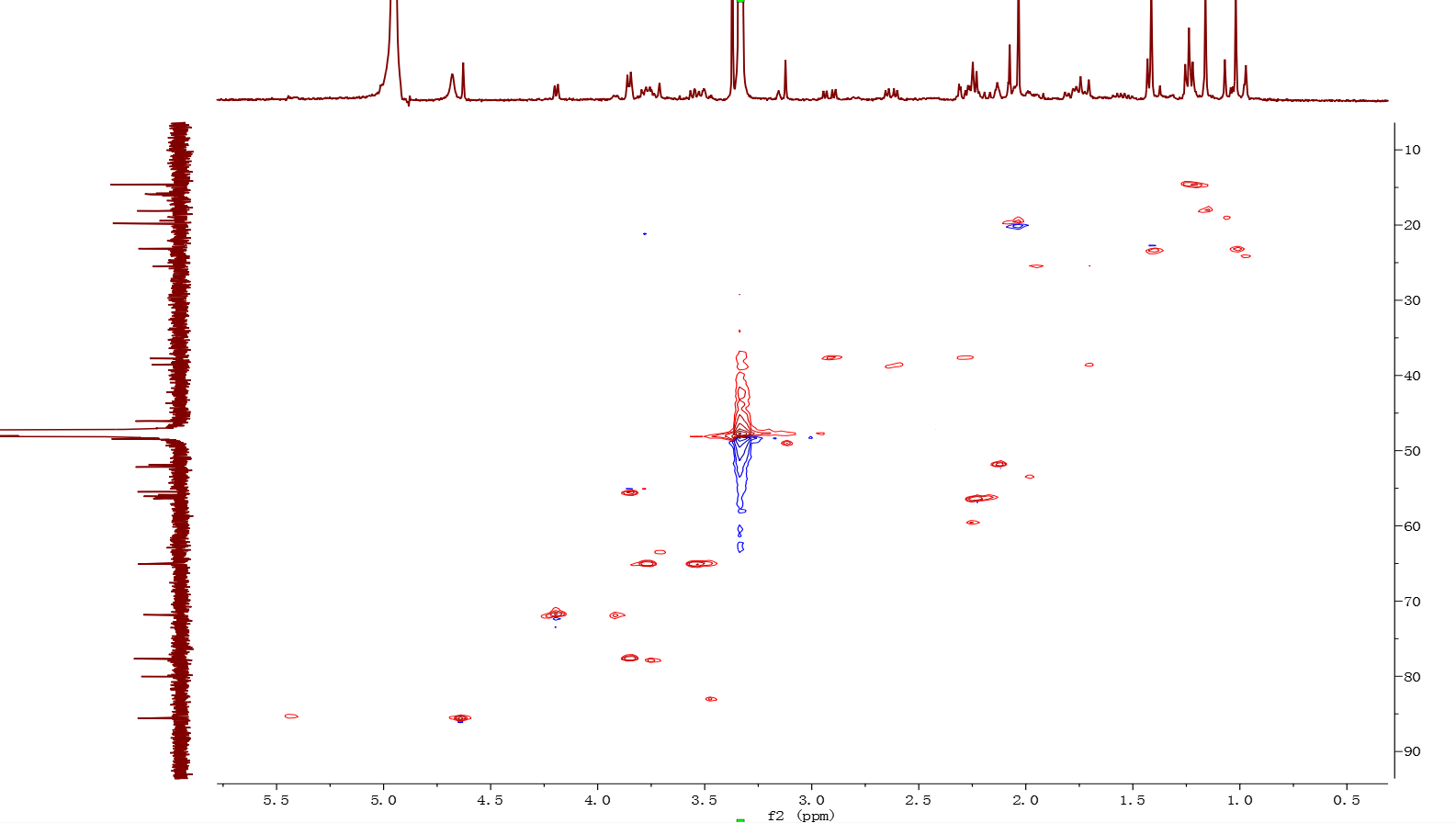


# HMBC of compound 4


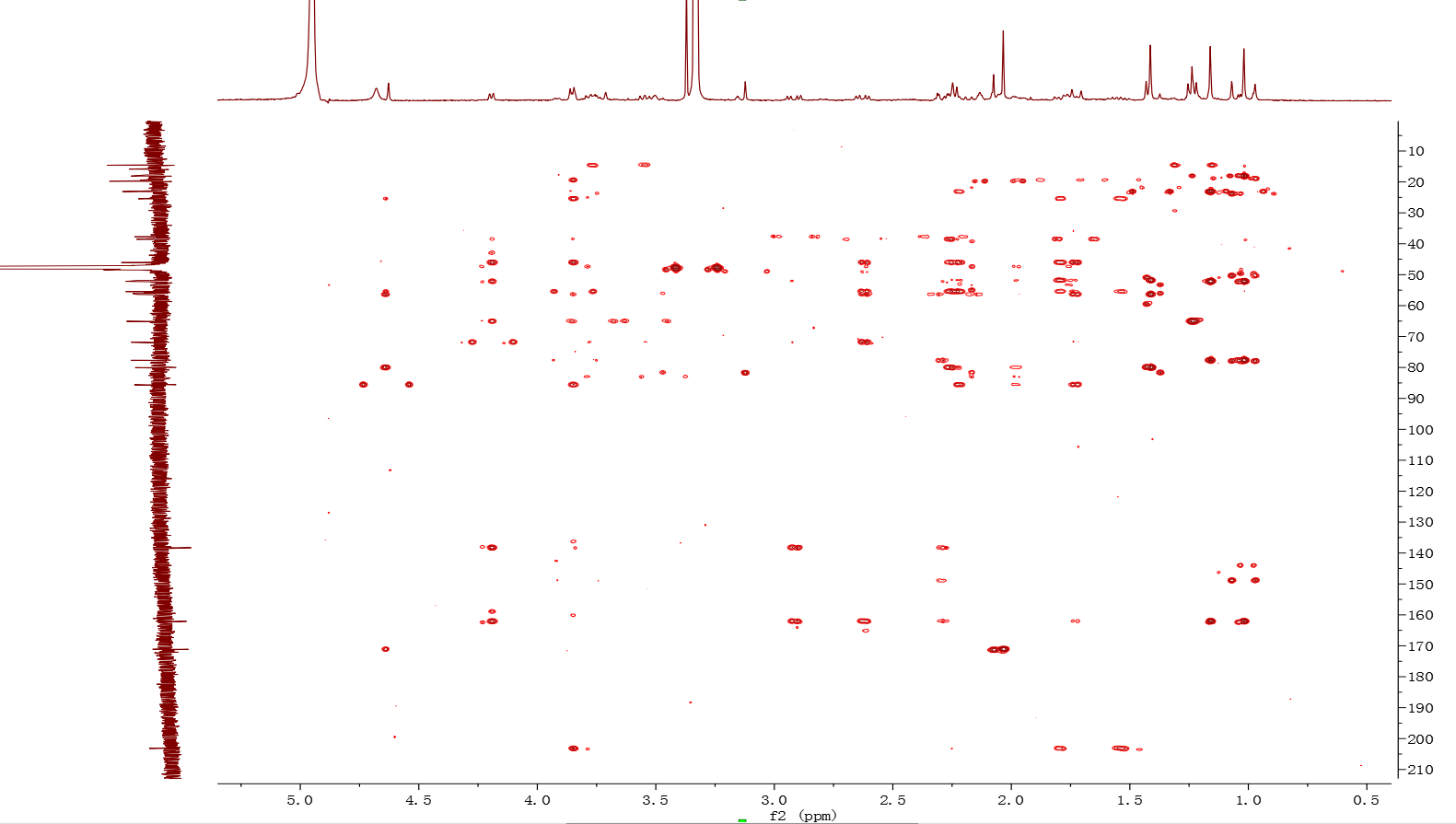


# ^1^H−^1^H COSY of compound 4


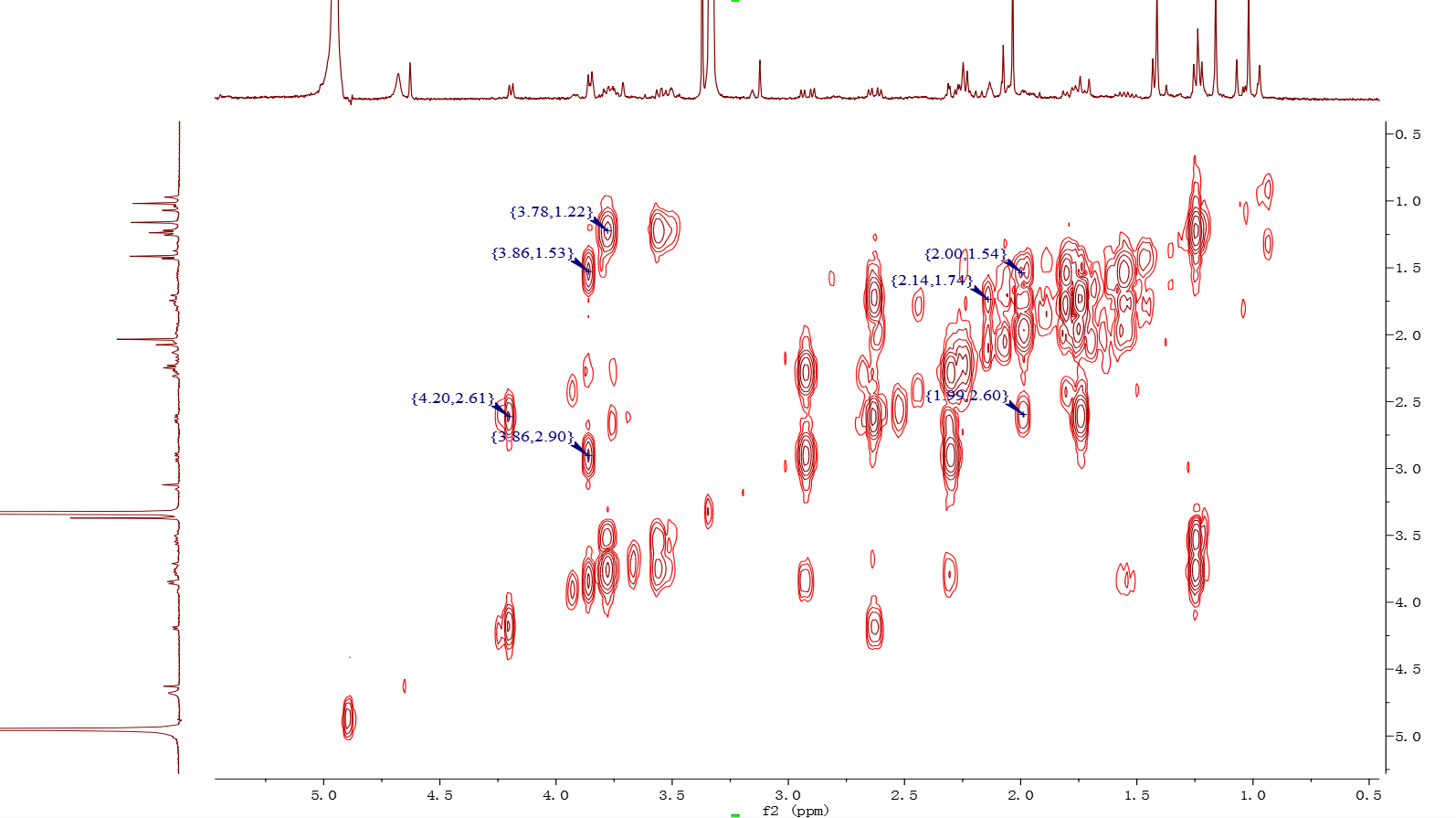


# NOESY of compound 4


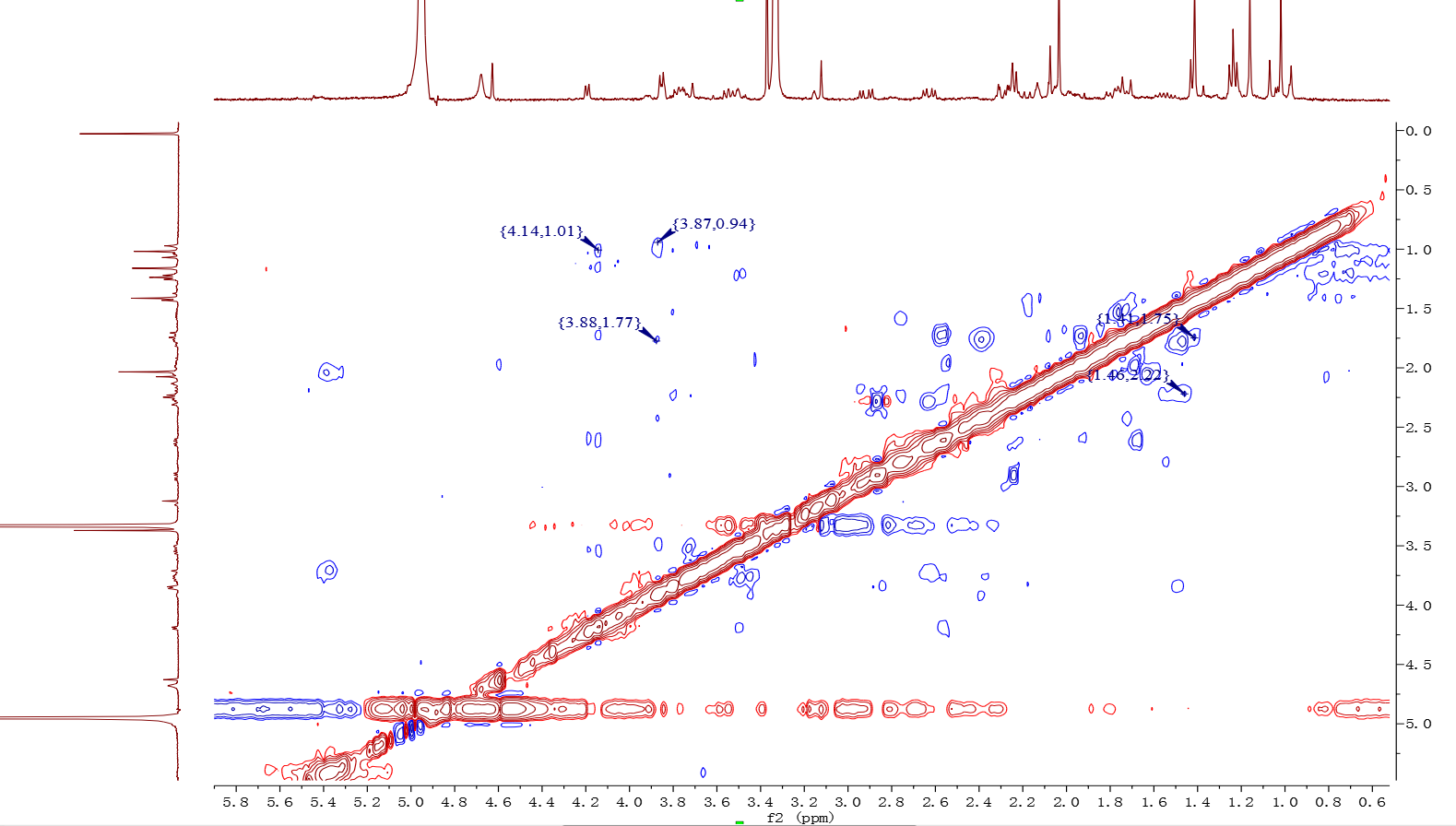


# HR-MS of compound 13

# CD spectrum of compound 13


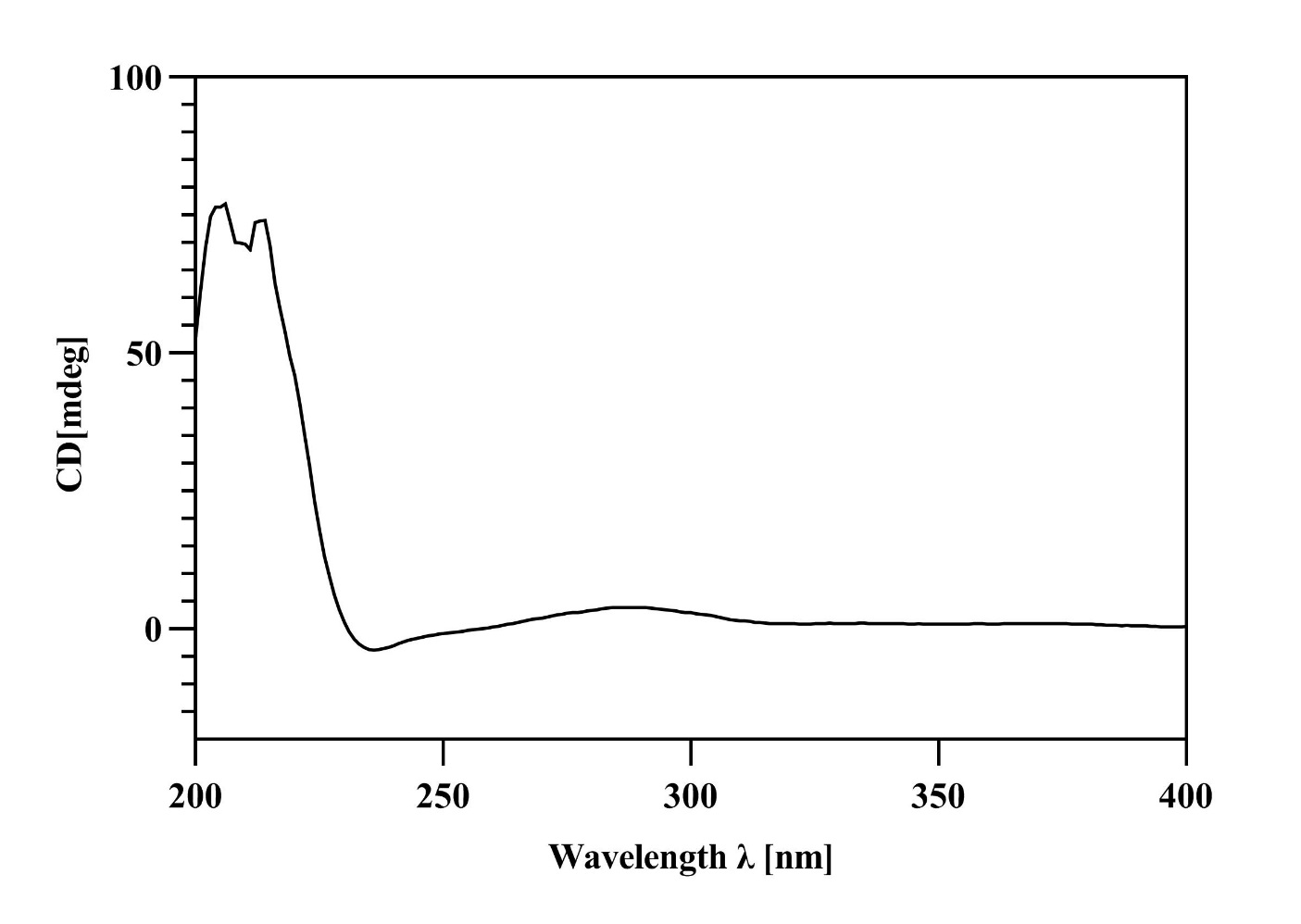


# ^1^H NMR of compound 13


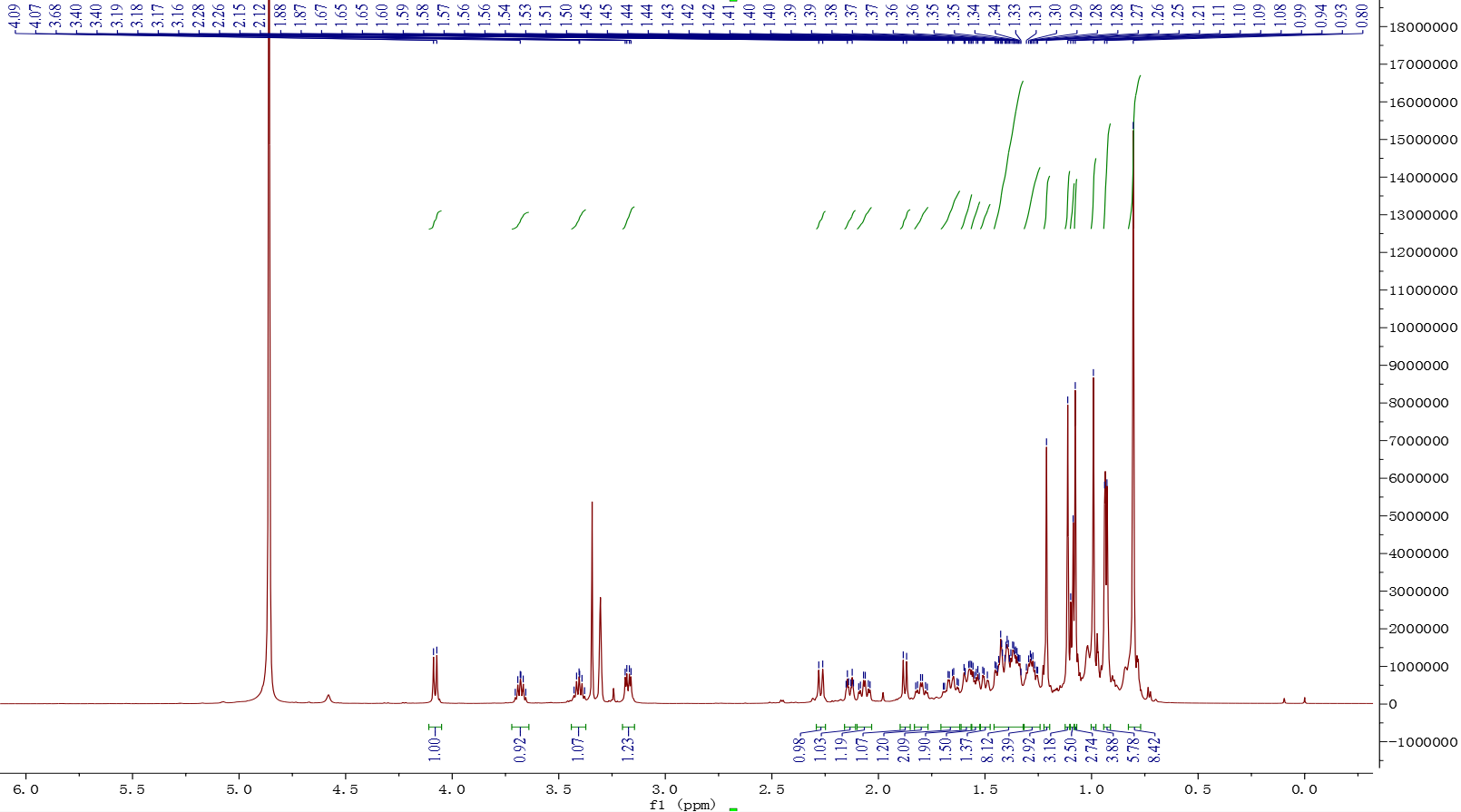


# ^13^C NMR of compound 13


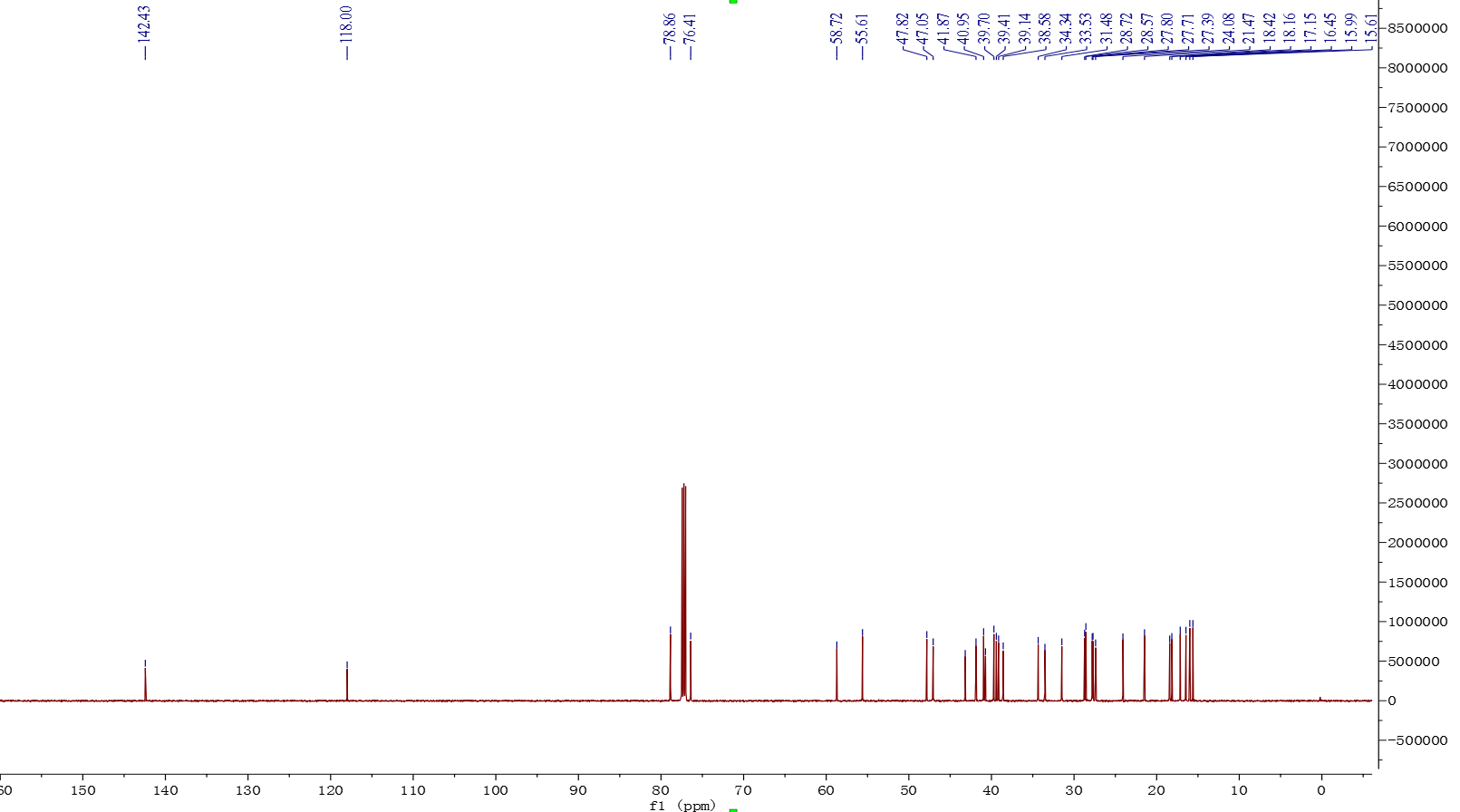


# HSQC of compound 13


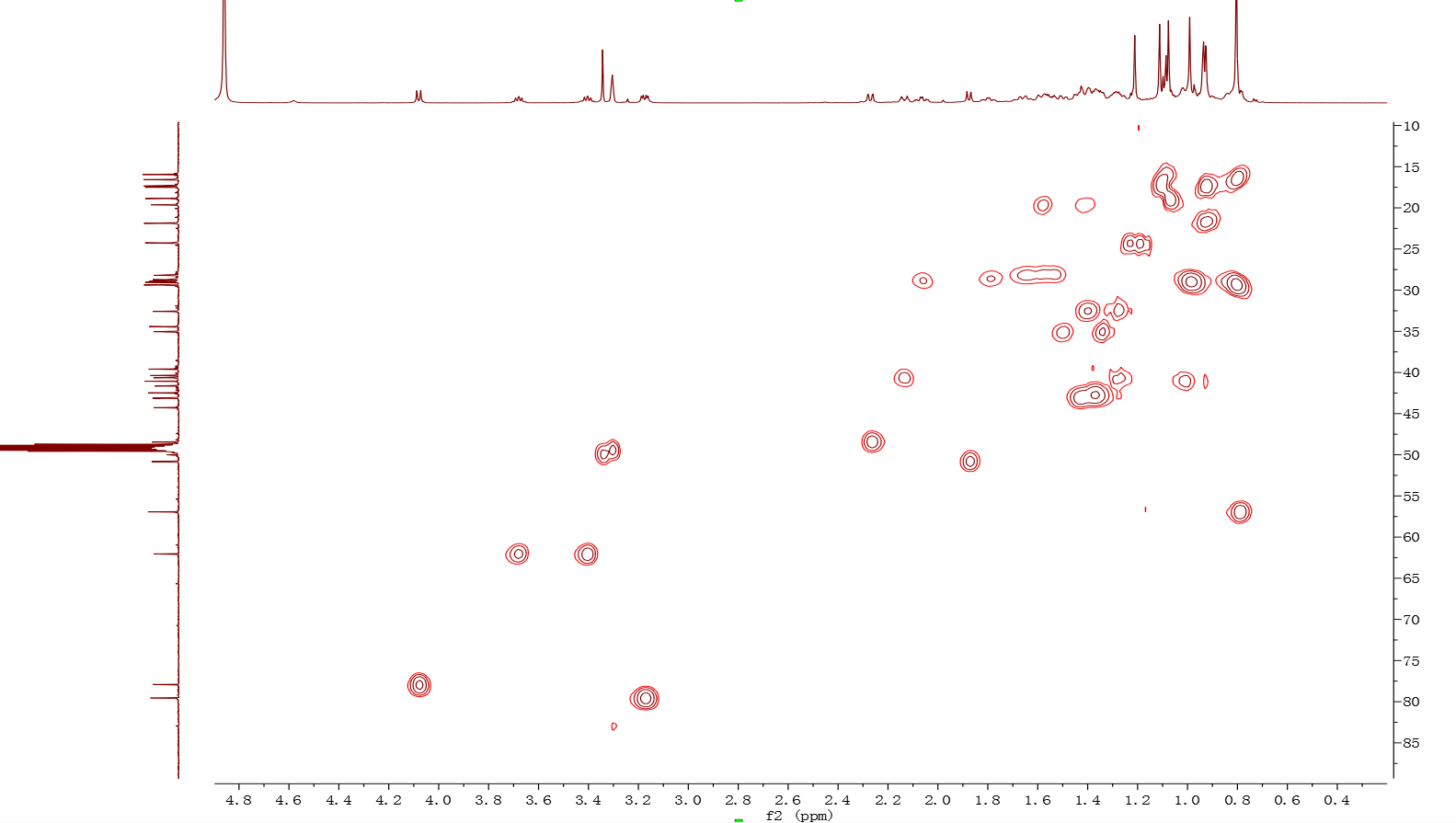


# HMBC of compound 13


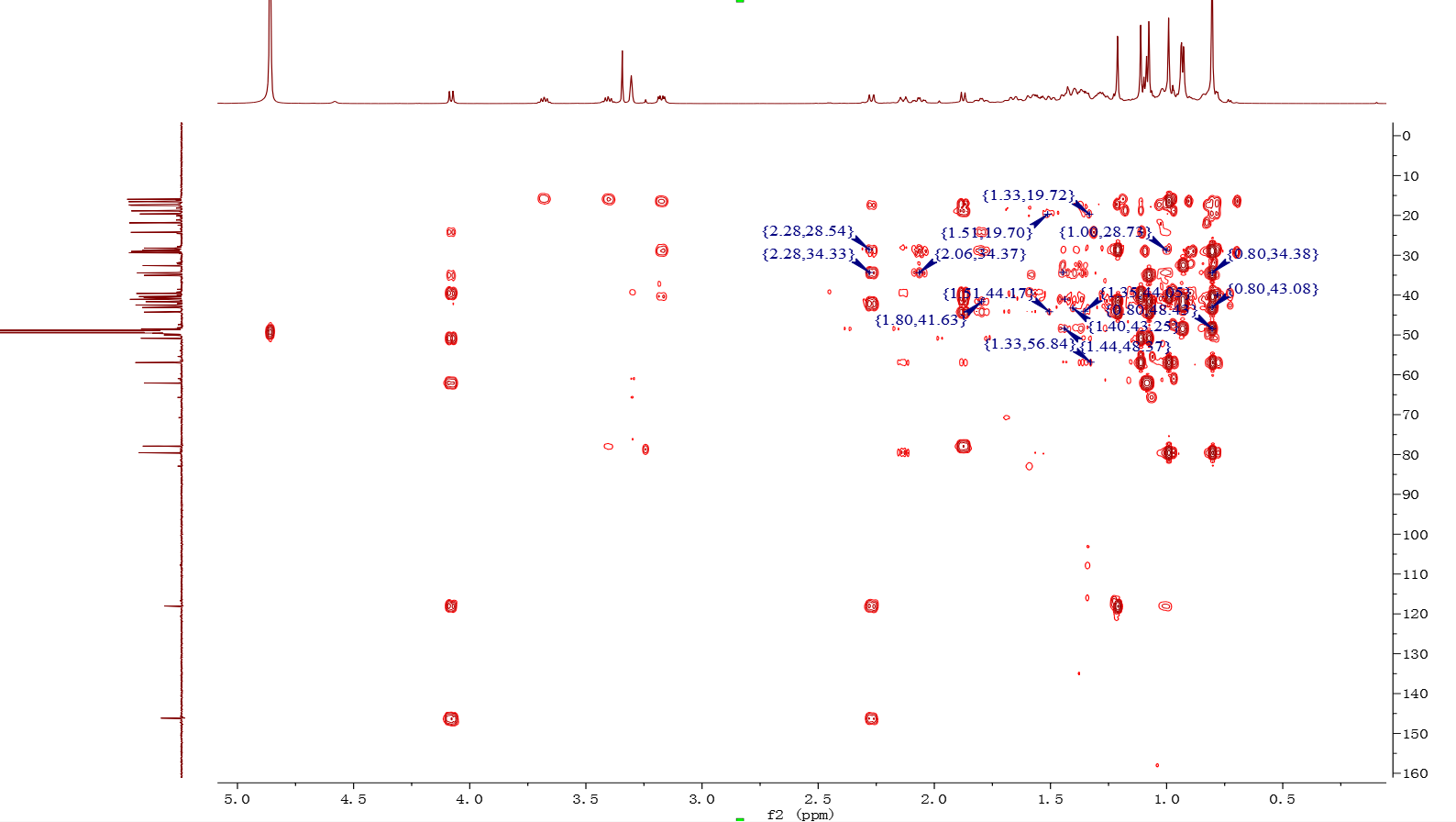


# ^1^H−^1^H COSY of compound 13


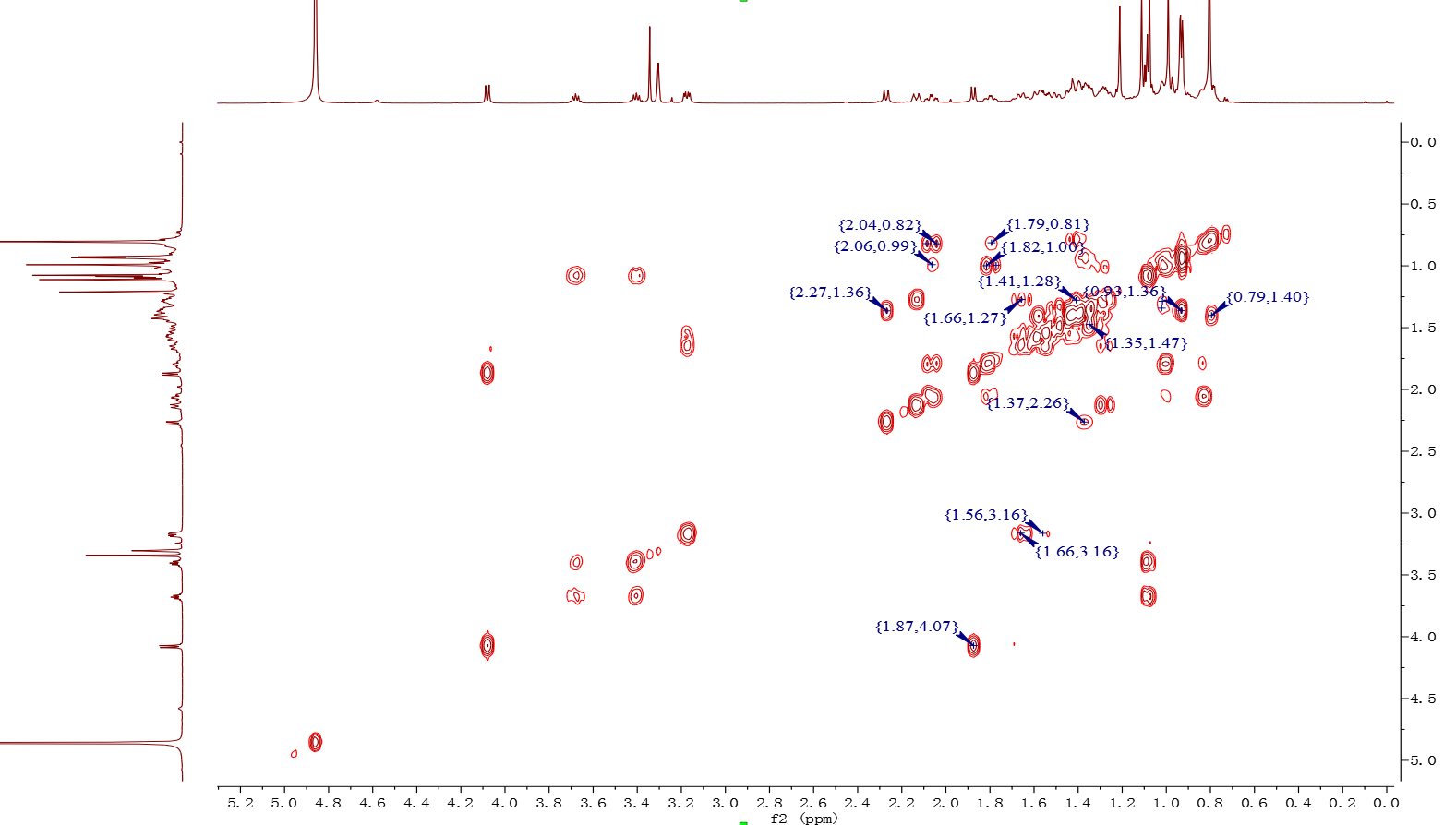


# NOESY of compound 13


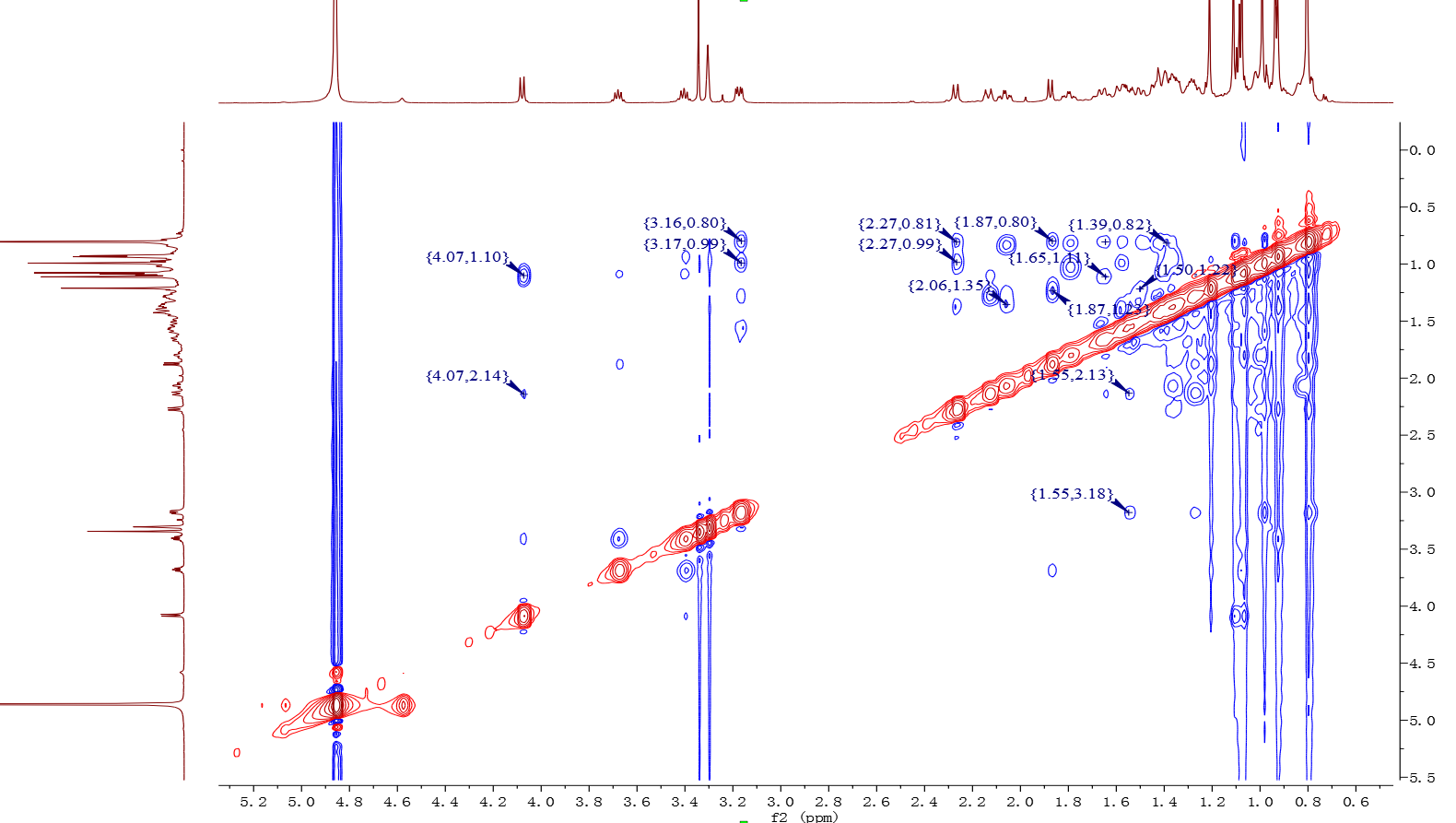


# HR-MS of compound 14

# CD spectrum of compound 14


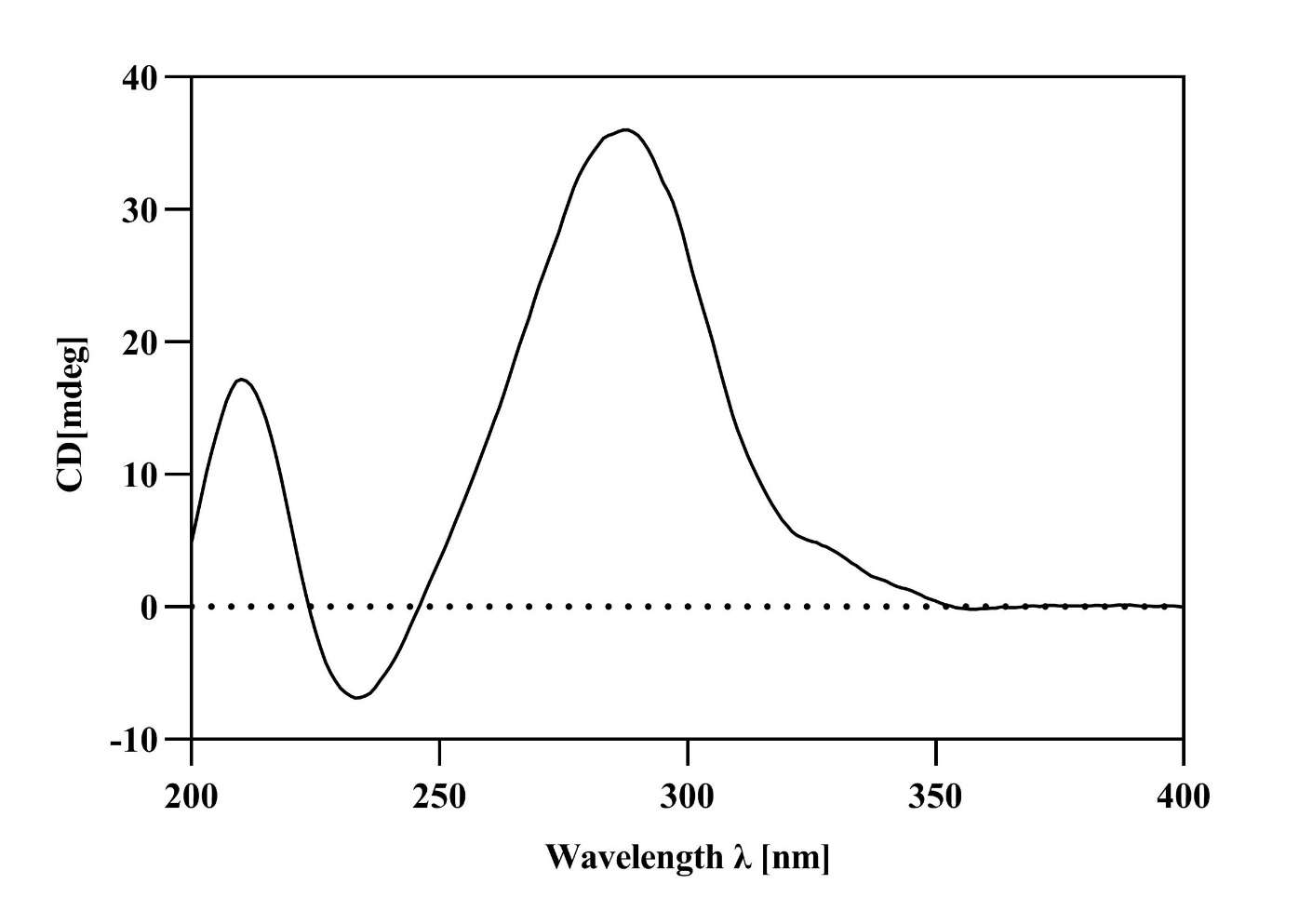


# ^1^H NMR of compound 14


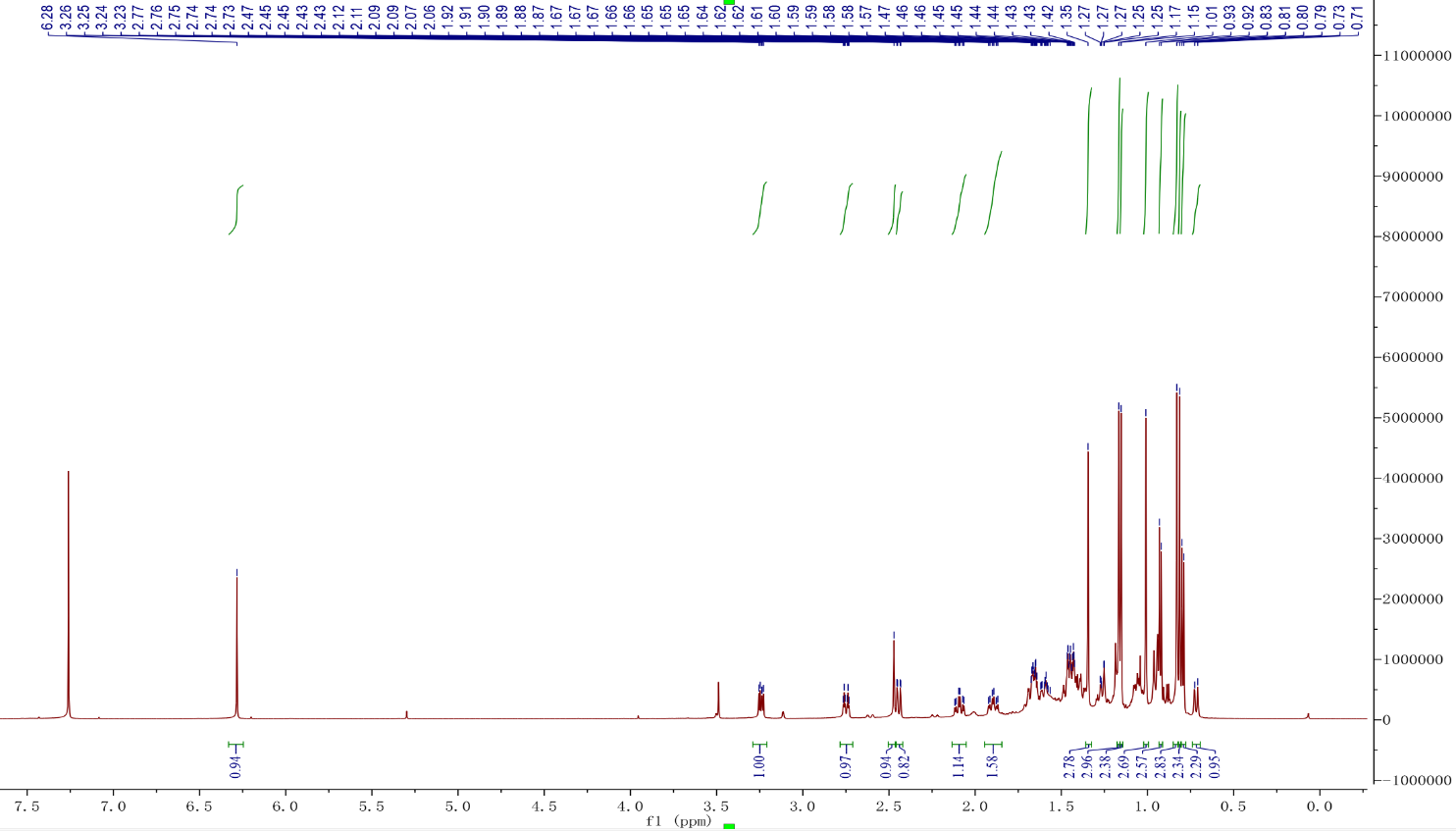


# ^13^C NMR of compound 14


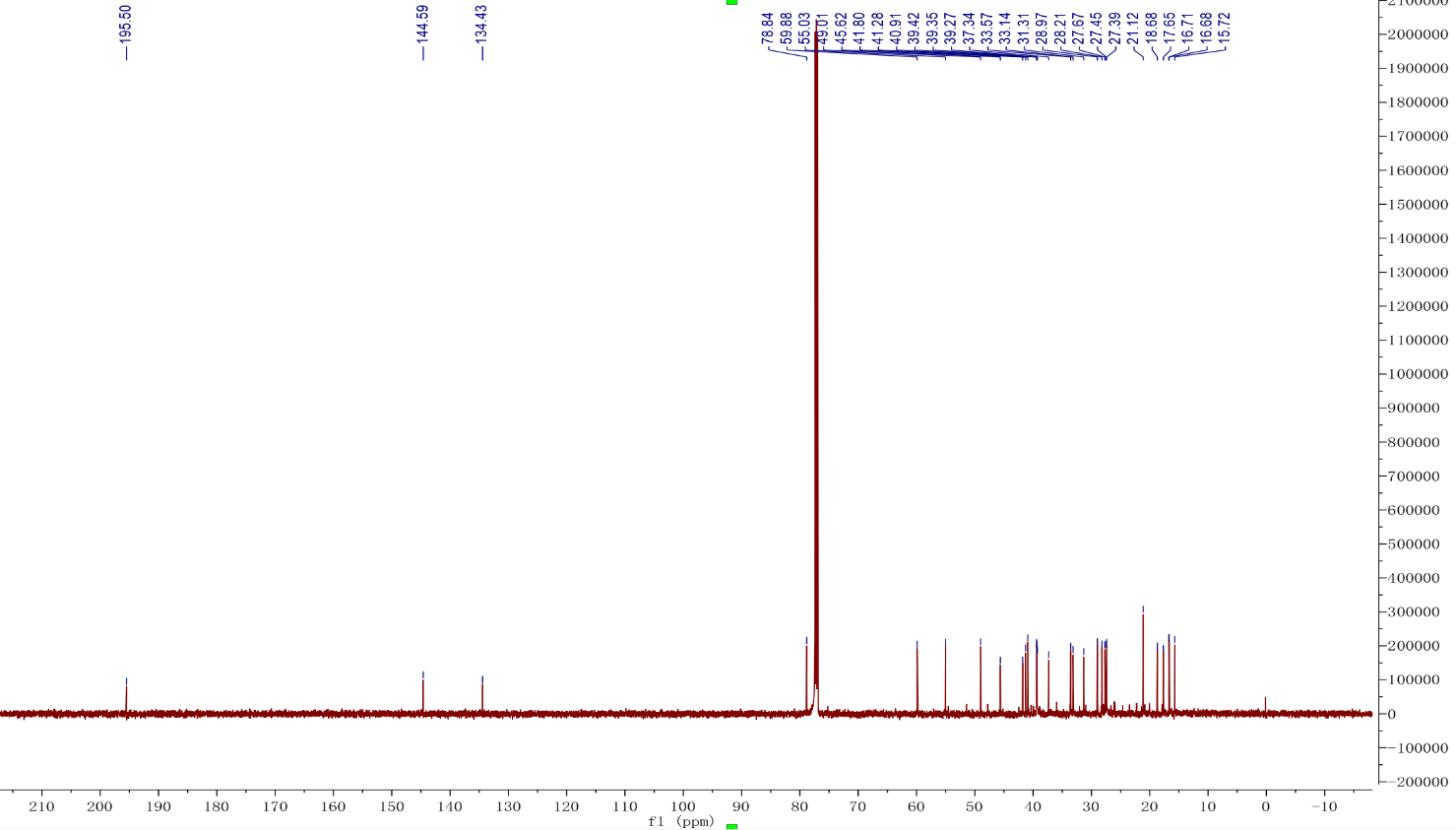


# HSQC of compound 14


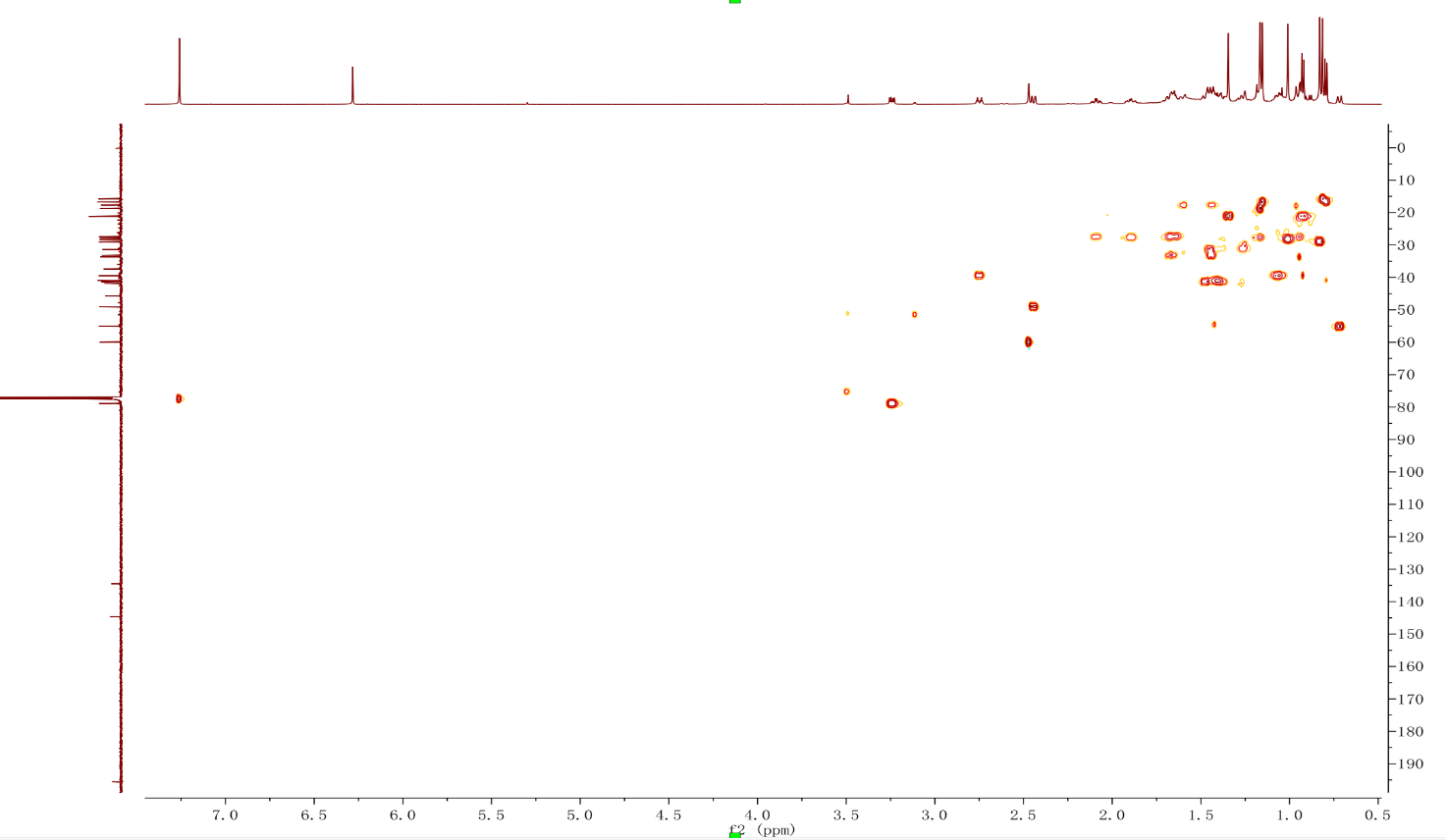


# HMBC of compound 14


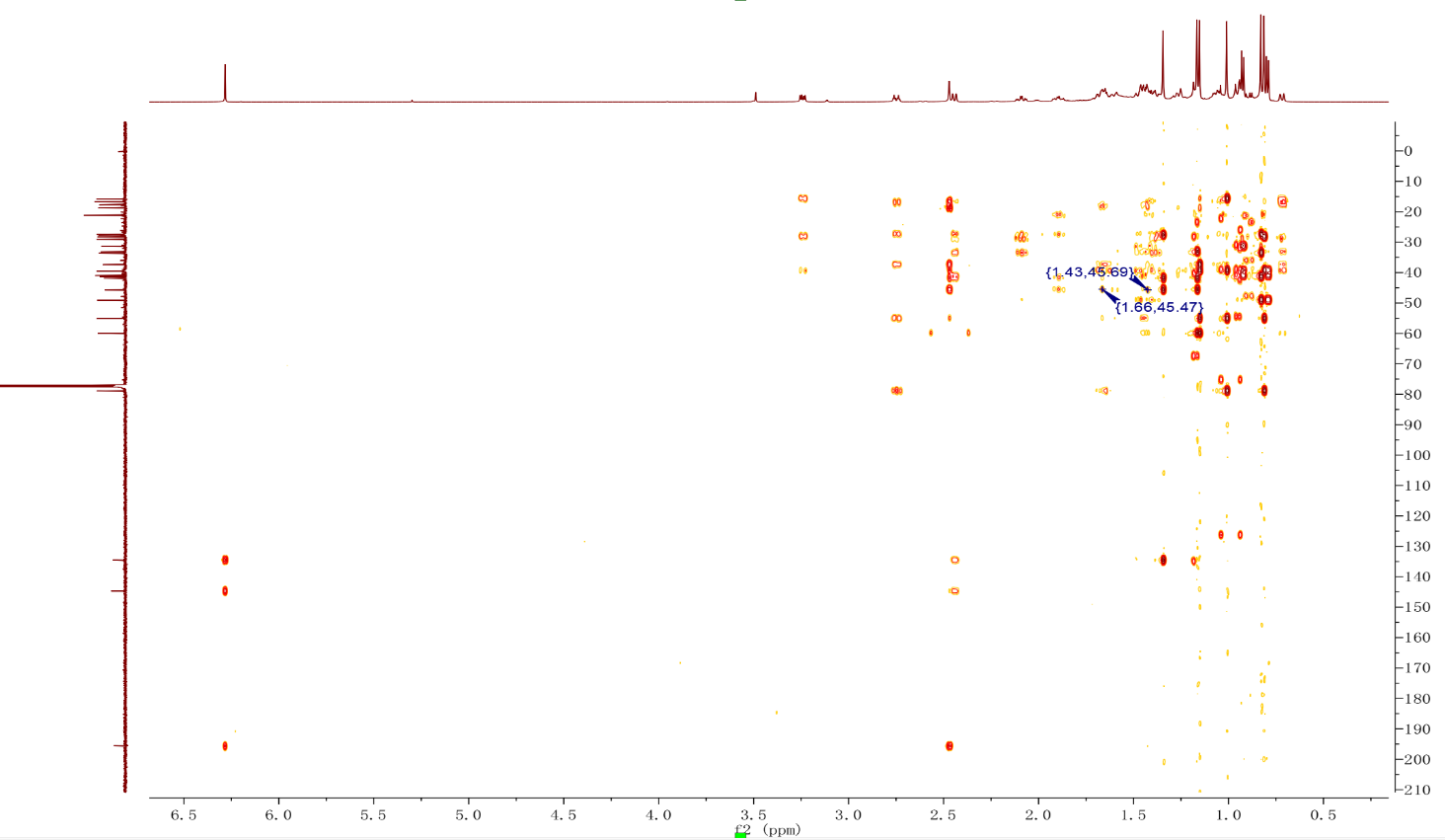


# ^1^H−^1^H COSY of compound 14


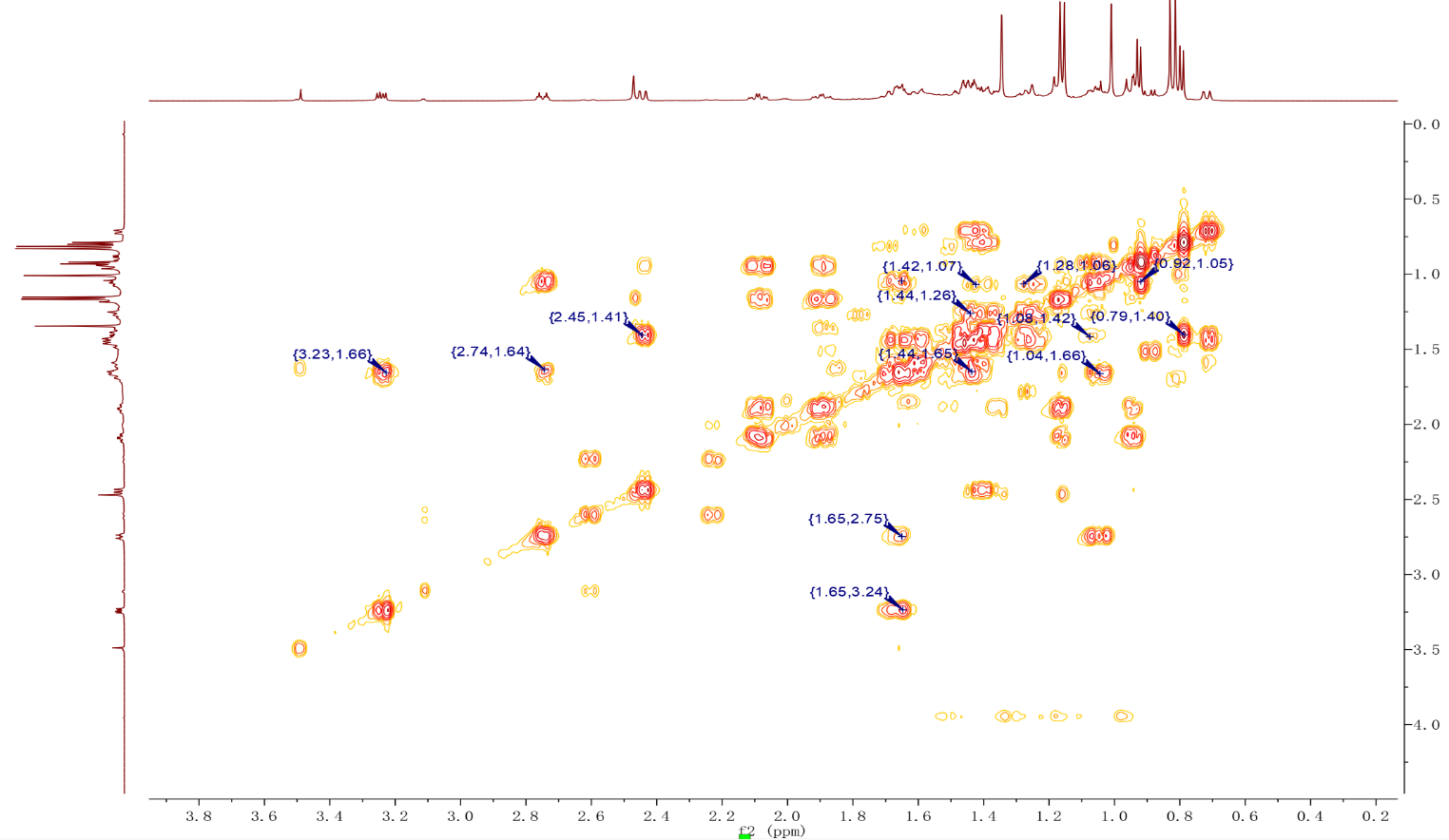


# NOESY of compound 14


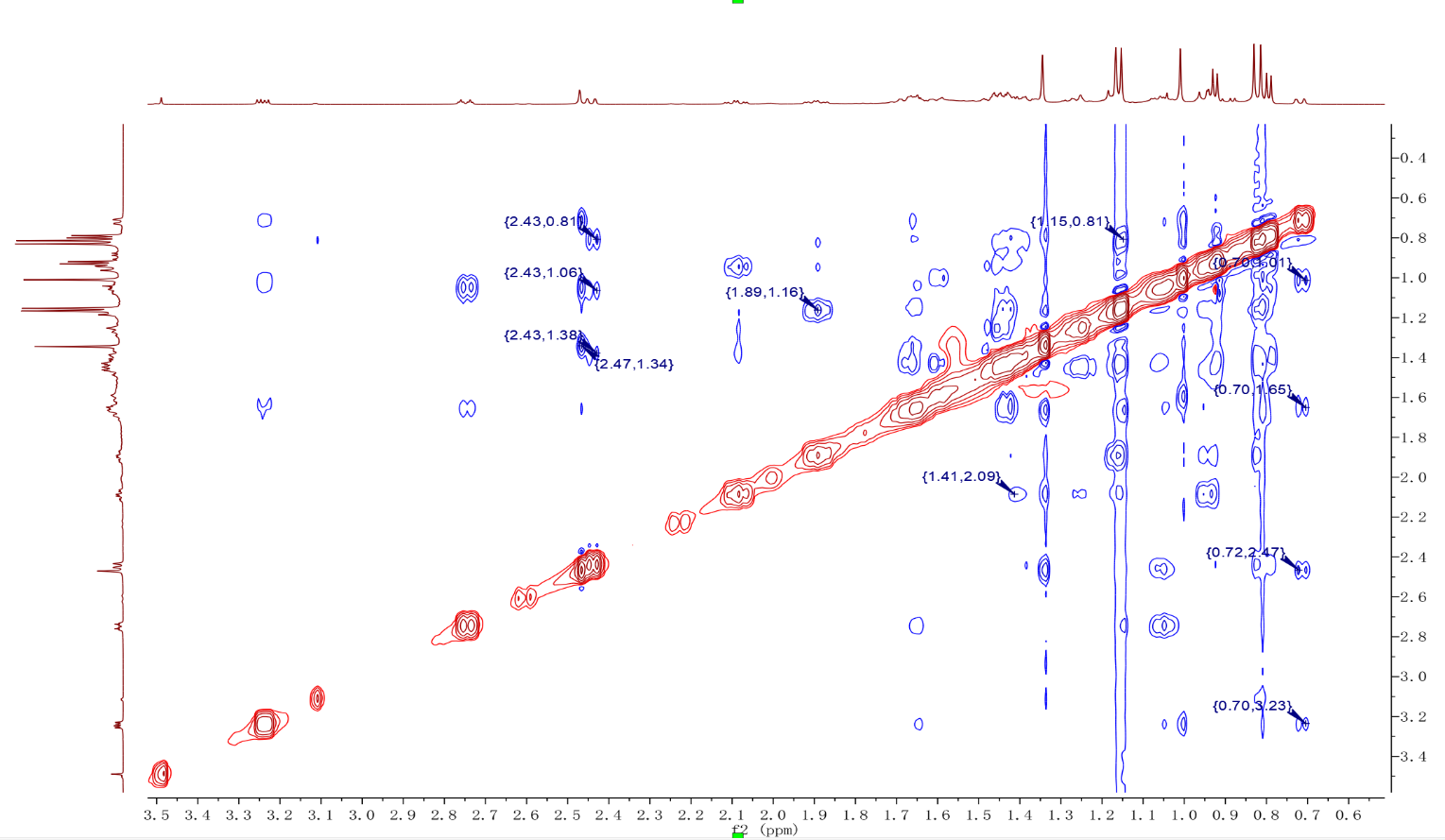


# HR-MS of compound 15

# CD spectrum of compound 15


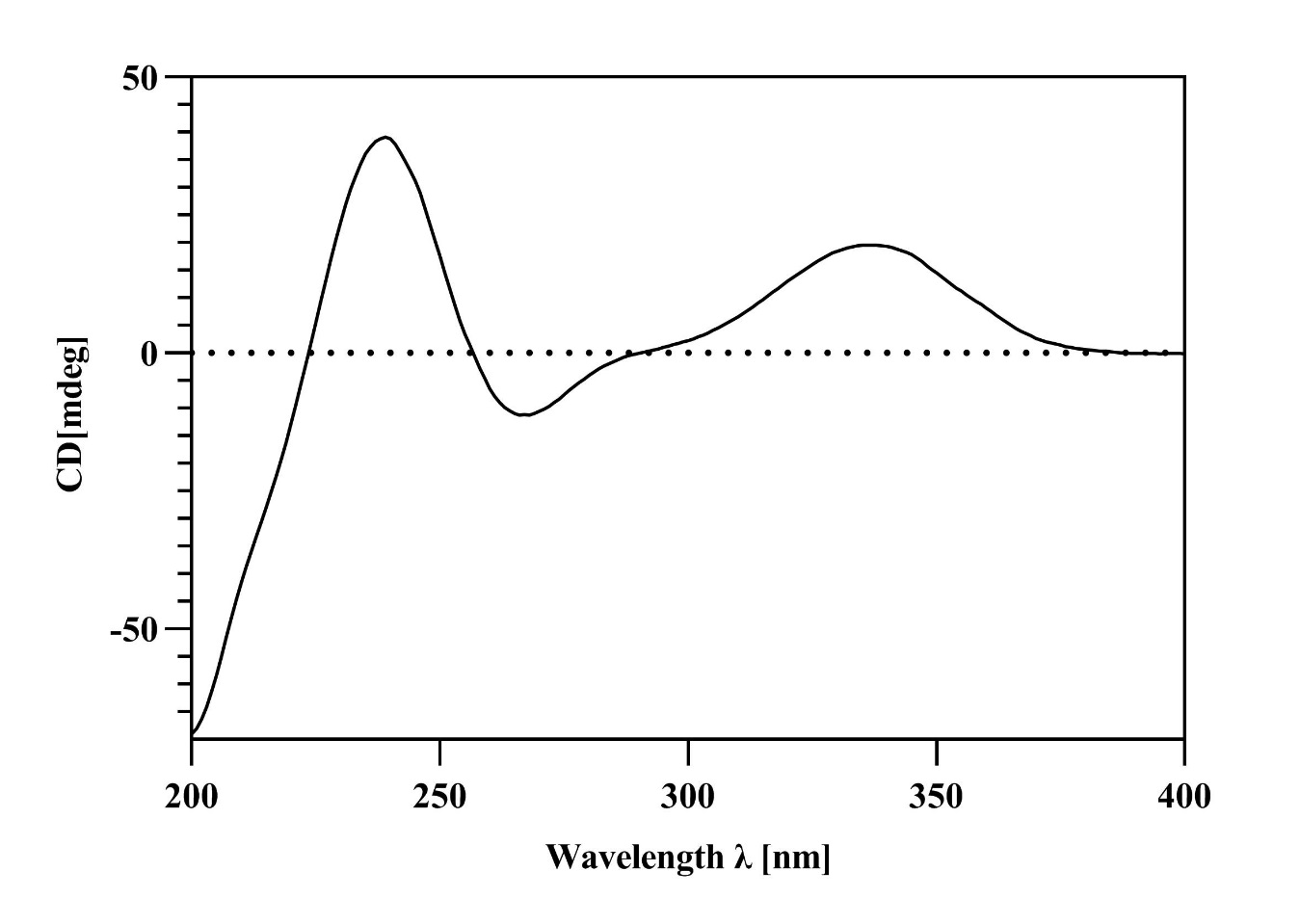


# ^1^H NMR of compound 15


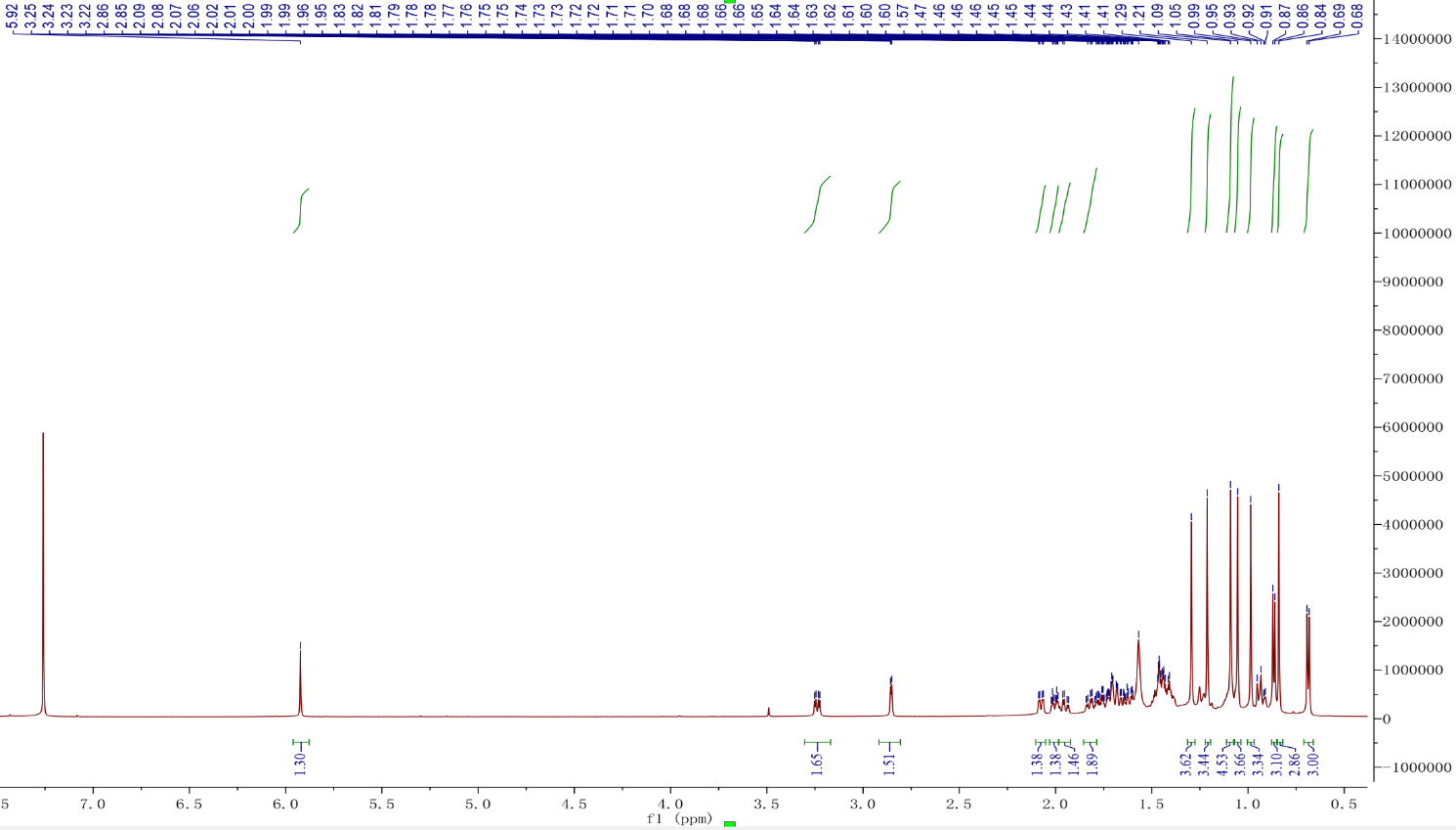


# ^13^C NMR of compound 15


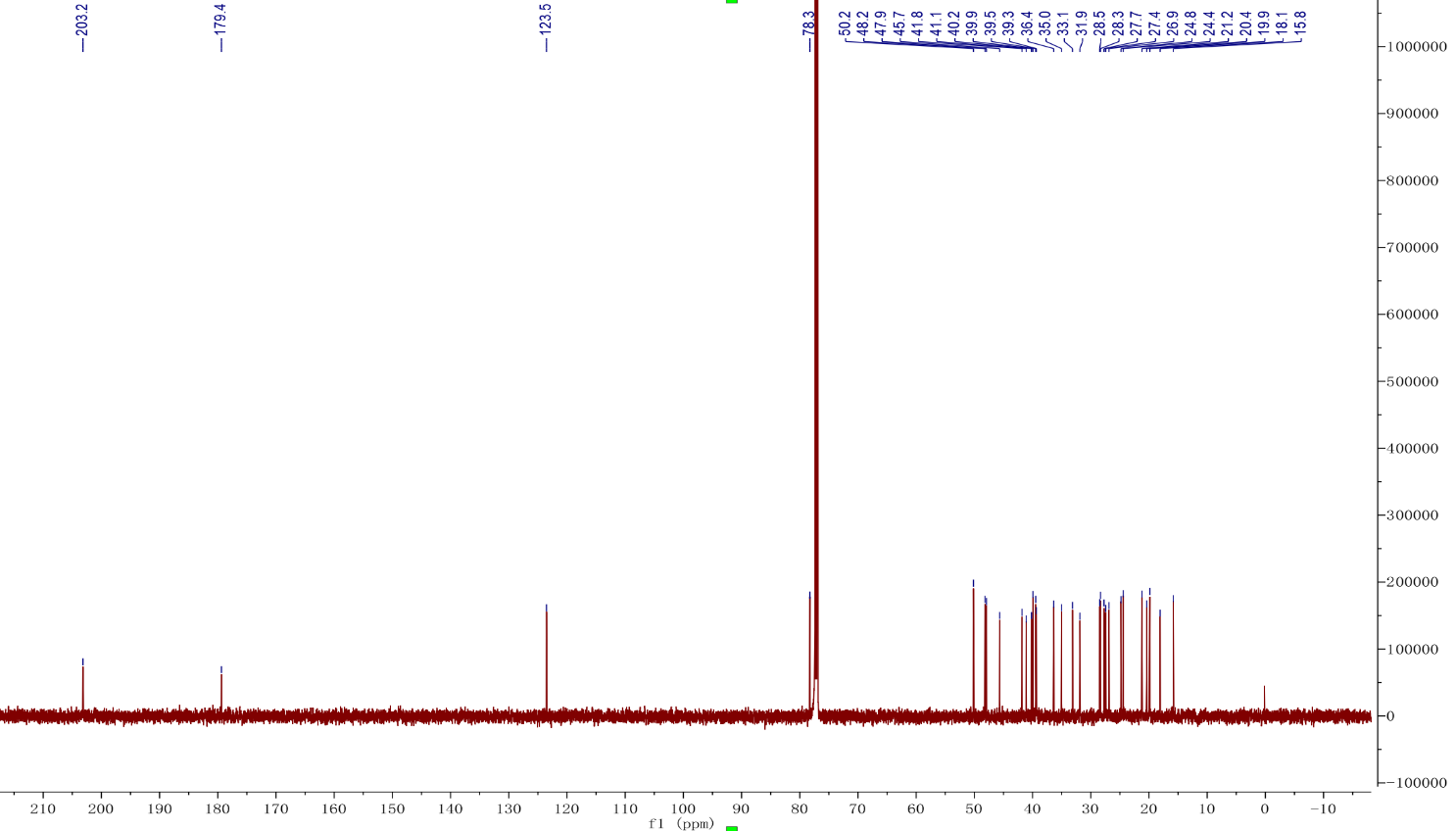


# HSQC of compound 15


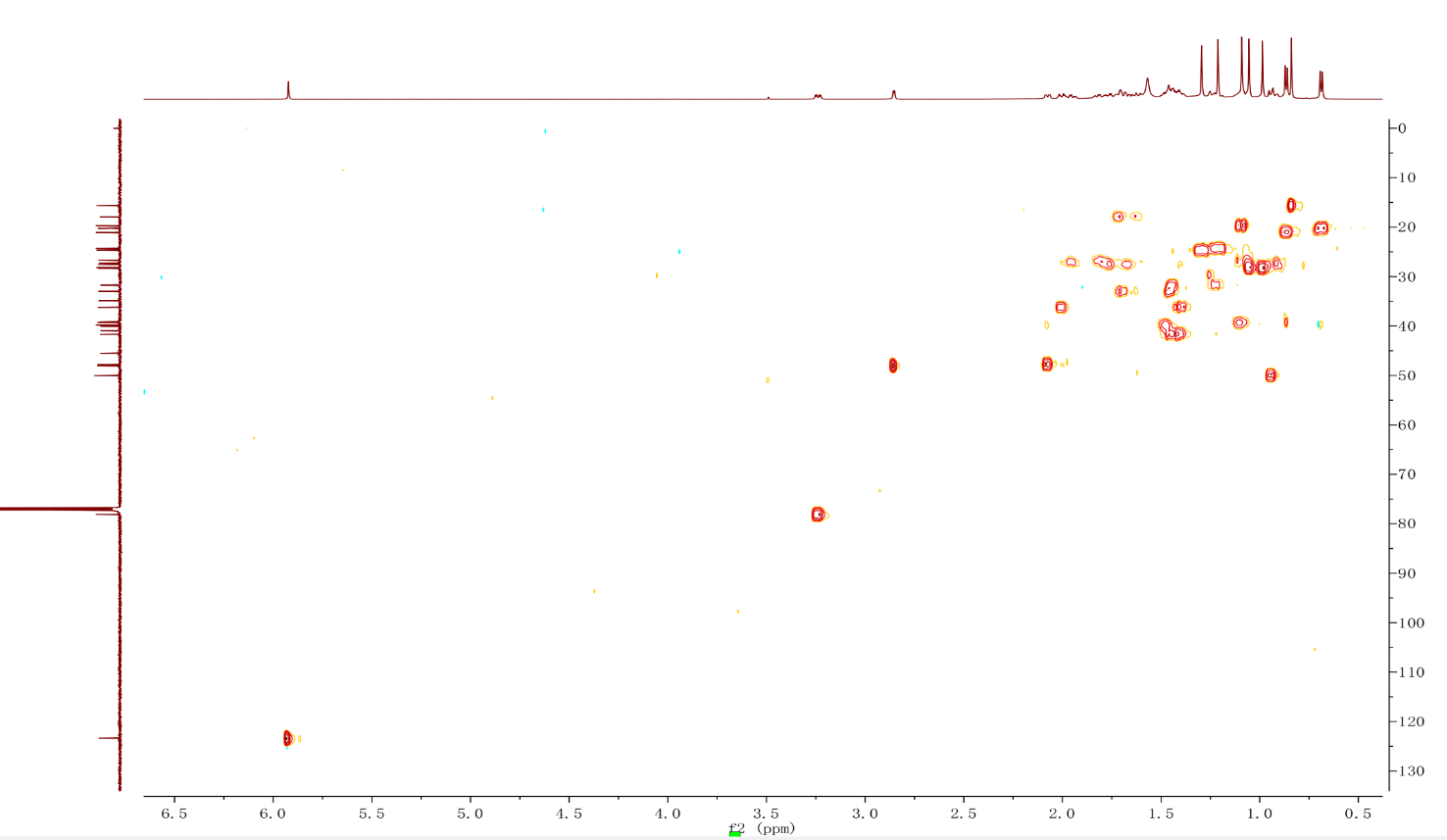


# HMBC of compound 15


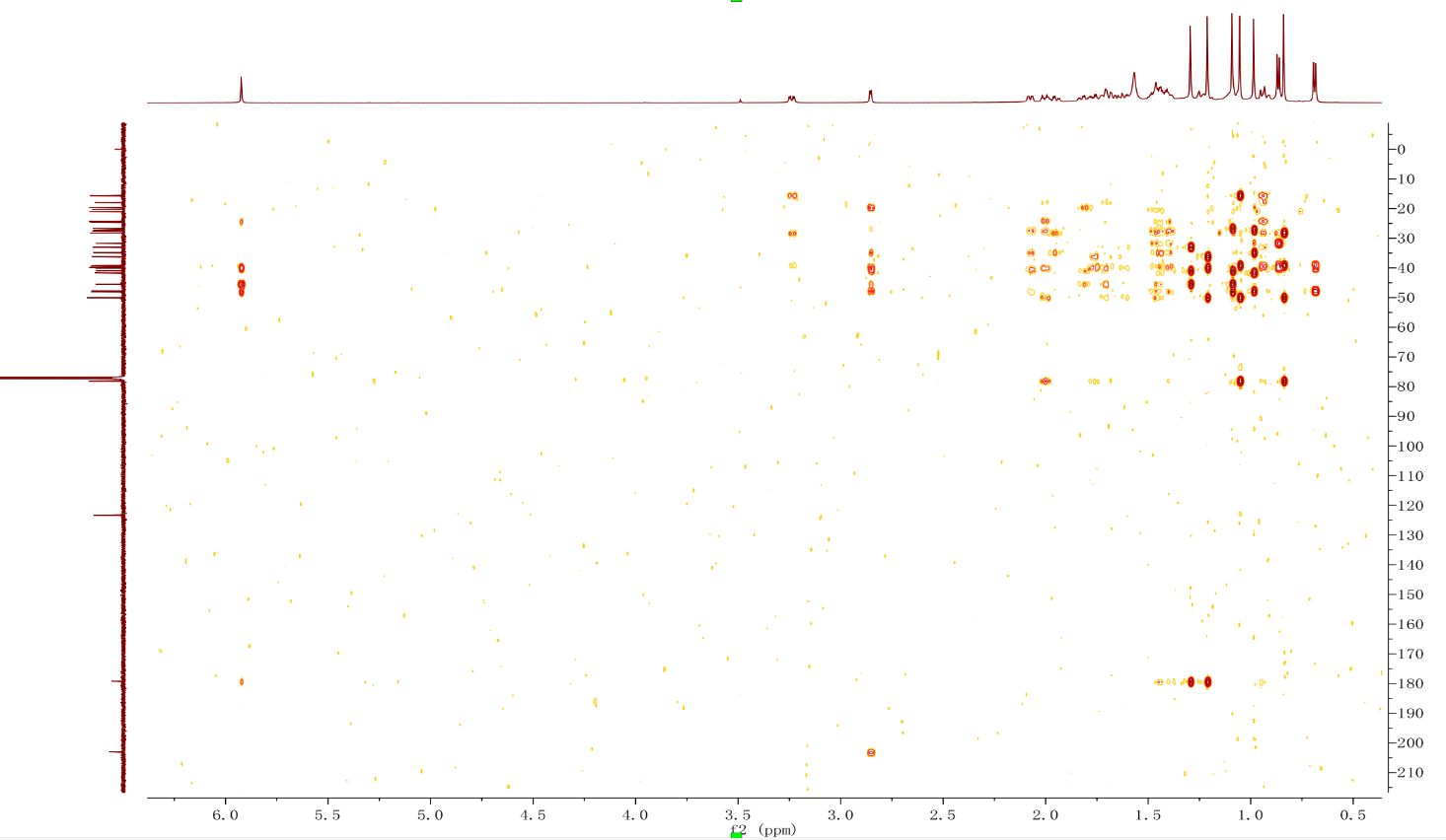


# ^1^H−^1^H COSY of compound 15


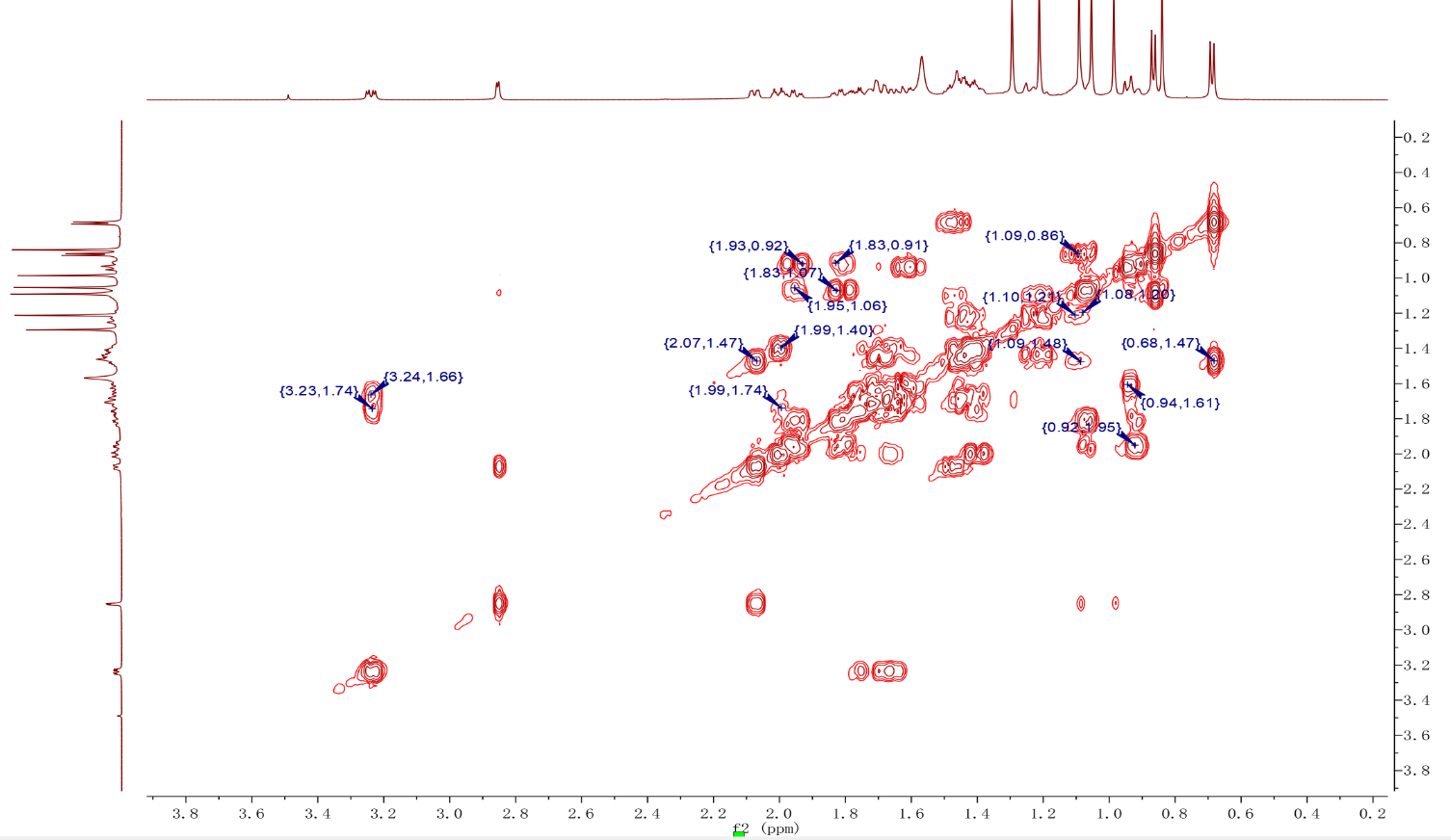


# NOESY of compound 15


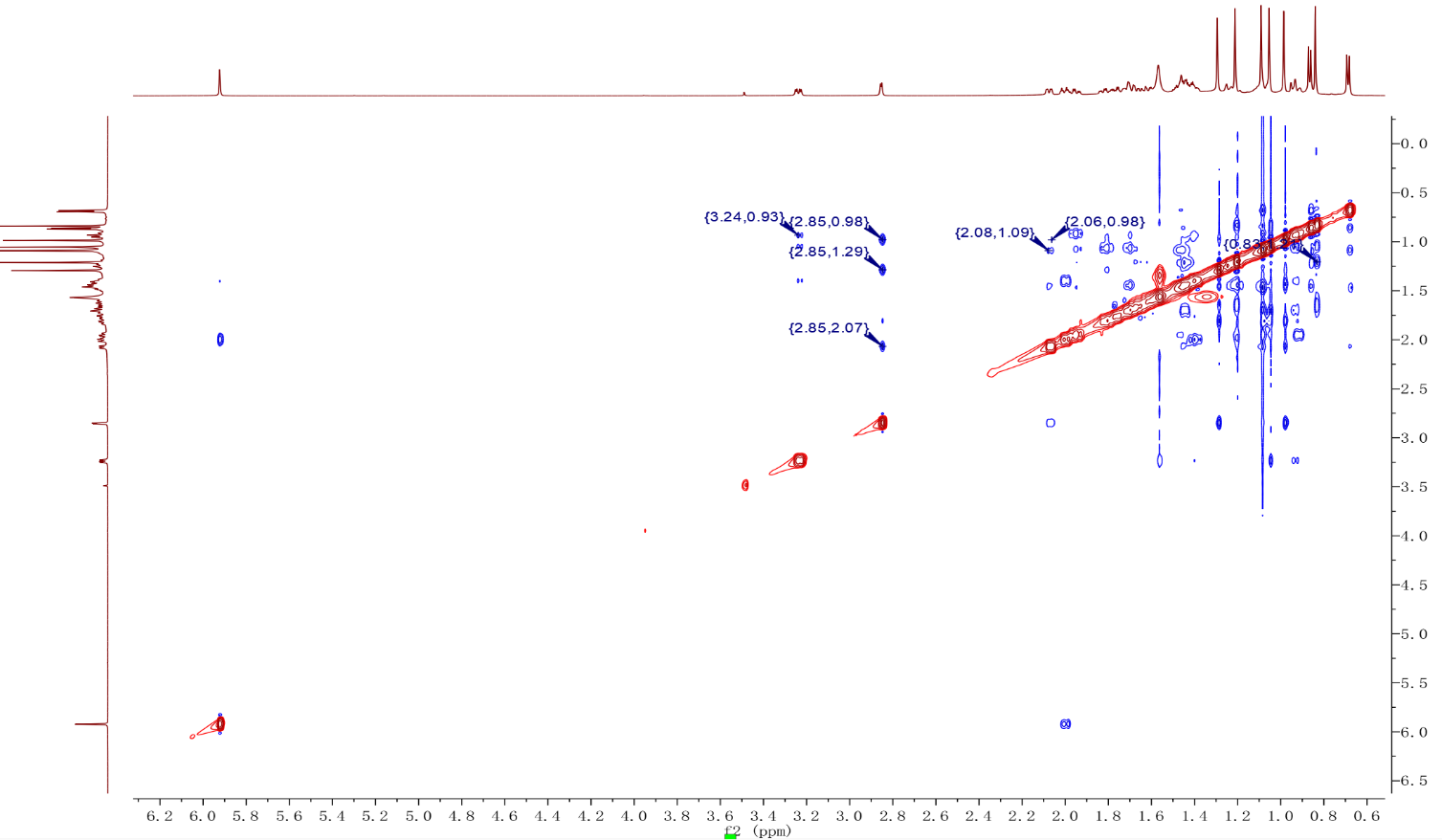


# Experimental ECD spectra of compounds 2, 7 and 10


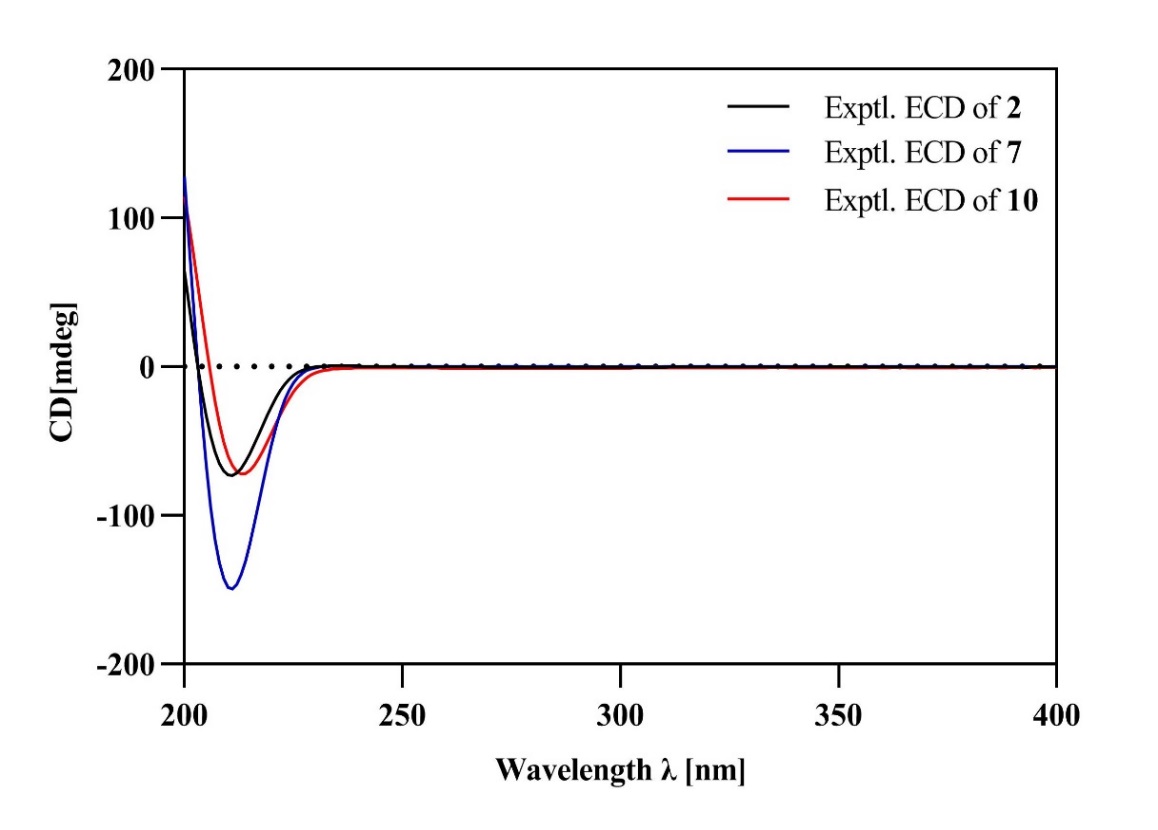


# Relative inhibition curves of compounds 14, 15, 18, 20, 27, 28, 30 and positive control

**
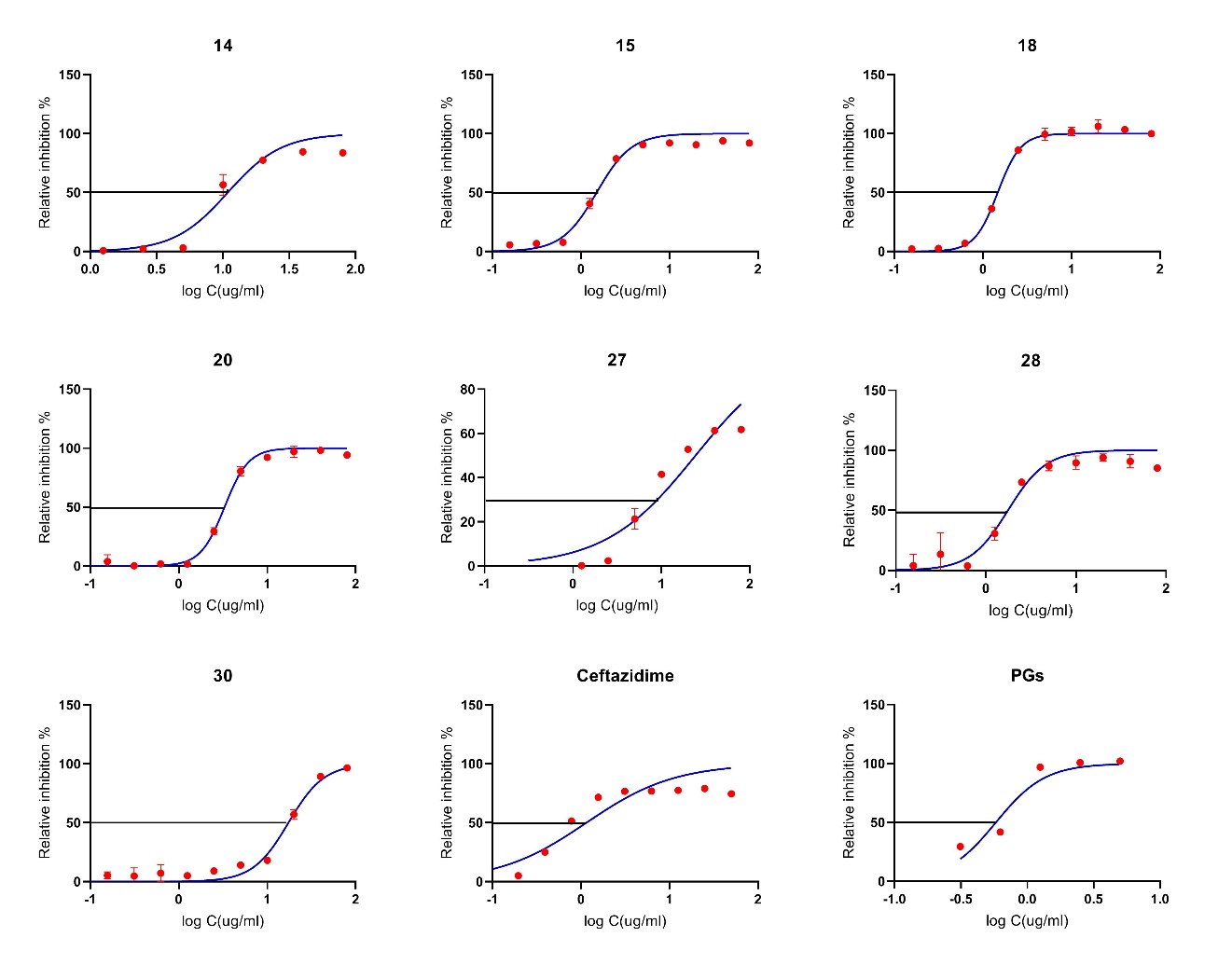
**

# Relative inhibition rate of EE subfractions against *Staphylococcus aureus*

**
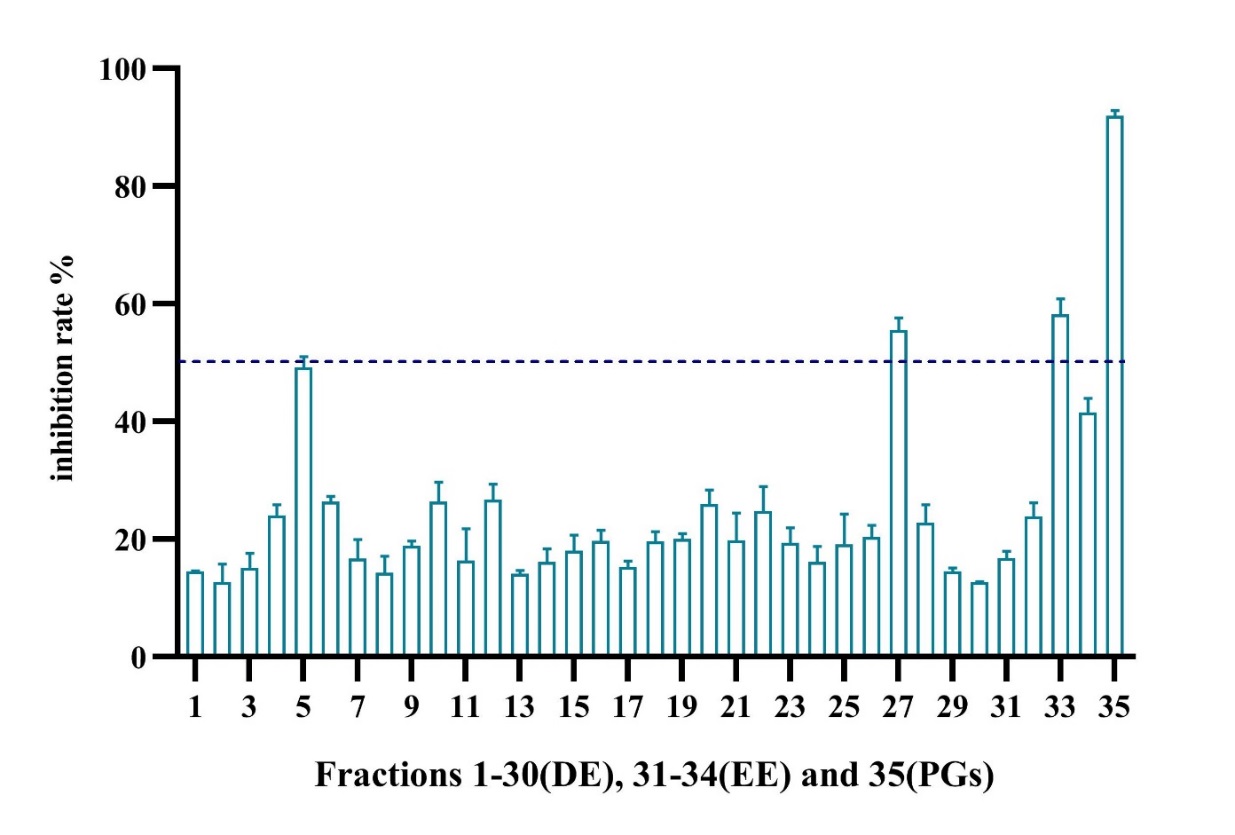
**

# X-ray crystallographic data

Crystallographic data of **1**: C_20_H_30_O_4_ (*M* = 334.44 g/mol): orthorhombic, space group *P*2_1_2_1_2_1_, *a* = 8.30166(13) Å, *b* = 12.78240(18) Å, *c* = 16.6859(3)Å, *V* = 1770.63(5) Å^3^, *Z* = 4, *T* = 302.90(10) K, *μ*(CuKα) = 0.685 mm^-1^, *Dcalc* = 1.255 g/cm^3^, 12586 reflections measured, 3464 unique (*R*_int_ = 0.0229, *R*_sigma_ = 0.0214) which were used in all calculations. The final *R*_1_ = 0.0326 [*I* > 2*σ*(I)], final w*R*_2_ = 0.0863 [*I* > 2*σ*(I)]. The final *R*_1_ = 0.0362 (all data), final w*R*_2_ = 0.0884 (all data). The goodness of fit on *F*^2^ was 1.069, Flack parameter = 0.06(7).

Crystallographic data of **5**: C_20_H_28_O_4_ (*M* = 332.42 g/mol): monoclinic, space group *P*2_1_, *a* = 7.5347(4) Å, *b* = 11.0700(5) Å, *c* = 11.1607(6) Å, *V* = 911.50(8) Å^3^, *Z* = 2, *T* = 100.15 K, *μ*(CuKα) = 0.665 mm^-1^, *Dcalc* = 1.211 g/cm^3^, 5181 reflections measured, 2504 unique (*R*_int_ = 0.1724, *R*_sigma_ = 0.1104) which were used in all calculations. The final *R*_1_ = 0.1003 [*I* > 2*σ*(I)], final w*R*_2_ = 0.2809 [*I* > 2*σ*(I)]. The final *R*_1_ = 0.1257 (all data), final w*R*_2_ = 0.3169 (all data). The goodness of fit on *F*^2^ was 1.178, Flack parameter = 0.2(8).

Crystallographic data of **7**: C_44_H_64_O_10_ (2*M* = 752.95 g/mol): monoclinic, space group *P*2_1_, *a* = 12.6761(3) Å, *b* = 13.5324(2) Å, *c* = 13.3933(3) Å, *V* = 2052.98(9) Å^3^, *Z* = 2, *T* = 297.30(10) K, *μ*(CuKα) = 0.686 mm^-1^, *Dcalc* = 1.218 g/cm^3^, 28798 reflections measured, 8016 unique (*R*_int_ = 0.0252, *R*_sigma_ = 0.0198) which were used in all calculations. The final *R*_1_ = 0.0330[*I* > 2*σ*(I)], final w*R*_2_ = 0.0881 [*I* > 2*σ*(I)]. The final *R*_1_ = 0.0351 (all data), final w*R*_2_ = 0.0894 (all data). The goodness of fit on *F*^2^ was 1.604, Flack parameter = 0.02(5).

Crystallographic data of **8**: C_22_H_32_O_5_ (*M* = 376.47 g/mol): monoclinic, space group *P*2_1_, *a* = 12.4525(4) Å, *b* = 13.2919(4) Å, *c* = 13.6724(4) Å, *V* = 2015.79(11) Å^3^, *Z* = 4, *T* = 170 K, *μ*(CuKα) = 0.698 mm^-1^, *Dcalc* = 1.241 g/cm^3^, 21476 reflections measured, 7522 unique (*R*_int_ = 0.0310, *R*_sigma_ = 0.0364) which were used in all calculations. The final *R*_1_ = 0.0384[*I* > 2*σ*(I)], final w*R*_2_ = 0.1034 [*I* > 2*σ*(I)]. The final *R*_1_ = 0.0393 (all data), final w*R*_2_ = 0.1043 (all data). The goodness of fit on *F*^2^ was 1.035, Flack parameter = 0.14(6).

Crystallographic data of **9**: C_20_H_30_O_4_ H_2_O (*M* = 352.45 g/mol): orthorhombic, space group *P*2_1_2_1_2_1_, *a* = 17.2190(5) Å, *b* = 11.38109(3) Å, *c* = 9.7764(3) Å, *V* = 1915.89(10) Å^3^, *Z* = 4, *T* = 299.94(10) K, *μ*(CuKα) = 0.697 mm^-1^, *Dcalc* = 1.218 g/cm^3^, 10016 reflections measured, 3481 unique (*R*_int_ = 0.0161, *R*_sigma_ = 0.0169) which were used in all calculations. The final *R*_1_ = 0.0555[*I* > 2*σ*(I)], final w*R*_2_ = 0.1614 [*I* > 2*σ*(I)]. The final *R*_1_ = 0.0570 (all data), final w*R*_2_ = 0.1639 (all data). The goodness of fit on *F*^2^ was 1.101, Flack parameter = 0.03(8).

Crystallographic data of **12**: C_20_H_28_O_4_ (*M* = 332.20 g/mol): orthorhombic, space group *P*2_1_2_1_2_1_, *a* = 6.7557(5) Å, *b* = 15.3389(11) Å, *c* = 17.2985(13) Å, *V* = 1792.6(2) Å^3^, *Z* = 4, *T* = 100.15 K, *μ*(CuKα) = 0.677 mm^-1^, *Dcalc* = 1.235 g/cm^3^, 13042 reflections measured, 3533 unique (*R*_int_ = 0.0622, *R*_sigma_ = 0.0638) which were used in all calculations. The final *R*_1_ = 0.0494[*I* > 2*σ*(I)], final w*R*_2_ = 0.1290 [*I* > 2*σ*(I)]. The final *R*_1_ = 0.0753 (all data), final w*R*_2_ = 0.1554 (all data). The goodness of fit on *F*^2^ was 1.199, Flack parameter = – 0.14(16).

Crystallographic data of **13**: C_32_H_54_O_3_ (*M* = 486.75 g/mol): orthorhombic, space group *P*2_1_2_1_2_1_, *a* = 6.7259(3) Å, *b* = 13.3970(8) Å, *c* = 30.7968(18) Å, *V* = 2775.00(3) Å^3^, *Z* = 4, *T* = 100.00(10) K, *μ*(CuKα) = 0.551 mm^-1^, *Dcalc* = 1.165 g/cm^3^, 14060 reflections measured, 5173 unique (*R*_int_ = 0.0615, *R*_sigma_ = 0.0656) which were used in all calculations. The final *R*_1_ was 0.0562 [*I* > 2*σ*(I)], final w*R*_2_ = 0.1383 [*I* > 2*σ*(I)]. The final *R*_1_ = 0.0740 (all data), final w*R*_2_ = 0.1576 (all data). The goodness of fit on *F*^2^ was 1.083, Flack parameter = – 0.2(2).

Crystallographic data of **15**: C_60_H_96_O_4_ (2*M* = 881.36 g/mol): monoclinic, space group *P*2_1_, *a* = 19.1269(4) Å, *b* = 6.6199(2) Å, *c* = 20.1134(5) Å, *V* = 2544.72(11) Å^3^, *Z* = 2, *T* = 100.00(10) K, *μ*(CuKα) = 0.524 mm^-1^, *Dcalc* = 1.150 g/cm^3^, 15090 reflections measured, 7272 unique (*R*_int_ = 0.1273, *R*_sigma_ = 0.1047) which were used in all calculations. The final *R*_1_ = 0.0781[*I* > 2*σ*(I)], final w*R*_2_ = 0.2109 [*I* > 2*σ*(I)]. The final *R*_1_ = 0.1036 (all data), final w*R*_2_ = 0.2346 (all data). The goodness of fit on *F*^2^ was 1.079, Flack parameter = 0.40(5).
